# Divalent Naphthalene Diimide Ligands Display High Selectivity for the Human Telomeric G‐quadruplex in K^+^ Buffer

**DOI:** 10.1002/chem.201700140

**Published:** 2017-03-30

**Authors:** Steven T. G. Street, Donovan N. Chin, Gregory J. Hollingworth, Monica Berry, Juan C. Morales, M. Carmen Galan

**Affiliations:** ^1^ School of ChemistryUniversity of BristolCantock's CloseBristolBS8 1TSUK; ^2^ School of PhysicsUniversity of Bristol, HH Wills Physics LaboratoryBristolBS8 1TLUK; ^3^ Novartis Institutes for Biomedical Research250 Massachusetts Ave.CambridgeMassachusetts02139USA; ^4^ Novartis Institutes for Biomedical Research, Novartis Campus4002BaselSwitzerland; ^5^ Instituto de Parasitología y BiomedicinaAvenida del Conocimiento, s/n18016Armilla, GranadaSpain

**Keywords:** antitumour agents, carbohydrates, drug design, G-quadruplexes, molecular recognition

## Abstract

Selective G‐quadruplex ligands offer great promise for the development of anti‐cancer therapies. A novel series of divalent cationic naphthalene diimide ligands that selectively bind to the hybrid form of the human telomeric G‐quadruplex in K^+^ buffer are described herein. We demonstrate that an imidazolium‐bearing mannoside‐conjugate is the most selective ligand to date for this quadruplex against several other quadruplex and duplex structures. We also show that a similarly selective methylpiperazine‐bearing ligand was more toxic to HeLa cancer cells than doxorubicin, whilst exhibiting three times less toxicity towards fetal lung fibroblasts WI‐38.

G‐quadruplexes are four‐stranded secondary structures that occur in guanine‐rich regions of DNA and are over‐represented in telomeres and gene promoter regions including oncogenes and tumor suppressors.^1^ It has been proposed that stabilization of these polynucleotide sequences by small molecules could lead to novel anticancer treatments.^2^ However, the development of anti‐cancer, drug‐like, bioavailable ligands to selectively stabilize a specific G‐quadruplex structure is still a major challenge, given the approximately 716,310 putative G‐quadruplex forming sequences in the human genome.^3^ Another hurdle to G‐quadruplex ligands reaching clinical development is their insufficient drug‐like character.^4^ Although some progress has been made, none of the G‐quadruplex ligands entering clinical trials have succeeded to date.^5^ Several molecules have been reported that can selectively stabilize one quadruplex structure or topology over another.^6^ For instance, telomestatin analogue TOxaPy6c is one of the most selective ligands discovered to date for the human telomeric G‐quadruplex in Na^+^ buffer. Another ligand, *N*‐methyl mesoporphyrin (NMM), can stabilize the human telomeric G‐quadruplex in K^+^ buffer with no observed stabilization in Na^+^ buffer, although it binds to other parallel quadruplexes.6a

Work within the Morales group has shown that carbohydrates can stack onto the G–C base pair, and affect the stability of the G‐quadruplex structure as well as hydrogen bond to purines.^7^ Indeed, a few examples of carbohydrate‐based G‐quadruplex ligands point towards sugars able to bind to grooves and/or loops of the quadruplex.^8^ We thus proposed that glycosides should be both tolerated and useful binding motifs in quadruplex ligand design.

To test our hypothesis, we decided to replace classical charged amine‐derived side chains with charged sugars on the quadruplex ligands. We chose the naphthalene diimide (NDI) scaffold since NDI‐based compounds are some of the most active and highly studied G‐quadruplex ligands to date, such as tetra‐substituted NDI MM41 developed by the Neidle group.^9^ Despite tri‐ and tetra‐substituted NDI ligands having been extensively researched,^10^, ^11^ di‐substituted NDI scaffolds have not yet been fully explored.11a, 12 Herein we report a highly modular synthesis of a small library of di‐substituted NDI ligands bearing charged carbohydrate moieties alongside classical and non‐classical charged groups, and their evaluation as selective G‐quadruplex ligands (Figure 1) and their in vitro cell cytotoxic profile in model systems.

**Figure 1:**
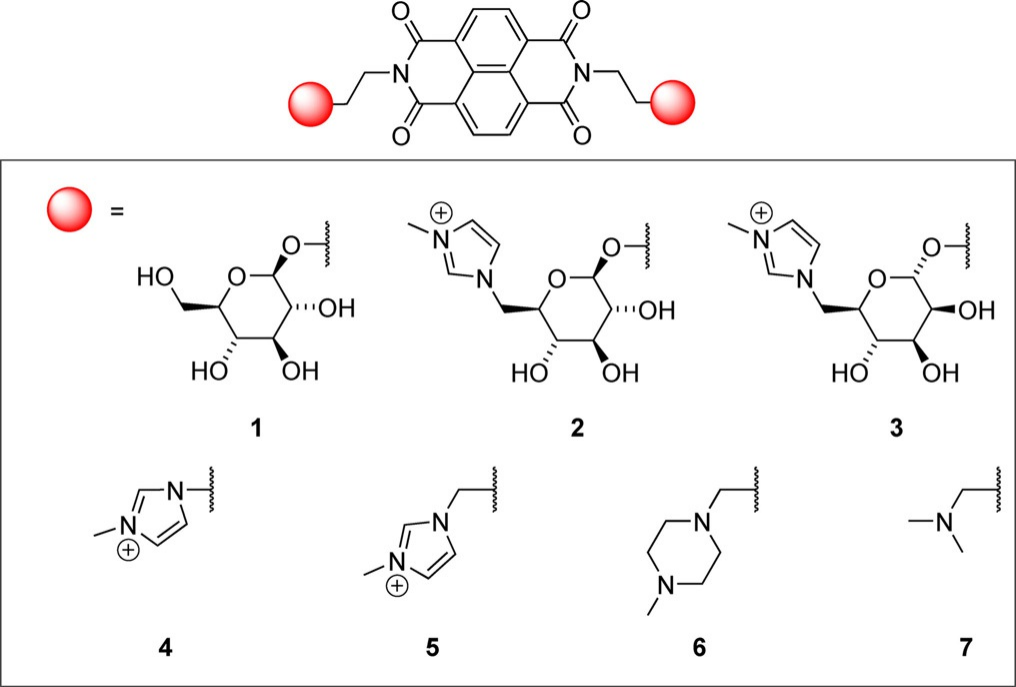
Structure of new NDI ligands **1**–**7**.

Initial efforts focused on targeting glucose‐ and mannose‐based NDIs. We hypothesized that the different stereochemical presentation of the OH at C‐2 and anomeric linkage at C‐1 should yield useful SAR information on the role the carbohydrate scaffold plays in binding and its ability to pick up additional polar interactions. In addition, a methylimidazolium cationic group was introduced at C‐6 of the glycosides to aid binding.^13^ As a control, uncharged glucoside **1** was also targeted, alongside novel NDIs **4** and **5** bearing only the methylimidazolium group in the absence of the sugar but differing in chain length. Finally, di‐substituted methylpiperazine‐ and dimethylamine‐containing **6** and **7** 14 were also prepared, as these motifs had been previously reported as components of other NDI ligands.11a,11b

The synthesis of glyco‐conjugates **2** and **3** was achieved in four steps from azidoalkyl glycosides **8** 15 and **9** 14 (Scheme 1). Selective mesylation of the C6‐hydroxyl using MsCl and pyridine, followed by S_N_2 displacement with methyl imidazole afforded imidazolium‐bearing glycosides **12** and **13** in two steps and 23–33 % overall yield. Subsequent azido reduction by hydrogenolysis followed by condensation with 1,4,5,8‐naphthalenetetra‐carboxylic dianhydride (NTCDA) under basic conditions afforded the desired NDI products **2** and **3**. Analogously, ligands **1** and **4**–**7** were obtained by reaction of NTCDA with either aminoethyl glucoside^14^ or 1‐methylimidazolium amines **S5** and **S6** in ethanol (see the Supporting Information) and 3‐dimethylaminopropylamine^16^ or 1‐(3‐aminopropyl)‐4‐methyl‐piperazine in toluene, respectively.

**Scheme 1:**
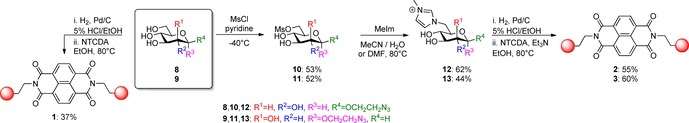
Synthetic route to monosaccharide‐NDIs.

With all the compounds in hand, G‐quadruplex and duplex DNA stabilization was evaluated in a FRET melting assay at a range of concentrations (1–10 μm), (Table 1, Figure 2 and Tables S1–3 in the Supporting Information). Sequences tested were F21T K^+^ (human telomeric G‐quadruplex in K^+^ buffer), F21T Na^+^ (human telomeric G‐quadruplex in Na^+^ buffer), F‐Myc‐T (c‐Myc Pu‐27 G‐quadruplex) and F10T (duplex DNA) (see the Supporting Information). Uncharged glucoside **1** did not stabilize any of the DNA sequences, which is in accordance with earlier observations that cationic charge is needed for quadruplex DNA stabilization. Excitingly, all of the charged ligands **2**–**6** showed different degrees of stabilization of a single G‐quadruplex (F21T K^+^), with no stabilization of the antiparallel F21T Na^+^ quadruplex observed, whilst exhibiting significantly lower stabilization of duplex DNA (F10T). This is somewhat interesting, as it might be expected for smaller, linear compounds such as **6** to be able to intercalate into duplex DNA.^17^ At high concentrations of ligand (e.g. 10 μm), some binding to both the C‐Myc quadruplex and to a lesser extent duplex DNA is observed, however as the concentration of ligand decreases the selectivity for F21T K^+^ increases (see Figure 2). Remarkably, at 1 μm imidazolium‐mannoside **3** and methylpiperazine **6** displayed a 10.1 °C and 9.8 °C stabilization for the F21T sequence in K^+^ buffer, respectively, with no observable binding to any other sequence. Furthermore, mannoside **3** showed a much higher stabilization than glucoside **2**. This could be due to either differences in hydrogen‐bonding interactions (OH at C‐2 in mannose is axial vs. equatorial in glucose), or the relative orientation of the glycoside due to their anomeric linkage (axial vs. equatorial). These compounds, as far as we are aware, are the most selective compounds for the hybrid/parallel type K^+^ human telomeric G‐quadruplex over the antiparallel Na^+^ equivalent, and in addition exhibit the highest selectivity for the hybrid/parallel type K^+^ human telomeric G‐quadruplex over the parallel type c‐Myc promoter quadruplex.

**Table 1:** DNA stabilization in FRET melting assay.^[a]^

Compound	F21T K^+^	F21T Na^+^	F‐Myc‐T	F10T
**1**	0.4±0.1	−0.1±0.5	−0.3±0.4	0.5±0.2
**2**	2.9±0.3	−1.5±0.4	0.9±0.7	0.1±0.2
**3**	10.1±0.3	−1.4±0.3	−0.3±0.7	−0.1±0.1
**4**	1.3±0.4	−0.6±0.5	1±0.4	0.4±0.2
**5**	3.7±0.3	−0.5±0.4	1.8±0.8	0.1±0.1
**6**	9.8±0.6	−2.3±0.3	0.5±0.3	0.3±0.2
**7**	2.3±0.5	−1.5±0.2	3.9±0.2	1.1±0.1

[a] Displayed as Δ*T*_max_ in °C. 1 μm ligand and 200 nm DNA concentration. For further information, see the Supporting Information.

**Figure 2:**
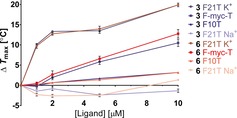
Stabilization of various DNA quadruplex and duplex sequences as a function of concentration by compound **3** and **6**.

Next, we sought to further investigate the quadruplex/duplex DNA selectivity of the most interesting compounds **2**, **3**, **5** and **6**, by using a FRET competition assay with quadruplex forming oligonucleotide F21T K^+^ and unlabeled ds26 competitor duplex DNA (Tables S4–6 and Figures S2–4 in the Supporting Information). In general, the compounds displayed good quadruplex/duplex selectivity, retaining 64–39 % stabilization even at a 1:217 quadruplex:duplex DNA ratio (see Figure S2 in the Supporting Information). It is noteworthy that the quadruplex stabilization effect for glycosides **2** and **3** was less perturbed than for their non‐carbohydrate counterparts (**5** and **6**, Figure S4 in the Supporting Information) suggesting that the former might be more selective towards quadruplex sequences.

To further understand the binding and selectivity observed for compounds **3**, **5** and **6** with the human telomeric G‐quadruplex, circular dichroism (CD) titrations were performed (Figure 3 and Figures S5–S10 in the Supporting Information) with unlabeled human telomeric oligonucleotide telo23 in pH 7.2 and either 100 mm Na^+^ or K^+^ phosphate buffer. All three ligands appeared to cause significant perturbation to the observed CD spectrum, indicating that these compounds are able to induce changes to the conformation of the oligonucleotide. As shown in Figure 3, in K^+^ buffer and at low ligand concentrations (1–3 equiv. of **3**) stabilization of the hybrid‐type topology was observed, as evidenced by the negative band at 235 nm, a reduction in the shoulder at 270 nm and an increase in the maximum at 290 nm.^18^ As the concentration of ligand increases (up to 30 equiv.), the maximum at 290 nm and the shoulder at 270 nm reverses direction. This effect could imply that these ligands appear to stabilize a hybrid‐type topology of telomeric DNA in K^+^ buffer at low concentrations, yet shift to stabilize a parallel‐type topology at higher concentrations, with mannoside **3** exerting the biggest shift in conformation. CD titrations in Na^+^ buffer, where the DNA sequence displays an antiparallel type topology showed a shift towards a hybrid‐type topology (decrease at 240 nm, increase at 260 nm and 295 nm, Supporting Information Figure S5). This would indicate that ligands in question still have some form of interaction with the antiparallel G‐quadruplex, though this does not appear to result in stabilization of the structure in the FRET melting assay. Even in the presence of telo23 without any buffer, **3** was able to induce the formation of a hybrid type G‐quadruplex (decrease in band at 258 nm, increase in band at 288 nm, Supporting Information Figure S10). These results suggest that these NDI ligands can induce significant topological changes to the quadruplex upon binding, and are possibly interacting with a hybrid/parallel‐type conformation of the human telomeric G‐quadruplex.

**Figure 3:**
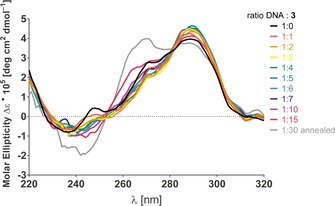
Circular dichroism titration of **3** with telo23 K^+^.

Isothermal titration calorimetry (ITC) was used to obtain quantitative thermodynamic information on the binding of **3**, **5** & **6** to the telo23 K^+^ human telomeric G‐quadruplex (Table 2 and Figures S11–S13 in the Supporting Information. Thermodynamic analysis revealed that the ligands differ in their binding affinity with a *K*_a_ of the order of 10^6^ m ^−1^, with imidazolium **5** having the largest *K*_a_, followed by mannoside **3** and then **6**. Further analysis of the data revealed that enthalpy was the main driving force for binding for all three compounds tested. Entropic contributions were negative in each case, although they were much less for mannoside **3** than for imidazolium **5** or methylpiperazine **6**. Cationic mannoside **3** showed the highest stabilization effect for F21T K^+^ by FRET analysis and also exhibited the second highest Δ*G* values, with the lowest entropic penalty of all three ligands investigated. This suggests that the sugar side chain in the ligand can better displace water molecules from the complex surface than the other motifs. The ITC‐derived binding stoichiometry for **5** and **6** showed approximately two ligands interacting with the quadruplex, whilst the stoichiometry for **3** was 2.7. These differences in binding thermodynamics between the different ligands indicate that besides the NDI scaffold, the side chains play an important role in binding to the oligonucleotide. Furthermore, binding of imidazolium‐glycoside **3**, imidazolium **5** and methylpiperazine **6** to telo23 K^+^ is greater than that of cationic porphyrin TmPyP4 as measured by Bončina et al., and of a similar magnitude as that of the quadruplex ligand Phen‐DC3.^19^

**Table 2:** Thermodynamics of binding of **3**, **5** and **6** to telo23 K^+^ measured by ITC. *K*_a_=association constant, *N*=stoichiometry, Δ*G*=Gibbs free energy change, Δ*H*=enthalpy change, Δ*S*=entropy change and *T*=temperature. Error (in brackets) represents a confidence interval of 68.3 %. For more information, see the Supporting Information.

Compound	**3**	**5**	**6**
*K*_a_ [×10^6^ m ^−1^]	1.30 (1.02,1.64)	2.16 (1.85,2.52)	1.03 (0.865,1.23)
*N*	2.7–2.6	2.0–1.9	1.9–2.0
Δ*G* [kcal/mol]	−8.34	−8.64	−8.20
Δ*H* [kcal/mol]	−10.0 (−8.80, −11.9)	−13.9 (−13.4, −14.4)	−14.5 (−13.7, −15.4)
Δ*S* [cal/mol K]	−5.55	−17.6	−21.0
*T* [K]	298	298	298

Molecular docking simulations were used to try to understand the binding mode of our ligands **2**–**6** with several of G‐quadruplex (g4) structures (parallel, antiparallel and 3+1 hybrid human telomeric g4, and the Pu27T c‐Myc g4) using Molsoft ICM‐Pro^20^ (Figure 4, Supporting Information Tables S7–9 and Figures S14–41). All of the compounds evaluated exhibited the NDI end‐stacking onto the quadruplex, with one side chain pointing into a groove. Models of binding for both **3** and **2** with the parallel human telomeric G‐quadruplex revealed several putative hydrogen bonds and imidazolium stacking interactions within this groove of the quadruplex, with one of the carbohydrate side chains inserted. Compound **6** also displayed a hydrogen‐bonding interaction between the protonated tertiary amine and the phosphate in the groove. The additional interaction of the positively charged imidazolium with the negatively charged phosphate of **3** in the groove is consistent with its enhanced *K*_a_ over **6**. Further work is still needed to fully understand the precise mechanism of cell cytotoxicity for these compounds and whether the observed activity is directly attributed to G‐quadruplex based processes.2a, 21

**Figure 4:**
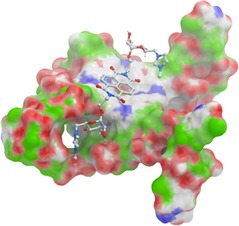
Model of **3** bound to the parallel K^+^ human telomeric G‐quadruplex (based on PDB ID: 4DA3). See the Supporting Information for further details.

In order to evaluate the cell cytotoxicity profile of compounds **1**–**7** and doxorubicin as the benchmark, we compared 72 h incubations with WI‐38 (embryonic human lung fibroblasts), HeLa (human cervical cancer cells), MDA‐MB231 and MCF7 (human breast cancer cells) over the range of 10 fm–100 μm ligand, quantifying the number of live cells (Calcein fluorescence, Table 3) and metabolic competence (Alamarblue, measure of reductive metabolism, Supporting Information Table S10). Whereas glyco‐conjugates **1**–**3** did not appear to be toxic at concentrations up to 100 μm for all the cell cultures tested, all other compounds in the class appeared to exhibit varying degrees of cytotoxicity, with increased toxicity towards cancer cells. HeLa cells were the most susceptible of all the cancer cells screened for example, **6** and **7** displayed 410 and 380 nm IC_50_ values, respectively, a slight improvement in potency when compared to doxorubicin (530 nm). Methylpiperizine **6** displayed the most potent in vitro cancer‐cell killing activity of all the compounds screened (e.g. IC_50_ of 410 nm for HeLa and 810 nm for MDA–MB32 vs. 2.28 μm for the healthy WI‐38 cells) (Table 3). It is important to highlight that whereas doxorubicin displayed a two‐fold selectivity for HeLa over WI‐38, **6** displayed a six‐fold selectivity for HeLa over WI‐38, making it three times as selective. Compounds **4** and **5**, which did not show significant G‐quadruplex stabilization, were about one order of magnitude less toxic to HeLa, MCF‐7 and WI‐38 than **6**. A non‐monotonic dose response was observed when **4** and **5** were tested against MDA‐MB231 cells and IC_50_ values could not be obtained, which suggest a different mode of action.

**Table 3:** Cytotoxicity of **1**–**7** and doxorubicin (Dox) after 72 h incubation.^[a]^

	**WI‐38**	**HeLa**	**MCF7**	**MDA‐MB32**
**Dox**	1.19 (1.05, 1.33)	0.53 (0.42, 0.62)	0.43 (0.37, 0.50)	0.80 (0.66, 0.97)
**1**	>100	>100	>100	>100
**2**	>100	>100	>100	>100
**3**	>100	>100	>100	>100
**4**	21.46 (16.39, 28.10)	6.23 (4.35, 8.92)	52.76 (38.41, 72.48)	n/m
**5**	40.22 (28.83, 56.11)	3.63 (2.59, 5.08)	11.77 (9.67, 14.33)	n/m
**6**	2.28 (2.10, 2.47)	0.41 (0.35, 0.47)	3.09 (2.83, 3.38)	0.81 (0.66, 1.01)
**7**	0.72 (0.67, 0.76)	0.38 (0.32, 0.46)	0.53 (0.48, 0.58)	8.10 (6.73, 9.74)

[a] Absolute IC_50_ values measured in μm using calcein fluorescence to assess live cells, with 95 % confidence interval in brackets. For metabolic effects (Alamar Blue Assay) see the Supporting Information. n/m=Non monotonic dose response observed.

Taking advantage of the inherent fluorescence of our ligands, confocal microscopy was used to assess the intracellular uptake in HeLa cells for compounds **3** and **6** (100 μm) after 30 min. and 16 h incubations. As shown in Figure 5, while significant intracellular uptake is seen for methylpiperazine **6** after 30 min. (Figure 5‐B and 5‐F), the uptake for **3** is much lower (Figure 5‐C and 5‐G), and a further reduction in fluorescence is registered after 16 h (Figure 5‐D and 5‐H). This suggests that the lack of cytotoxicity for glyco‐conjugate **3**, despite showing selective G‐quadruplex binding, could be attributed to poor cellular uptake.^22^ Further work is still needed to fully understand the precise mechanism of cell cytotoxicity for these compounds, and whether the observed activity is directly attributed to G‐quadruplex based processes.2a, 21

**Figure 5:**
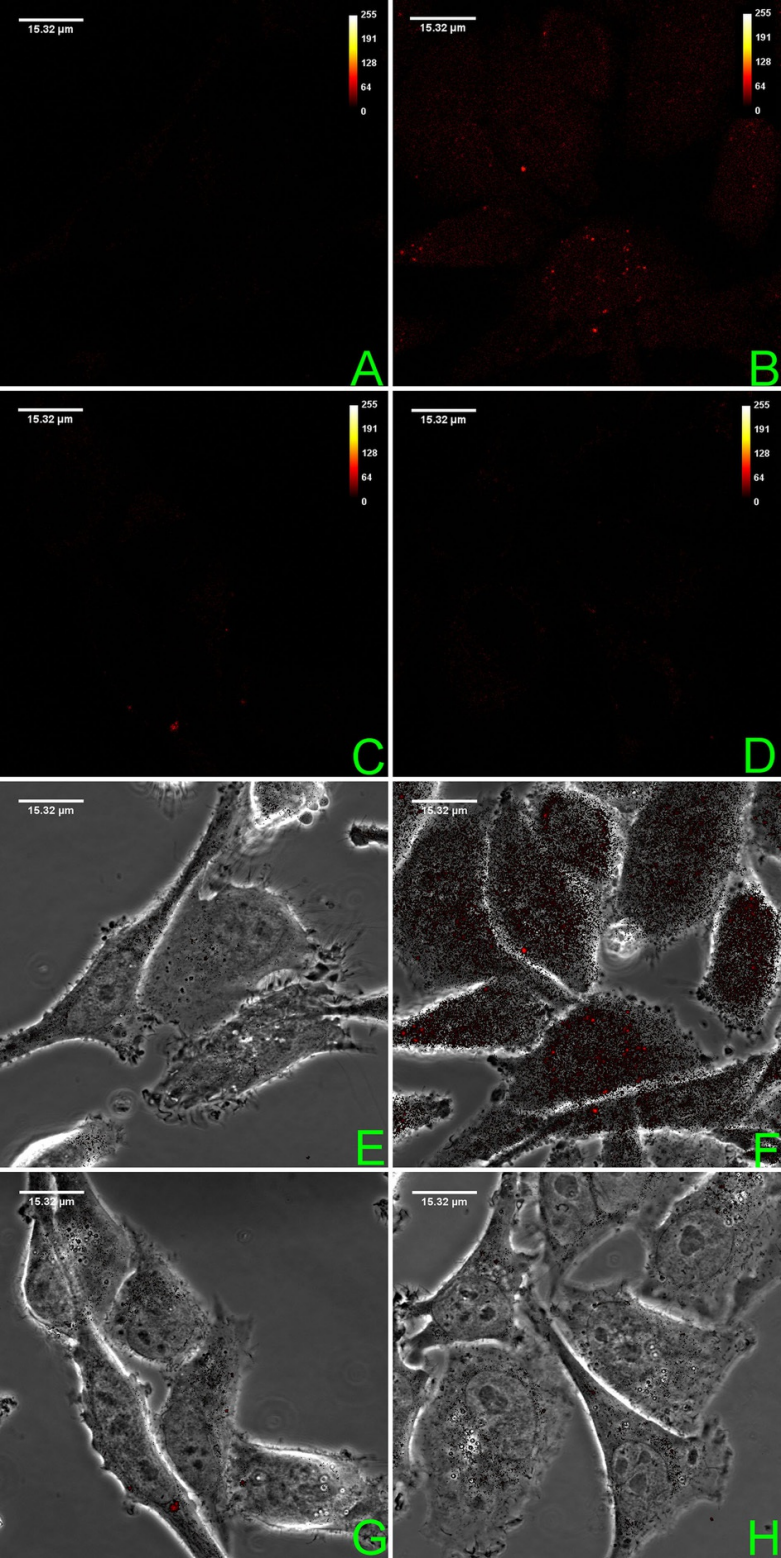
HeLa Cell uptake of **3** and **6** monitored by fluorescence confocal microscopy. Fluorescence images and the merged bright‐field/fluorescence images of: A and E) untreated control; B and F) after 30 min exposure to 100 μm of **6**; C and G) after 30 min exposure to 100 μm of **3**; D and H) after 16 h exposure to 100 μm of **3**. For more information, see the Supporting Information.

In conclusion, the modular synthesis and evaluation of a series of di‐substituted naphthalene diimide G‐quadruplex ligands that display high selectivity towards the hybrid‐type topology of the K^+^ human telomeric G‐quadruplex is described. We showed that imidazolium mannoside **3** is the most selective ligand towards the F21T K^+^ quadruplex sequence to date. Excitingly, compound **6**, which is easily accessible in one step, is more toxic than doxorubicin towards cancer cells whilst exhibiting three times more selectivity. Our findings suggest that lead divalent compounds **3**, **5** and **6** provide an interesting platform for further development and that charged carbohydrates can be exploited as binding motifs that can be tuned to interact with G‐quadruplex grooves. These results represent an exciting step towards developing more selective and bioactive G‐quadruplex ligands with potentially improved anti‐cancer activity.

## Conflict of interest

The authors declare no conflict of interest.

## Supporting information

As a service to our authors and readers, this journal provides supporting information supplied by the authors. Such materials are peer reviewed and may be re‐organized for online delivery, but are not copy‐edited or typeset. Technical support issues arising from supporting information (other than missing files) should be addressed to the authors.

Supplementary
