# Supplementary material for: Divalent Naphthalene Diimide Ligands Display High Selectivity for the Human Telomeric G‐quadruplex in K+ Buffer
Source: Chemistry. 2017 Mar 30;23(29):6953–8. doi: 10.1002/chem.201700140 (PMC5485019; doi:10.1002/chem.201700140)
Supplement: Supplementary file 1 — Supplementary [file CHEM-23-6953-s001.pdf]

CHEMISTRY
A European Journal

Supporting Information

Divalent Naphthalene Diimide Ligands Display High Selectivity for the Human Telomeric G-quadruplex in K^+ Buffer

Steven T. G. Street,[a] Donovan N. Chin,[c] Gregory J. Hollingworth,[d] Monica Berry,[b] Juan C. Morales,*[e] and M. Carmen Galan*[a]

chem_201700140_sm_miscellaneous_information.pdf

Supporting Information

Divalent Naphthalene Diimide Ligands Display High Selectivity for the Human Telomeric G-quadruplex in K^+ Buffer

Steven T. G. Street,[a] Donovan N. Chin,[b] Gregory J. Hollingworth,[c] Monica Berry,[d] Juan C. Morales*,[e] and M. Carmen Galan*,[a]

[a] School of Chemistry, University of Bristol, Cantock's Close, Bristol, BS8 1TS, United Kingdom.

[b] Novartis Institutes for Biomedical Research, 250 Massachusetts Ave., Cambridge, Massachusetts 02139, USA

[c] Novartis Institutes for Biomedical Research, Novartis Campus, CH-4002, Basel, Switzerland

[d] School of Physics, University of Bristol, HH Wills Physics Laboratory, Bristol, BS8 1TL, United Kingdom

[e] Instituto de Parasitología y Biomedicina, Avenida del Conocimiento, s/n 18016, Armilla, Granada, Spain

Table of Contents

General Experimental Details 3
FRET Melting Assays 6
Circular Dichroism 11
Isothermal Titration Calorimetry (ITC) 15
Molecular Docking Studies 20
Cell Culture Protocols 37
Toxicity Assays 37
Confocal Microscopy 43
Experimental Procedures and Data 46
NMR Spectra of Novel Compounds 57
References 71

General Experimental Details

Chemicals were purchased and used without further purification. All oligonucleotides used were purchased from Eurogentec (Belgium), purified by HPLC and delivered dry. Oligonucleotide concentrations were determined by UV-absorbance using a NanoDrop 2000 Spectrophotometer from Thermo Scientific. Dry solvents were obtained by distillation using standard procedures, or by passage through a column of anhydrous alumina using equipment from Anhydrous Engineering (University of Bristol) based on the Grubbs' design.[1] Reactions requiring anhydrous conditions were performed under N_2; glassware and needles were either flame dried immediately prior to use, or placed in an oven (150 °C) for at least 2 h and allowed to cool in a desiccator or under reduced pressure. Liquid reagents, solutions or solvents were added via syringe through rubber septa; solid reagents were added via Schlenk type adapters. Reactions were monitored by TLC on Kieselgel $60F_{254}$ (Merck), with UV light (254 or 365 nm) detection and either by charring with 10% sulfuric acid in ethanol, staining with ninhydrin or staining with PPh_3 and then ninhydrin. Flash column chromatography was performed according to Still and co-workers,[2] using silica gel [Merck, 230−400 mesh (40−63 μm)]. The crude material was applied to the column by pre-adsorption onto silica, as appropriate. Reverse phase flash chromatography was performed on a Teledyne Isco Combiflash purification system, with RediSep Rf Gold C18 cartridges. Solvents for flash column chromatography (FCC) and thin layer chromatography (TLC) are listed in volume:volume percentages. Extracts were concentrated in vacuo using both a Heidolph Hei-VAP Advantage rotary evaporator (bath temperatures up to 50 °C) at a pressure of 15 mmHg (diaphragm pump) or 0.1 mmHg (oil pump), as appropriate, and a high vacuum line at room temperature. Water soluble compounds were freeze dried on a Lytotrap Plus (LTE Scientific LTD). ^{1}H NMR and ^{13}C NMR spectra were measured at 25°C in the solvent specified with Varian or Bruker spectrometers operating at field strengths listed. Chemical shifts are quoted in parts per million with spectra referenced to the residual solvent peaks. Multiplicities are abbreviated as: br (broad), s (singlet), d (doublet), t (triplet), q (quartet), p (pentet), m (multiplet) and *app.* (apparent) or combinations thereof. Assignments of ^{1}H NMR and ^{13}C NMR signals were made where possible, using COSY, HSQC and HMBC experiments. ^{19}F NMR spectra were recorded with 2-fluorobenzoic acid present as an internal standard to quantify the amount of TFA. For TFA quantification, the integration of the internal standard was compared with TFA in ^{19}F NMR and the cation in ^{1}H NMR and the ratio between the

two taken. Compounds **6** and **7** were tested as their protonated TFA salts. Carbohydrate numbering nomenclature in pyranoside systems follows Figure S1 below.

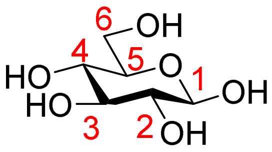

Figure S1. Nomenclature for numbering of pyranoside systems

Mass spectra were obtained by the University of Bristol mass spectrometry service by electrospray ionisation (ESI) or matrix assisted laser desorption ionisation (MALDI) modes. Reactions followed by MALDI were analysed using an Applied Biosystems 4700 Proteomics analyser time-of-flight/time-of-flight mass spectrometer. Infra-red spectra were recorded in the range 4000-650 cm^{-1} on a Perkin Elmer Spectrum either as neat films or solids compressed onto a diamond window. Optical rotations were measured on a Bellingham + Stanley ADP220 polarimeter. The units of the specific rotation, (deg·mL)/g·dm) are implicit and are not included with the reported value. Concentration *c* is given in g/100 mL.

Analytical high performance liquid chromatography (HPLC) was performed on a Waters system with a Waters 2707 Autosampler, a Waters 2535 Quaternary Gradient Module, a Waters In-Line Degasser, a Waters Temperature Control Module II, a Waters 2424 ELS Detector and a Waters 2998 Photodiode Array Detector (PDA) set to monitor 210 – 450 nm. Compound purity was assessed by monitoring the PDA at the wavelength specified, using the conditions as follows: The analytical column used was a Phenomenex Luna 3 µm C18(2) 100 Å (250 × 4.6 mm). For the analytical method, the flow rate was 0.75 mL / min and the mobile phases used were 0.05 % formic acid in water for the aqueous phase and 0.05 % formic acid in methanol for the organic phase. The gradient was 5 % organic phase for 5 minutes at the start before increasing to 40 % organic phase over 25 minutes, then increasing to 95 % organic phase over 1 minute before holding at 95 % for 9 minutes.

Analytical Liquid Chromatography-Mass Spectrometry (LC-MS) was performed on a Waters Acquity Ultra Performance LC-MS with PDA detector, set to monitor 100-1600 m/z on the MS and 210 – 450 nm on the PDA. The column used was a Waters Acquity UPLC HSS T3 1.8 µm (2.1 × 50 mm). For the method, the flow rate was 1 mL / min, the mobile phases used were 0.05 % formic acid and 0.05 % ammonium acetate in water for the aqueous phase and 0.04 % formic acid in acetonitrile for the organic phase. The gradient was from 5 % organic phase at the start to 98 % organic phase over 9.4 minutes, then holding at 98 % organic for

0.4 minutes before returning to 5 % organic over 0.1 minutes and holding at 5 % organic for 0.3 minutes.

Preparative HPLC was performed on a Grace Discovery Sciences Reveleris Prep System with a Phenomenex Luna 5 µm C18(2) 100 Å AXIA packed (250 × 21.2 mm) column. For purification, the instrument was set to monitor the ELSD signal as well as 220 nm, 254 nm and 280 nm light on the UV detector. Flow rates were 14 mL / min. The mobile phases used were 0.05 % formic acid in water for the aqueous phase and 0.05 % formic acid in methanol for the organic phase. The gradient was from 5 % organic phase for 5 minutes at the start to 40 % organic phase over 10 minutes, then holding at 40 % organic phase for 10 minutes before rising to 80 % organic phase over 10 minutes, with 10 minutes at 100 % organic phase followed by 5 minutes at 5 % organic phase. The fractions were combined and concentrated in the same manner as for FCC.

FRET Melting Assays

FRET melting assays were performed according to the procedure reported by De Cian and co-workers[3] on a Stratagene MX3005P qPCR. The oligonucleotides used were:

Quadruplex Model	Sequence
F21T (Human Telomeric G-quadruplex)	5’-FAM-GGGTTAGGGTTAGGGTTAGGG-TAMRA-3’
FMycT (c-Myc Pu27 promoter quadruplex)	5’-FAM-TTGAGGGTGGGTAGGGTGGGTAA-TAMRA-3’
F10T (Duplex DNA)	5’-FAM-TATAGCTATA-*HEG*-TATAGCTATA-TAMRA-3’
ds26 (unlabelled competitor duplex DNA)	5'-CAATCGGATCGAATTCGATCCGATTG-3'

Where FAM = 6-carboxyfluorescein, TAMRA = 6-carboxy-tetramethylrhodamine and HEG = $[(-CH_2CH_2O-)_6]$

All ligands were annealed before use by heating for 2 minutes at 90°C and then placed immediately into ice. The final concentration of oligonucleotide was 200nM in all cases, except for ds26 which was used in 0.2 µM, 1 µM and 10 µM concentrations as part of the FRET competition assay as stated. The buffer used depended on the sequence in question, for F21T in Na^+ conditions, the final buffer concentration was 100mm NaCl, and 10 mm Li Cacodylate. For FMycT (K^+ Conditions), 1 mM KCl, 99 mM LiCl and 10 mM Li Cacodylate was used. For F21T in K^+ conditions as well as for F10T, 10 mM KCl, 90 mM LiCl and 10 mM Li Cacodylate was used. Ligand concentrations were either 1 µM, 2 µM, 5 µM or 10 µM. FRET competition assays used the same buffer as their non-competition counterparts, and the ratio of quadruplex/duplex DNA was taken as the ratio of G-quartets to nucleotide base pairs. Each sample was tested in duplicate, and each experiment was tested in at least triplicate to assess the reproducibility of all results. Appropriate control experiments were also carried out for each sample set, using the ligand TmPyP4 as a positive control. Data processing was carried out using Origin 9, with ΔT_{max} used to represent ΔT_m.

Table S1. DNA Stabilization of Ligands in FRET Melting Assay at 10µM Concentration (ΔT_{max} °C).

Compound	F21T K^+	F21T Na^+	F-myc-T	F10T
**1**	0.4 ± 0.3	-0.5 ± 0.3	-0.1 ± 0.4	0.0 ± 0.1
**2**	14.1 ± 0.5	-1.5 ± 0.1	8.0 ± 0.8	1.8 ± 0.1
**3**	19.9 ± 0.4	-1.3 ± 0.4	10.5 ± 0.9	3.2 ± 0.0
**4**	8.2 ± 0.6	0.1 ± 0.9	8.3 ± 0.8	2.0 ± 0.1
**5**	14.7 ± 0.4	1.0 ± 0.4	14.4 ± 0.9	4.1 ± 0.3
**6**	19.9 ± 0.3	1.3 ± 0.2	12.8 ± 1.0	3.2 ± 0.0
**7**	13.2 ± 0.4	0.9 ± 0.3	16.1 ± 0.4	5.7 ± 0.2

Table S2. DNA Stabilization of Ligands in FRET Melting Assay at 5µM Concentration (ΔT_{max} °C).

Compound	F21T K^+	F21T Na^+	F-myc-T	F10T
**1**	-0.4 ± 0.6	-0.6 ± 0.4	0.9 ± 0.4	0 ± 0.1
**2**	9.3 ± 0.6	-1.3 ± 0.5	4.8 ± 0.5	0.9 ± 0.2
**3**	13.5 ± 0.7	-2.4 ± 0.1	5.8 ± 0.7	1.7 ± 0.2
**4**	4.0 ± 0.6	-0.4 ± 0.8	3.9 ± 0.5	1.0 ± 0.2
**5**	10.5 ± 0.7	-1.8 ± 0.5	10.1 ± 0.7	2.3 ± 0.2
**6**	13.9 ± 0.7	-2.5 ± 0.3	6.6 ± 0.6	1.4 ± 0.2
**7**	8.8 ± 0.5	-0.9 ± 0.4	12.7 ± 0.4	3.5 ± 0.2

Table S3. DNA Stabilization of Ligands in FRET Melting Assay at 2µM Concentration (ΔT_{max} °C).

Compound	F21T K^+	F21T Na^+	F-myc-T	F10T
**1**	-0.4 ± 0.4	-0.8 ± 0.7	-0.1 ± 0.2	0.1 ± 0.1
**2**	5.1 ± 0.4	-1.0 ± 0.5	1.3 ± 0.8	0 ± 0.1
**3**	13.2 ± 0.3	-0.8 ± 0.4	2.0 ± 0.6	0.7 ± 0.2
**4**	1.6 ± 0.5	0 ± 0.6	1.5 ± 0.3	0.2 ± 0.3
**5**	6.1 ± 0.5	-1.7 ± 0.5	5.0 ± 0.4	0.9 ± 0.1
**6**	12.8 ± 0.35	-2.6 ± 0.4	2.7 ± 0.9	0.7 ± 0.2
**7**	4.1 ± 0.7	-1.8 ± 0.6	7.3 ± 0.4	2.1 ± 0.1

Table S4. Ligand Stabilization of F21T K^+ DNA in FRET Competition Assay at 5μM Concentration with amount of ds26 added below (ΔT_{max} °C).

Compound	0 μM [ds26]	0.2 μM [ds26]	1 μM [ds26]	10 μM [ds26]
**1**	-	-	-	-
**2**	9.3 ± 0.6	9.9 ± 0.5	9.5 ± 0.7	5.9 ± 0.3
**3**	13.5 ± 0.6	13.0 ± 0.5	12.6 ± 0.3	7.6 ± 0.4
**4**	4.0 ± 0.6	5 ± 0.3	4 ± 0.5	1.1 ± 0.3
**5**	10.5 ± 0.7	10.3 ± 0.8	9.5 ± 0.8	4.1 ± 0.5
**6**	13.9 ± 0.7	13.2 ± 0.8	12.3 ± 0.6	7.2 ± 0.3
**7**	-	-	-	-

Table S5. Ligand Stabilization of F21T K^+ DNA in FRET Competition Assay at 1μM Concentration with amount of ds26 added below (ΔT_{max} °C).

Compound	0 μM [ds26]	0.2 μM [ds26]	1 μM [ds26]	10 μM [ds26]
**1**	-	-	-	-
**2**	-	-	-	-
**3**	10.1 ± 0.3	4.5 ± 0.5	3.9 ± 0.4	1.9 ± 0.4
**4**	-	-	-	-
**5**	3.7 ± 0.3	2.5 ± 0.5	2.3 ± 0.4	0.4 ± 0.7
**6**	9.8 ± 0.6	1.3 ± 0.3	1.1 ± 0.3	-0.6 ± 0.6
**7**	-	-	-	-
**TmPyP4**	27.4 ± 0.4	17.8 ± 0.4	14.4 ± 0.6	8.7 ± 0.6

Table S6. Ligand Stabilization of F-Myc-T DNA in FRET Competition Assay at 5μM Concentration with amount of ds26 added below (ΔT_{max} °C).

Compound	0 μM [ds26]	0.2 μM [ds26]	1 μM [ds26]	10 μM [ds26]
**1**	-	-	-	-
**2**	4.8 ± 0.5	3.4 ± 0.3	3.9 ± 0.3	0.5 ± 0.4
**3**	5.8 ± 0.7	5.7 ± 0.2	5.0 ± 0.2	1.6 ± 0.3
**4**	3.9 ± 0.5	3.9 ± 0.2	2.8 ± 0.2	-0.3 ± 0.3
**5**	10.1 ± 0.7	9.7 ± 0.2	9.0 ± 0.4	2.2 ± 0.3
**6**	6.6 ± 0.6	8.4 ± 0.5	7.2 ± 0.4	2.4 ± 0.1
**7**	-	-	-	-

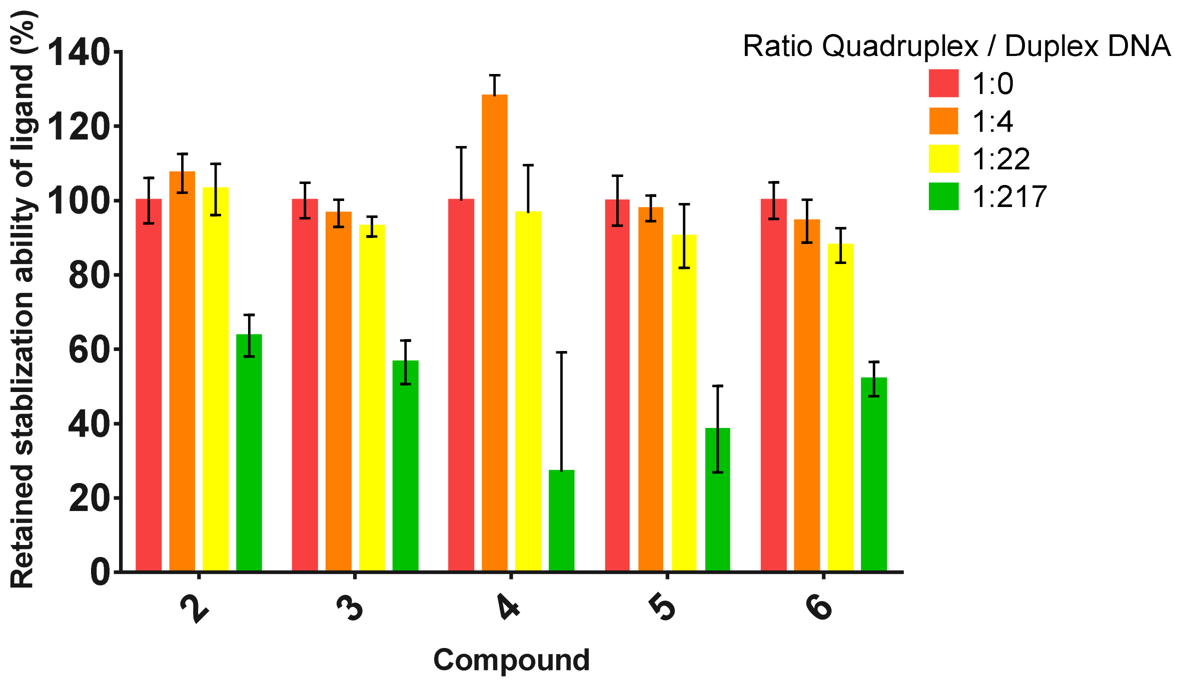

Figure S2. FRET Competition Assay of F21T K^+ at 25:1 (ligand:quadruplex) ratio, with unlabelled ds26 duplex DNA added as a competitor.

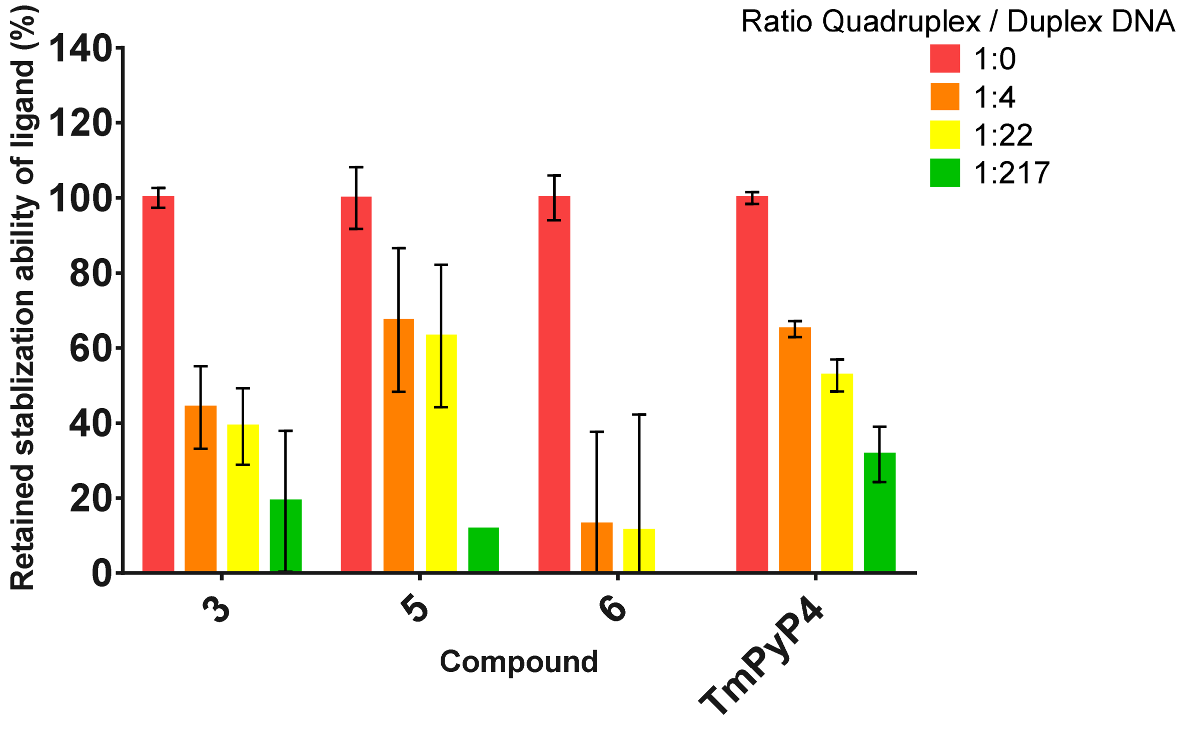

Figure S3. FRET Competition Assay of F21T K^+ at 5:1 (ligand:quadruplex) ratio, with unlabelled ds26 duplex DNA added as a competitor. Note that at very low quadruplex / duplex DNA ratios, **3** is comparable with TmPyP4, while **6** is failing. This emphasises the importance of carbohydrates on increasing specific binding to the K^+ form of the human telomeric G-quadruplex.

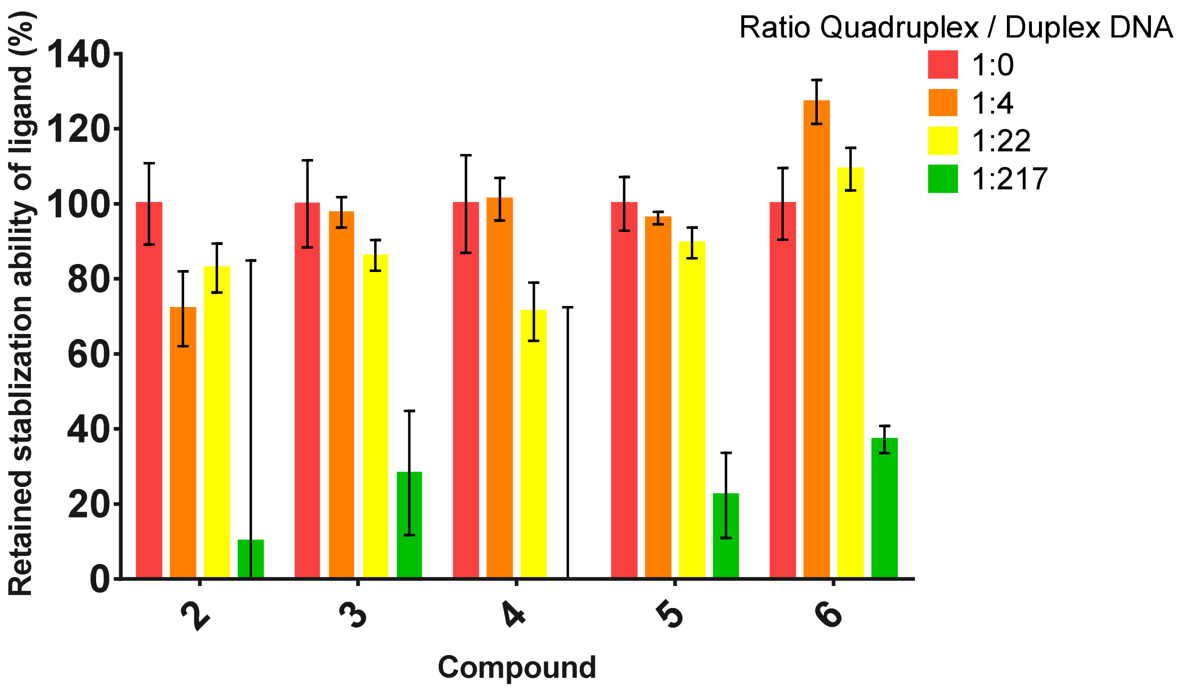

Figure S4. FRET Competition Assay of F-Myc-T at 25:1 (ligand:quadruplex) ratio, with unlabelled ds26 duplex DNA added as a competitor.

Circular Dichroism

Circular Dichroism (CD) titrations were recorded using a Jasco J-810 spectrometer fitted with a Peltier temperature controller. Measurements were taken in a quartz cuvette with a path length of 5 mm, at 20°C, at a 50 nm / min scanning speed at 1 nm intervals, with a 1 nm bandwidth. The CD spectrum was recorded between 450 and 200 nm, and baseline corrected for the buffer used. The oligonucleotide sequence used was:

telo23 (human telomeric G-quadruplex): 5'-TAGGGTTAGGGTTAGGGTTAGGG -3'

The oligonucleotide was at a concentration of 4.22 μM which gave an OD of 1 and the buffer used was either sodium or potassium phosphate (100 mM, pH 7.4). Oligonucleotide concentration remained constant throughout, and dilutions were made using a solution containing both oligonucleotide and the ligand, with the amount added determining the relative ratio of the two species. The reported spectrum for each sample represents the average of 3 scans and is baseline corrected for the buffer and ligand. Data processing was carried out using Prism 6 with a 4 point second order smoothing polynomial applied to all spectra. Observed ellipticities were converted to mean residue ellipticity (θ) = deg x cm^2 x $dmol^{-1}$ (mol ellip).

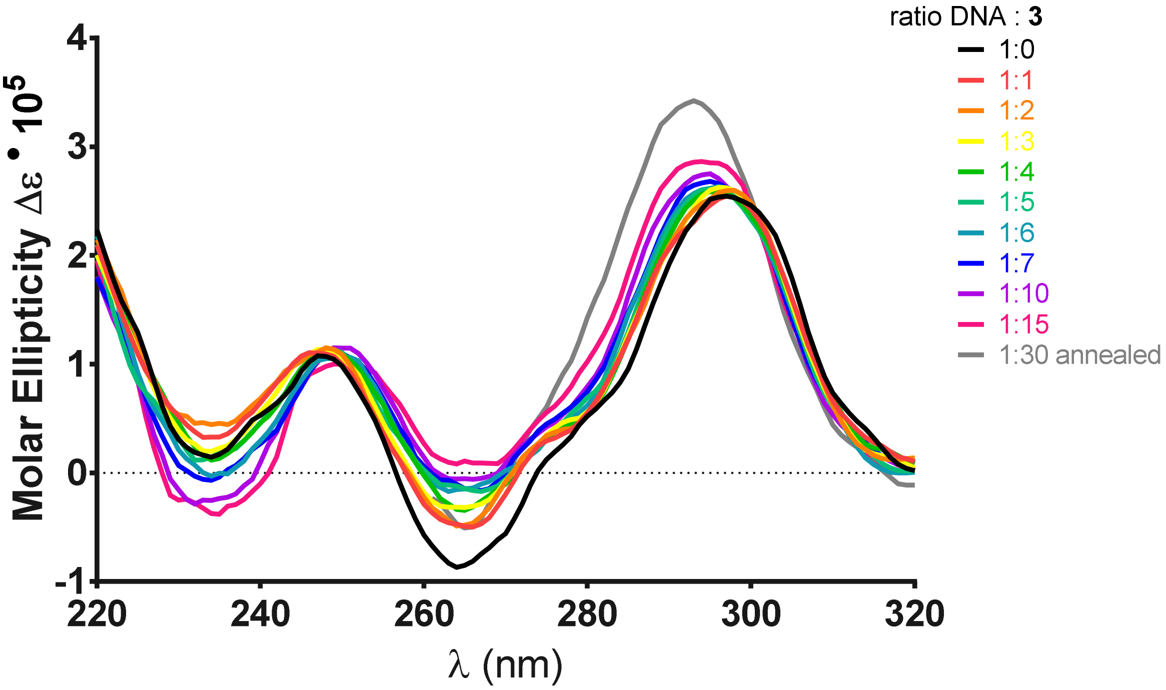

Figure S5. CD titration of **3** with telo23 in Na^+ buffer. The increase in ligand concentration corresponds to a shift towards a mixture of antiparallel and hybrid type topologies (increase in negative band at 265nm and increase / shift in positive band at 295 nm).

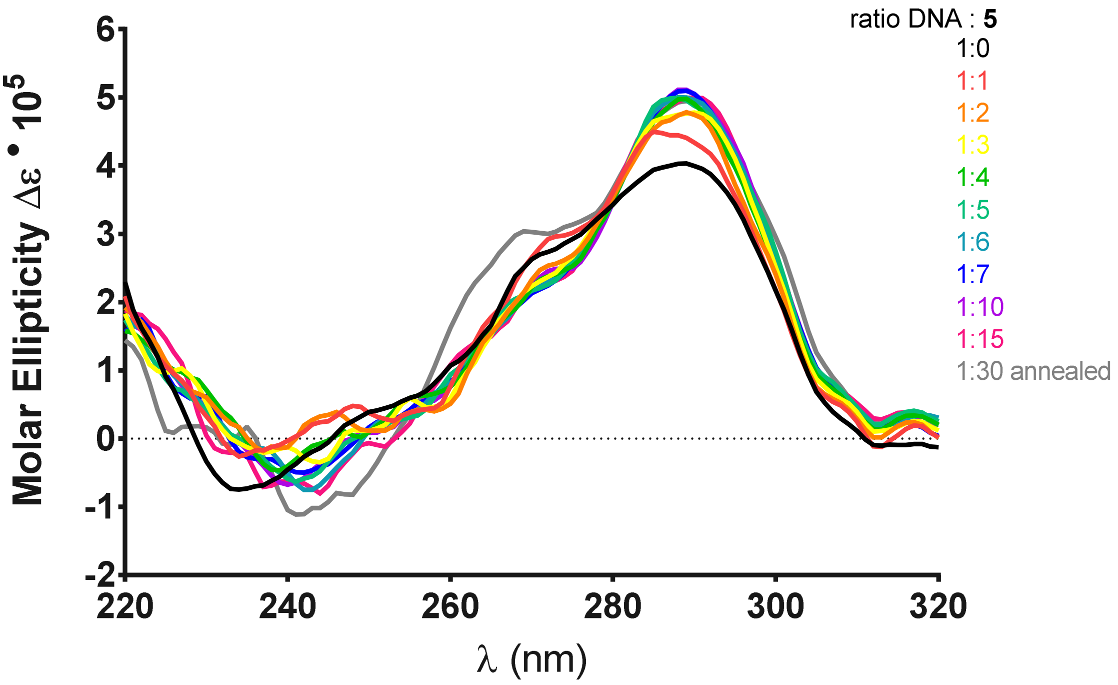

Figure S6. CD titration of **5** with telo23 in K^+ buffer. The increase in ligand concentration corresponds to an increase in hybrid-type topology, similar to **3** but to a lesser extent.

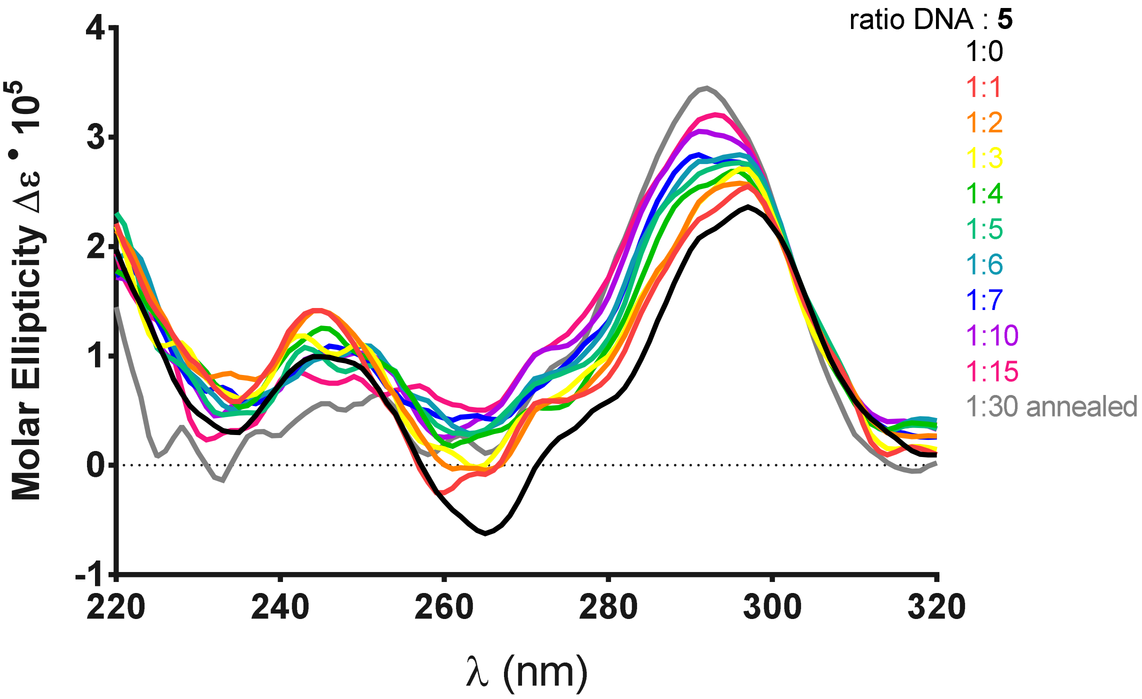

Figure S7. CD titration of **5** with telo23 in Na^+ buffer. The increase in ligand concentration corresponds to a shift towards a mixture of antiparallel and hybrid type topologies (increase in negative band at 265nm and increase / shift in positive band at 295 nm).

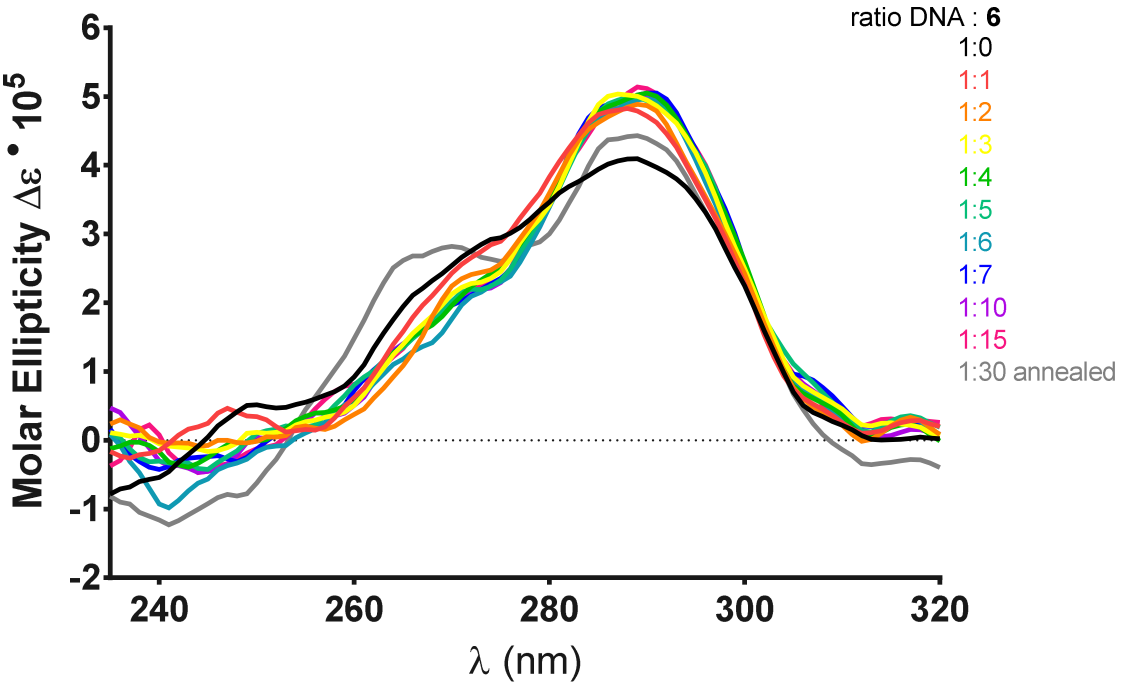

Figure S8. CD titration of **6** with telo23 in K^+ buffer. The increase in ligand concentration corresponds to an increase in hybrid-type topology, similar to **3** but to a lesser extent.

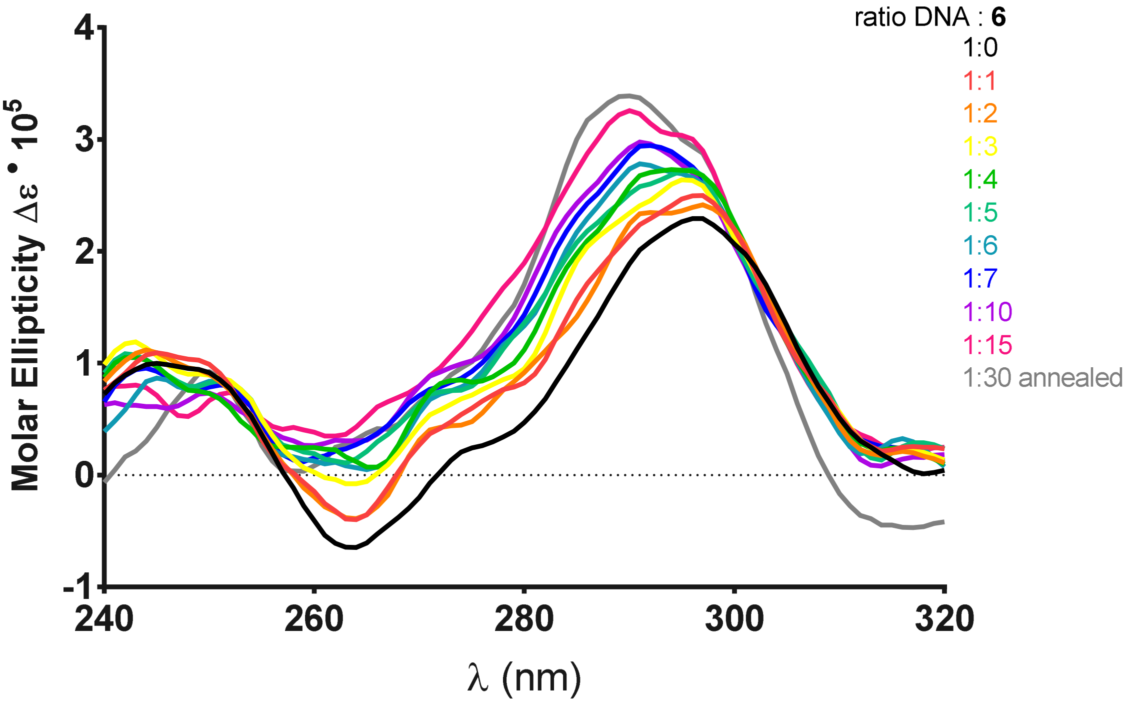

Figure S9. CD titration of **6** with telo23 in Na^{+} buffer. The increase in ligand concentration corresponds to a shift towards a mixture of antiparallel and hybrid type topologies (increase in negative band at 265nm and increase / shift in positive band at 295 nm).

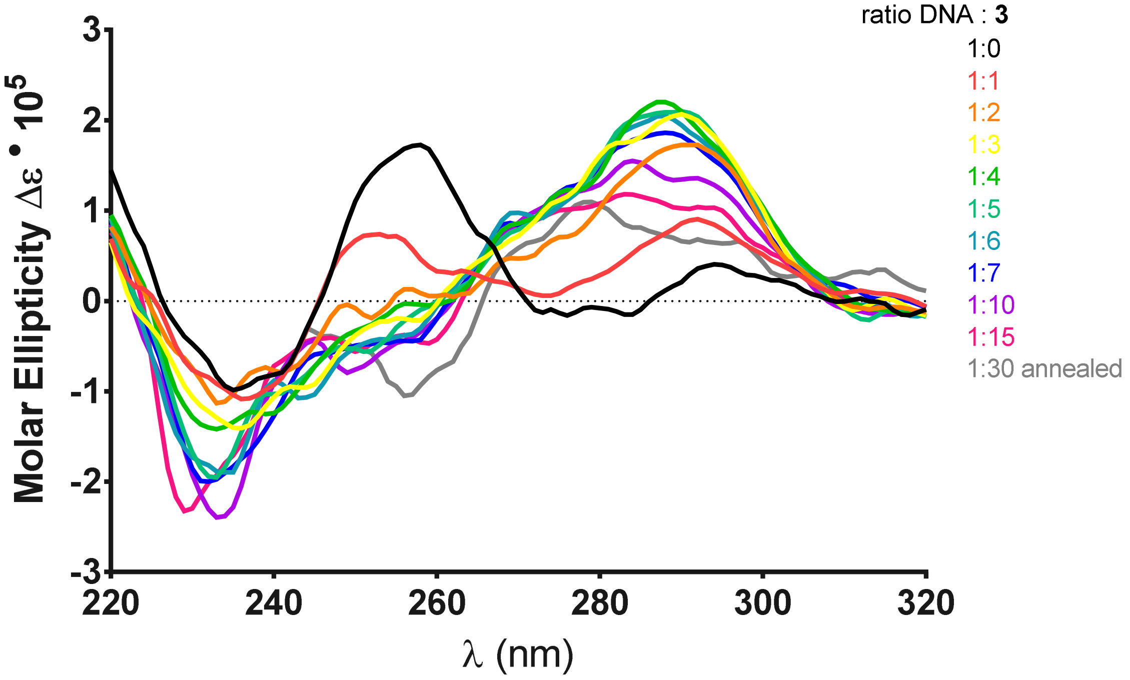

Figure S10. CD titration of **3** with telo23 without buffer (no preformed G-quadruplex). These results suggest the induction of a hybrid-type G-quadruplex topology of the oligonucleotide upon the addition of compound **3**.

Isothermal Titration Calorimetry (ITC)

ITC measurements were carried out in a MicroCal Auto-iTC$_{200}$ titration calorimeter (MicroCal, Northampton, MA).All ITC experiments were carried out in the same buffer (100 mM potassium phosphate, pH 7.4). The oligonucleotide sequence used was:

telo23 (human telomeric G-quadruplex): 5'-TAGGGTTAGGGTTAGGGTTAGGG -3'

The volume of sample in the reference cell was always 200 μL, and the volume of sample in the syringe was always 40 μL. Before loading, all solutions were thoroughly degassed. The reference cell was filled with the degassed buffer. The oligonucleotide was annealed and placed in the sample cell (15 μM), and the respective ligand was dissolved in the same buffer and placed in the syringe (300 μM). 19 injections were made at 25°C, at 10 μcal/sec with an initial delay of 60 seconds. The stirring speed was 750 rpm throughout. The first injection was 0.4 μL for a duration of 0.8 seconds, the next injections were 2 μL for a duration of 4 seconds. The spacing between each injection was 150 seconds, and the filter period was 5 seconds. The heats of dilution were determined by parallel experiments by injecting the respective ligand into the same buffer. Each experiment was performed in duplicate to assess the reproducibility of the result..

The thermograms (integrated heat / injection data) obtained were processed according to the procedure outlined by Brautigam et. al.[4] This consisted of a bias-free, global analysis using freely available NITPIC,[5] SEDPHAT[6] and GUSSI[7] software. Firstly, the thermograms imported and were integrated automatically using NITPIC. The results were imported into SEDPHAT and underwent a global analysis and curve-fitting. Multiple different non-linear regression binding models were trialled, and the simplest binding model that was capable of rationalising the data was selected. In this case, all data was fitted to the 'A+B <-> AB heterogeneous association' model. The association constant (K_a) and enthalpy change (ΔH), were minimised simultaneously and fitted to all datasets as part of the curve fitting process until a minimum was found. These values were then used to calculate the entropy change (ΔS) and Gibbs free energy (ΔG) using the equation $\Delta G = -RTLn\ K_a = \Delta H - T\Delta S$ where R is the gas constant and T is the absolute temperature. Finally, this data was imported into GUSSI to generate the final figures. Error was calculated using the 'automatic confidence interval search with projection method' and used a P value of 0.683. The stoichiometry (N) was calculated by using the 'one-set of sites' binding model in the Microcal Analysis add-on for Origin 9.0

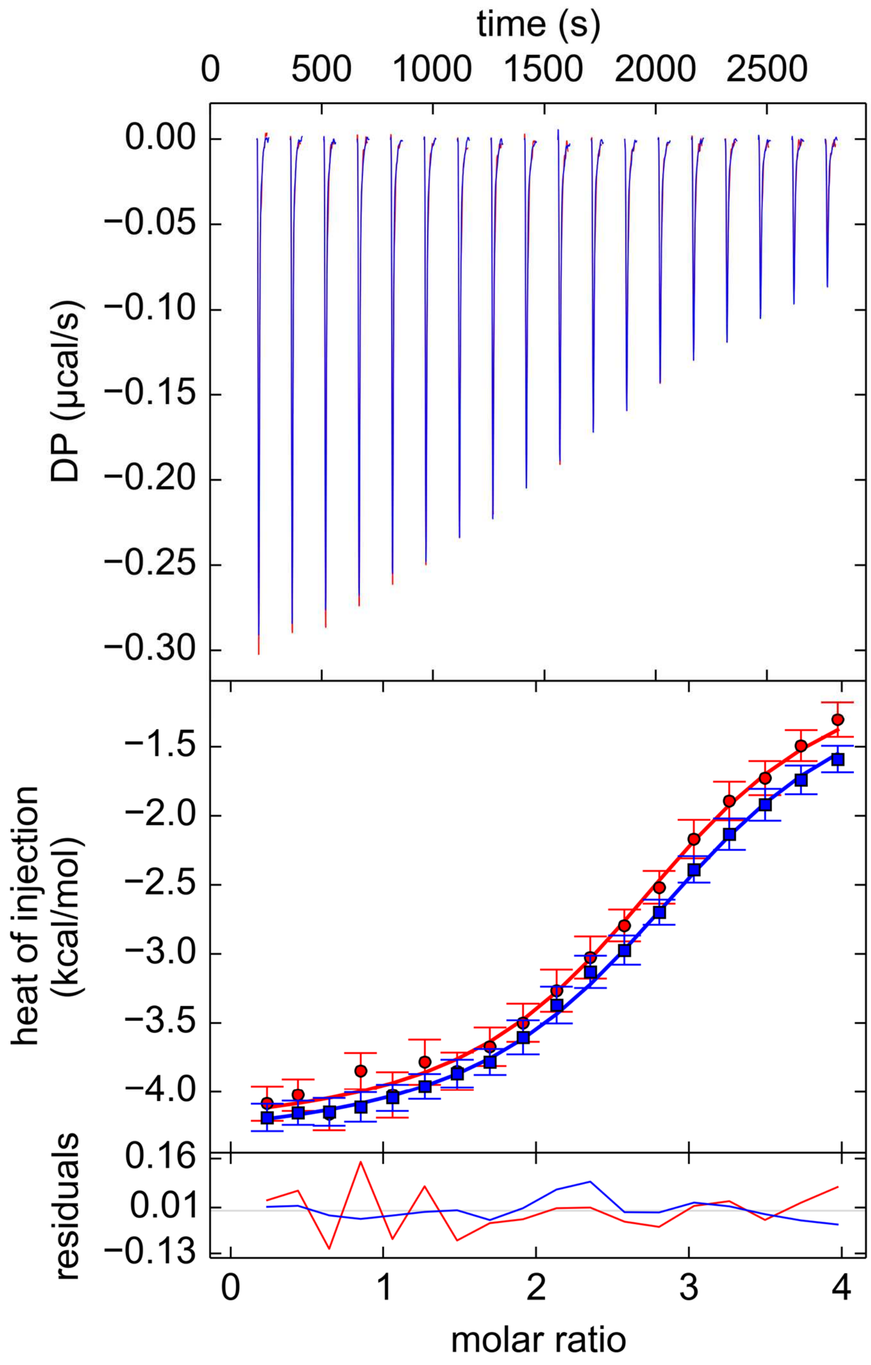

Figure S11. ITC Thermogram and nonlinear regression best fit for the binding of **3** to telo23.

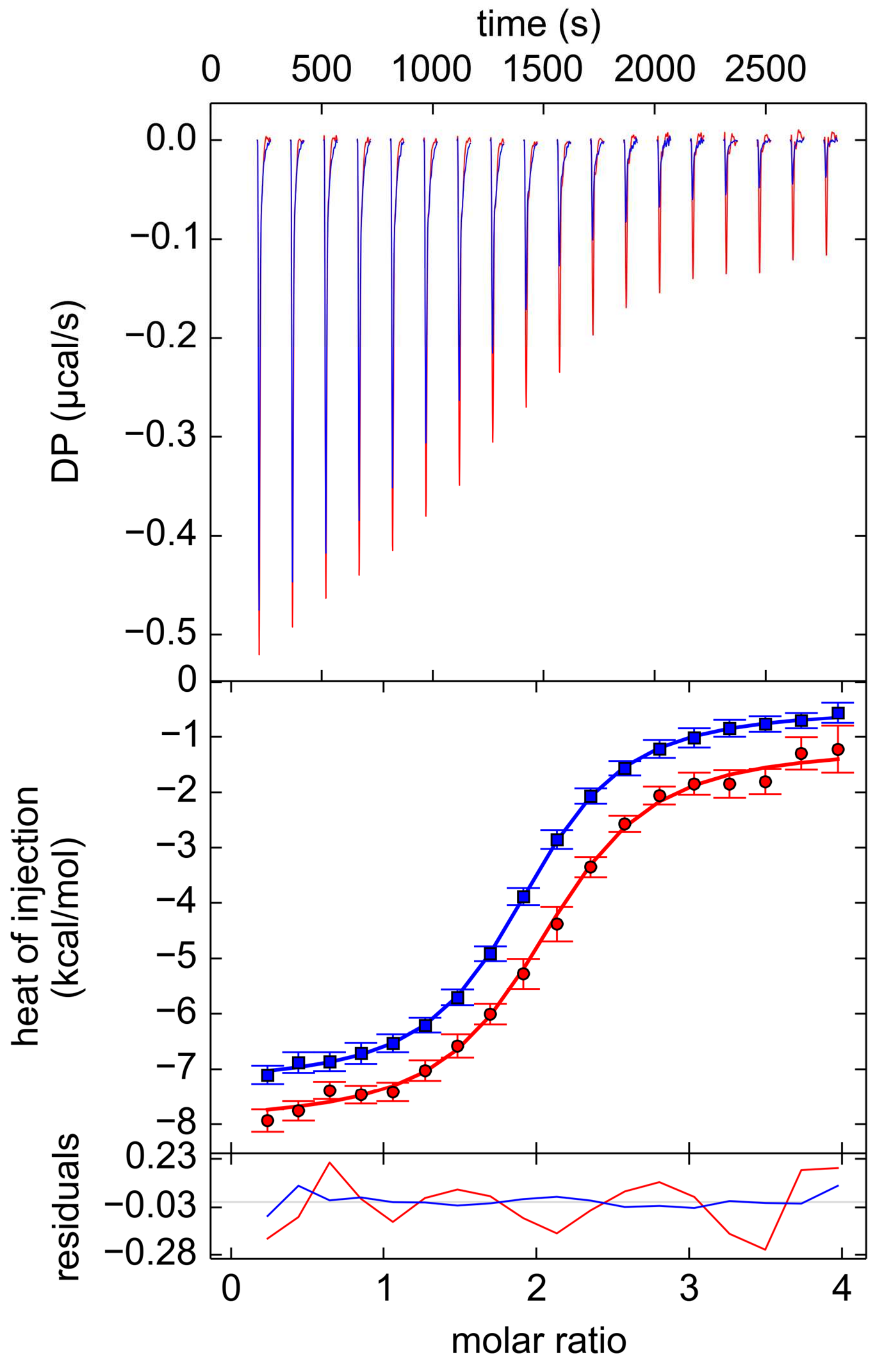

Figure S12. ITC Thermogram and nonlinear regression best fit for the binding of **5** to telo23.

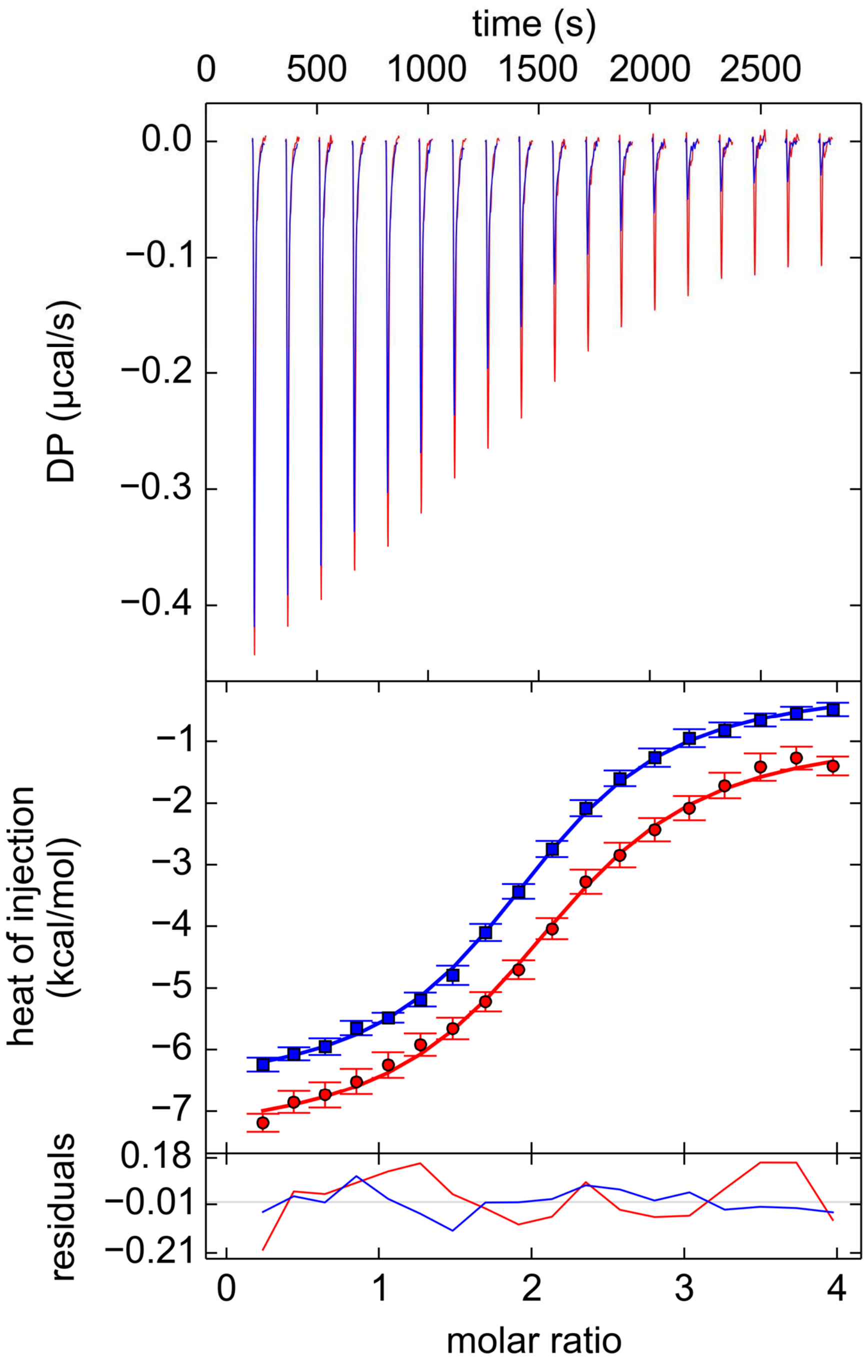

Figure S13. ITC Thermogram and nonlinear regression best fit for the binding of **6** to telo23.

Molecular Docking Studies

Molecular docking studies were performed using Molsoft ICM-Pro (Molsoft L.L.C., San Diago, CA)[8] The crystal structure of the tetrasubstituted naphthalene diimide ligand MM41 bound to a human telomeric DNA G-quadruplex 21-mer (PDB 4DA3)[9] was used as an initial modelling template to probe the binding modes of compounds **2-7** to the parallel K^+ human telomeric G-quadruplex. Subsequent DNA structures that were modelled include: the anti-parallel Na^+ human telomeric G-quadruplex with a ligand bound to the top face (2MCO)[10], the anti-parallel Na^+ human telomeric G-quadruplex with a ligand bound to the bottom face (2MCC)[10], the 3+1 hybrid human telomeric G-quadruplex (2MB3)[11] and the Pu27T parallel c-Myc promoter G-quadruplex (2MGN)[12]. Before docking, the G-quadruplex structure was converted to an ICM object, and the original ligand was removed. All atom types, hydrogen and missing heavy atoms were added to the receptor structure. According to the ICM local energy minimization method, the molecular conformation was represented by the internal coordinate variables. Energy calculations were based on a modified version of the ECEPP/3 force field with a distance-dependent dielectric function. Docking was conducted by a biased probability Monte Carlo (BPMC) minimization procedure. During the docking stimulation, grid energy, continuum electrostatic and entropy terms were used to evaluate the binding energy of ligands **1-7** with the DNA structure and the internal conformational energy of the ligand in order to find the most favourable orientation. Each independent docking was repeated 3 times, and the binding energies were computed.

Table S7. Predicted ICM-VLS scores for ligands X-Y. The lower the VLS score, the stronger the interaction is predicted to be. This score does not take into account ligand strain, which is reported below.

Compound	Parallel hTel G_4 (4DA3)	Antiparallel hTel G_4, top face (2MCO)	Antiparallel hTel G_4, bottom face (2MCC)	3+1 Hybrid htel G_4 (2MB3)	Pu24T, parallel c-Myc promoter G_4 (2MGN)
**1**	-13.61	-	-	-	-
**2**	-29.22	-	-	-21.59	-26.59
**3**	-15.92	-	-	-30.55	-39.22
**4**	-24.11	-	-	-28.31	-28.77
**5**	-26.16	-	-	-23.33	-31.34
**6**	-23.60	-31.82	-26.44	-17.02	-29.88
**7**	-17.61	-	-	-22.11	-

PDB IDs for the structures used are reported in brackets.

Table S8. Predicted ICM-Ligand Strain scores for ligands X-Y. The lower the Ligand Strain score, the less strained the ligand.

Compound	Parallel hTel G_4 (4DA3)	Antiparallel hTel G_4, top face (2MCO)	Antiparallel hTel G_4, bottom face (2MCC)	3+1 Hybrid htel G_4 (2MB3)	Pu24T, parallel c-Myc promoter G_4 (2MGN)
**1**	8.25	-	-	-	-
**2**	12.4	-	-	9.56	18.33
**3**	11.67	-	-	12.77	9.74
**4**	5.24	-	-	7.45	5.20
**5**	5.49	-	-	4.87	5.43
**6**	6.63	7.91	11.71	6.03	6.36
**7**	4.97	-	-	4.08	-

PDB IDs for the structures used are reported in brackets.

Table S 9. Predicted ICM-Scores for ligands X-Y. This score is an overall score, which takes into account both the interaction energy (VLS-Score) and the ligand strain (Ligand Strain Score). The lower the overall score, the better the predicted interaction overall.

Compound	Parallel hTel G_4 (4DA3)	Antiparallel hTel G_4, top face (2MCO)	Antiparallel hTel G_4, bottom face (2MCC)	3+1 Hybrid htel G_4 (2MB3)	Pu24T, parallel c-Myc promoter G_4 (2MGN)
**1**	-5.37	-	-	-	-
**2**	-16.82	-	-	-12.03	-8.26
**3**	-4.25	-	-	-17.78	-29.48
**4**	-18.87	-	-	-20.87	-23.57
**5**	-20.66	-	-	-18.47	-25.9
**6**	-16.97	-23.90	-14.72	-10.99	-23.53
**7**	-12.64	-	-	-18.03	-

PDB IDs for the structures used are reported in brackets.

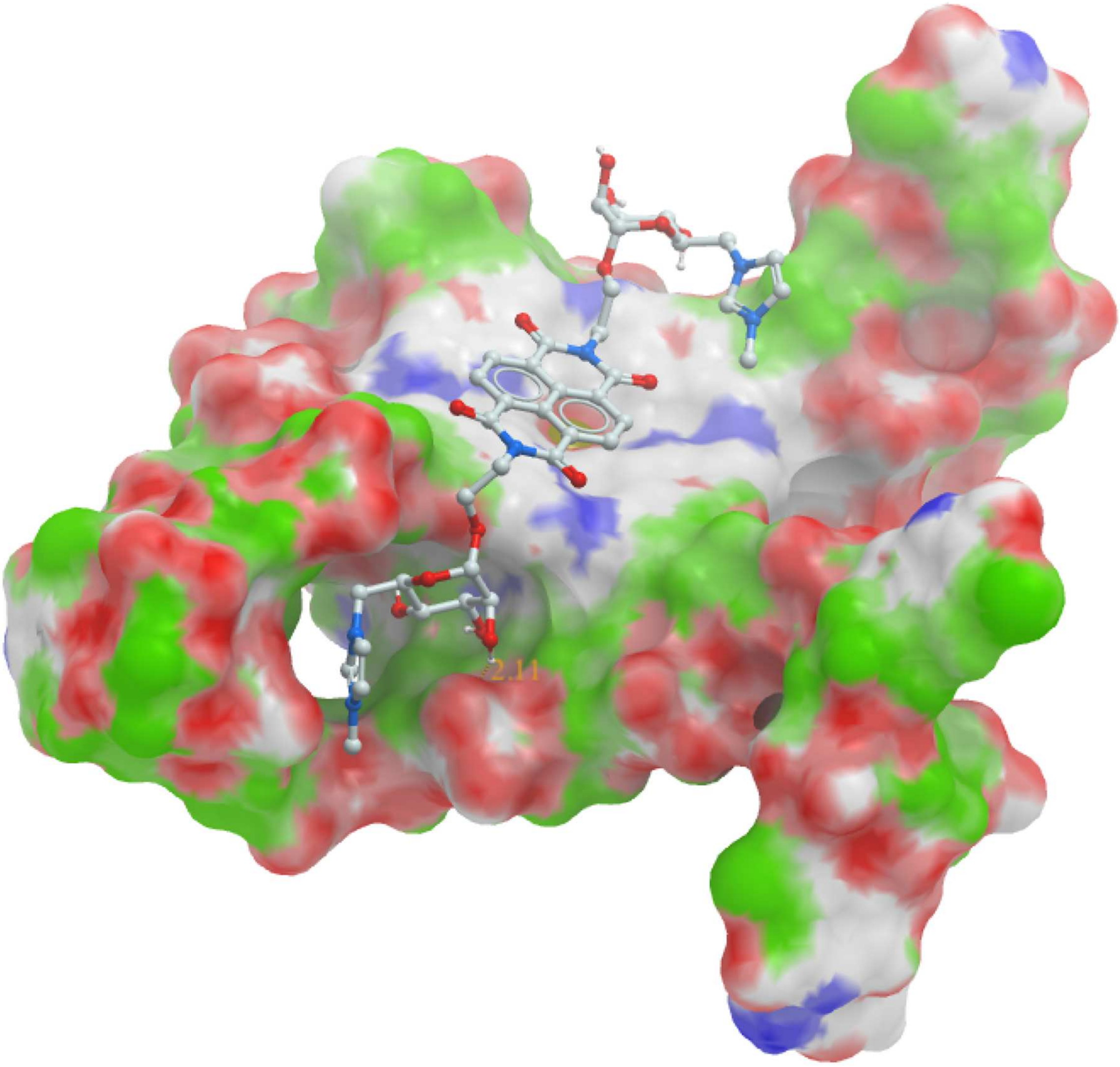

Figure S14. Modelled structure of compound **3** bound to the parallel K^+ human telomeric G-quadruplex. Note the hydrogen bonding interaction of the C-2 hydroxyl with the phosphate in the groove (2.11Å distance) and the electrostatic interaction of the imidazolium group with another phosphate in the groove of the quadruplex.

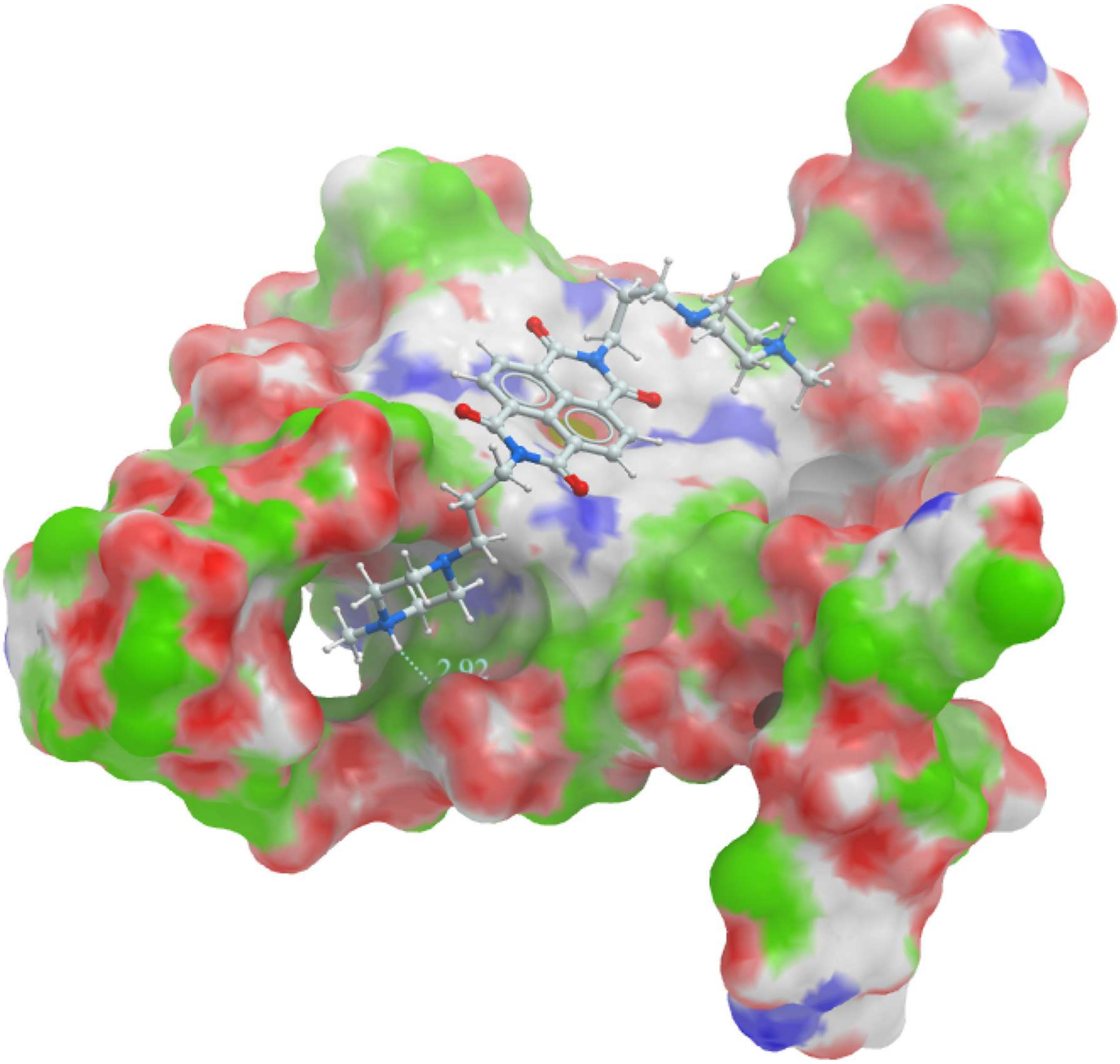

Figure S15. Modelled structure of compound **6** bound to the parallel K^+ human telomeric G-quadruplex. Note the hydrogen bonding interaction of the terminal amine with the phosphate in the groove of the quadruplex (2.92Å distance).

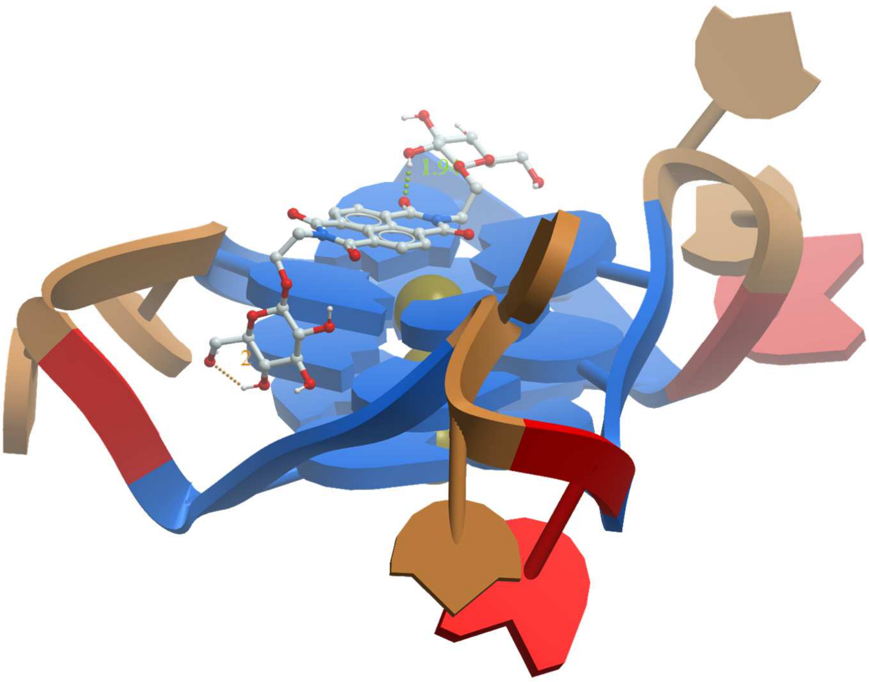

Figure S16. Side view of compound **1** docked into the parallel K^+ human telomeric G-quadruplex. Note the two intramolecular hydrogen bonding interactions on the ligand between C-4 and C-6 hydroxyls (2.00Å distance) on one side and C-2 and an NDI C=O on the other side (1.94Å distance).

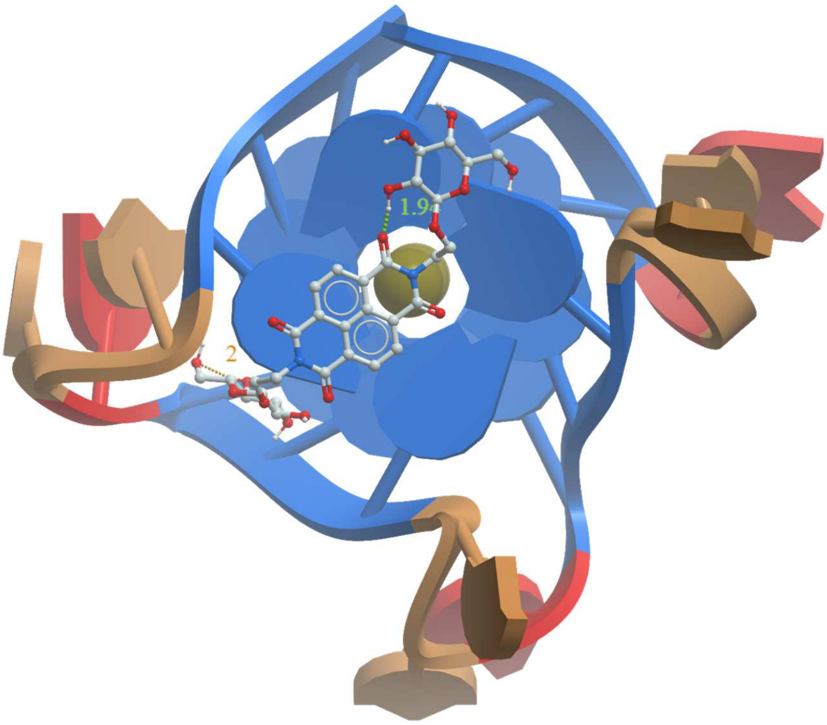

Figure S17. Top view of compound **1** docked into the parallel K^+ human telomeric G-quadruplex. Note the two intramolecular hydrogen bonding interactions on the ligand between C-4 and C-6 hydroxyls (2.00Å distance) on one side and C-2 and an NDI C=O on the other side (1.94Å distance).

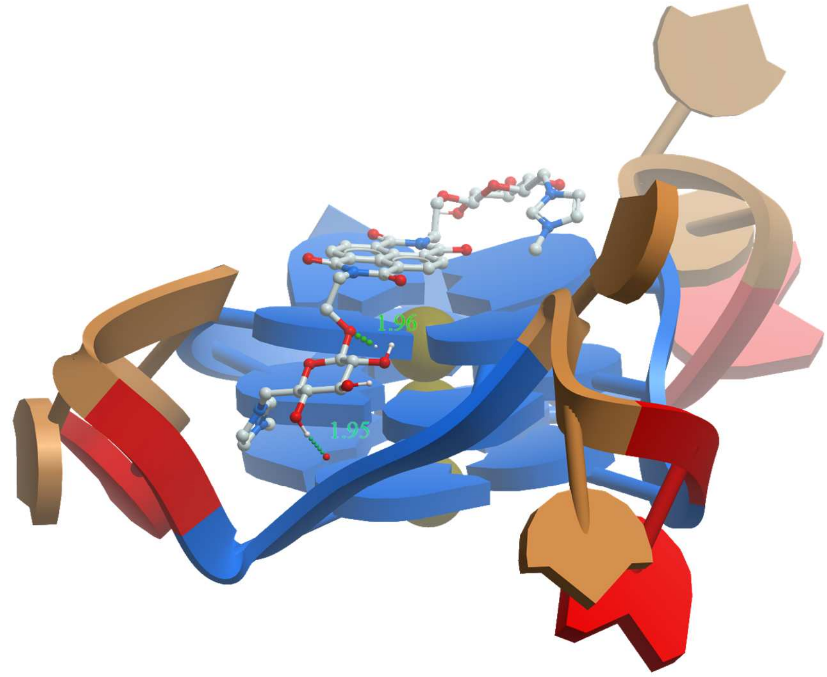

Figure S18. Side view of compound **2** docked into the parallel K^+ human telomeric G-quadruplex. Note the two hydrogen bonding interactions at C-1 (1.96Å distance) and C-4 (1.95Å distance).

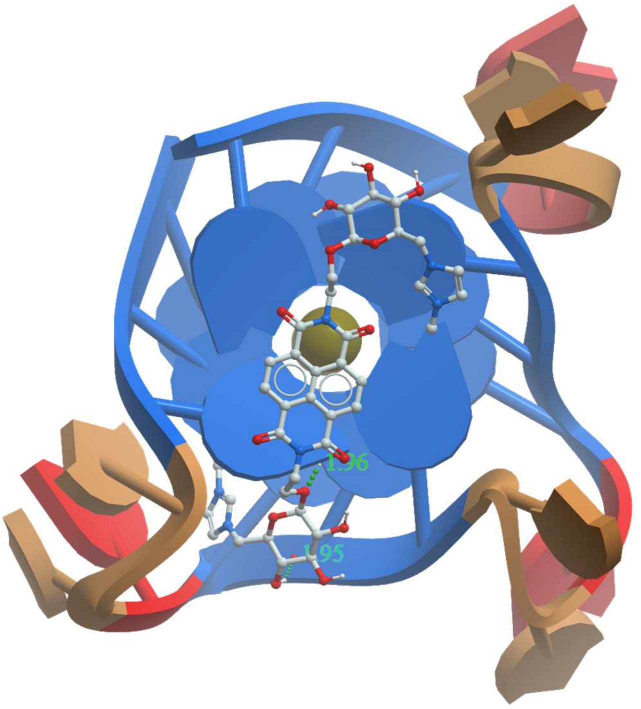

Figure S19. Top view of compound **2** docked into the parallel K^+ human telomeric G-quadruplex. Note the two hydrogen bonding interactions at C-1 (1.96Å distance) and C-4 (1.95Å distance).

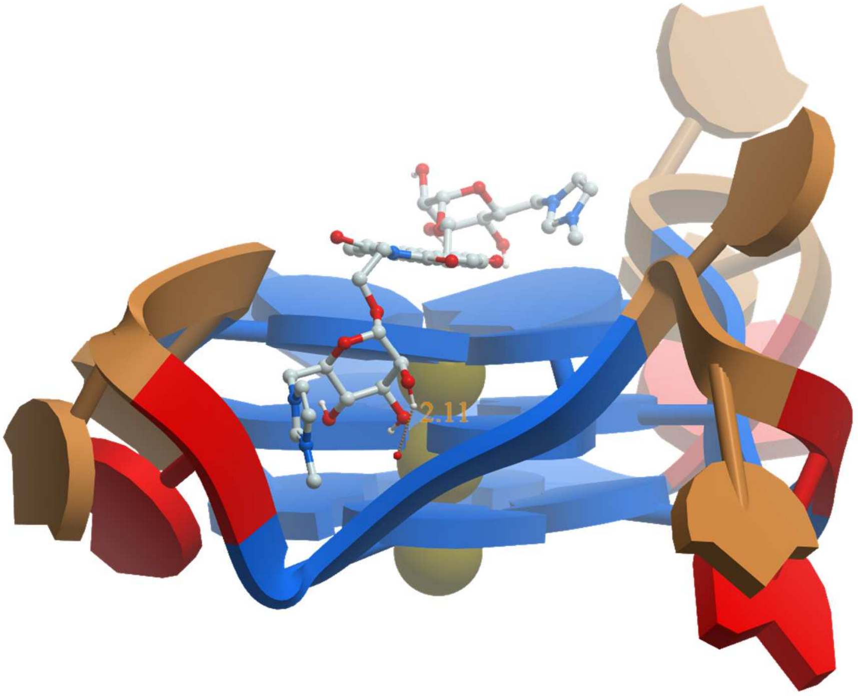

Figure S20. Side view of compound **3** docked into the parallel K^+ human telomeric G-quadruplex. Note the hydrogen bonding interaction at C-2 (2.11Å distance).

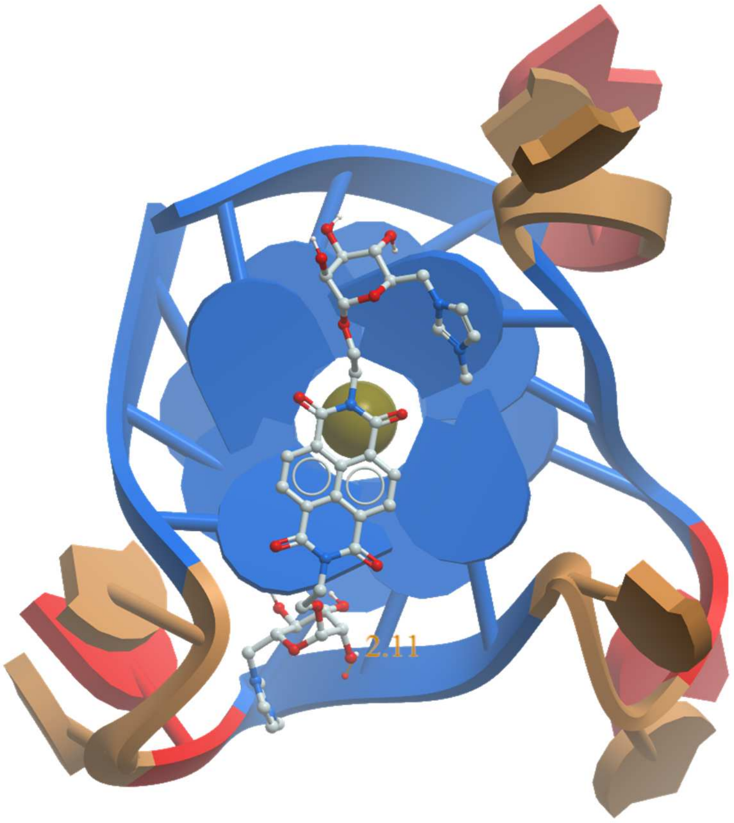

Figure S21. Top view of compound **3** docked into the parallel K^+ human telomeric G-quadruplex. Note the hydrogen bonding interaction at C-2 (2.11Å distance).

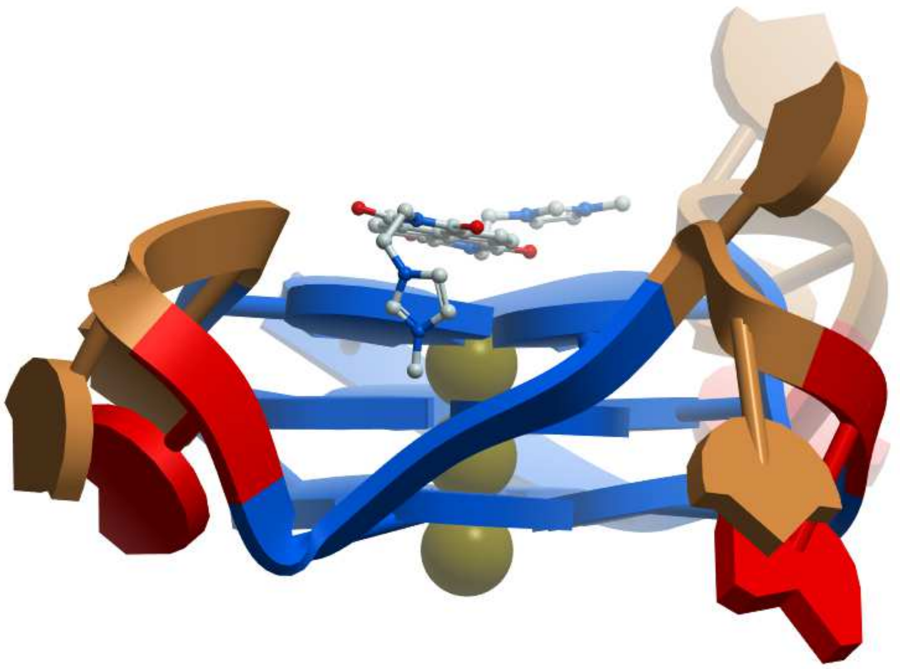

Figure S22. Side view of compound **4** docked into the parallel K^+ human telomeric G-quadruplex.

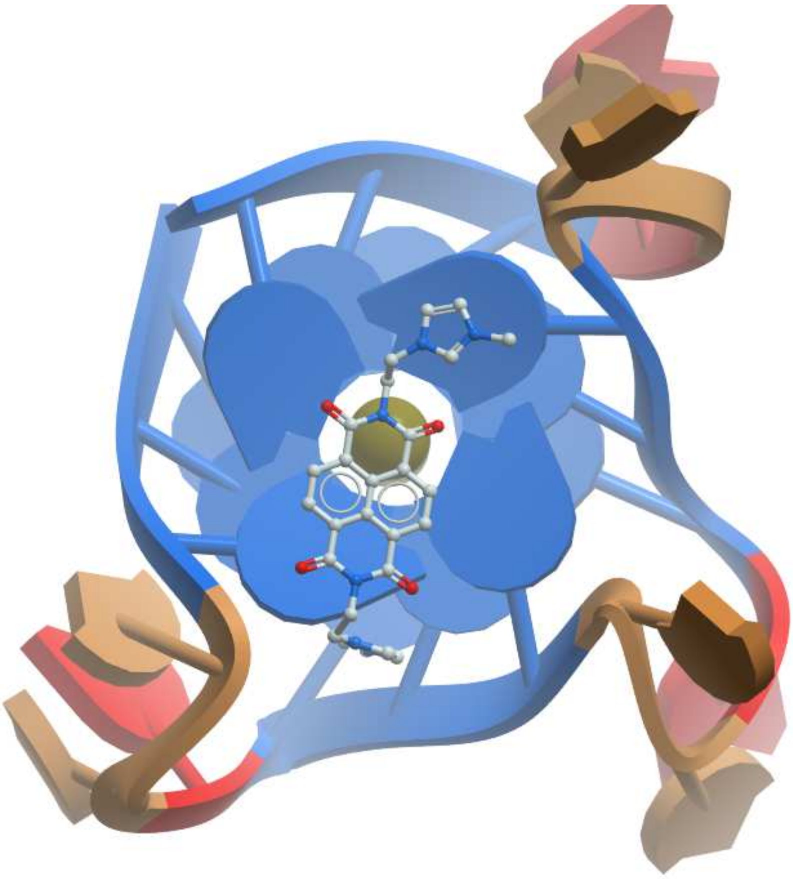

Figure S23. Top view of compound **4** docked into the parallel K^+ human telomeric G-quadruplex.

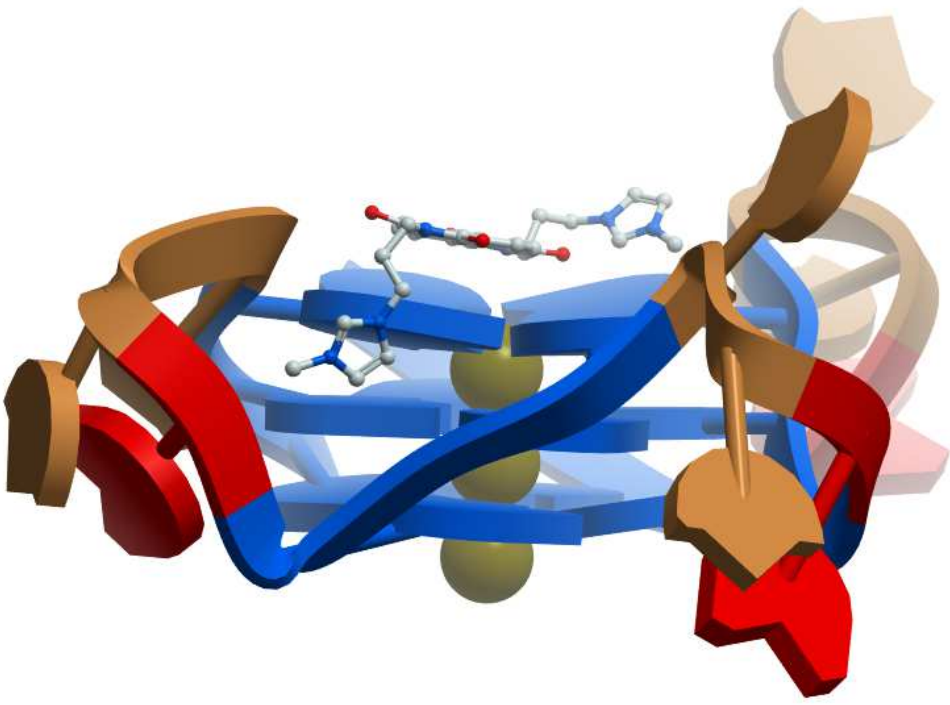

Figure S24. Side view of compound **5** docked into the parallel K^+ human telomeric G-quadruplex.

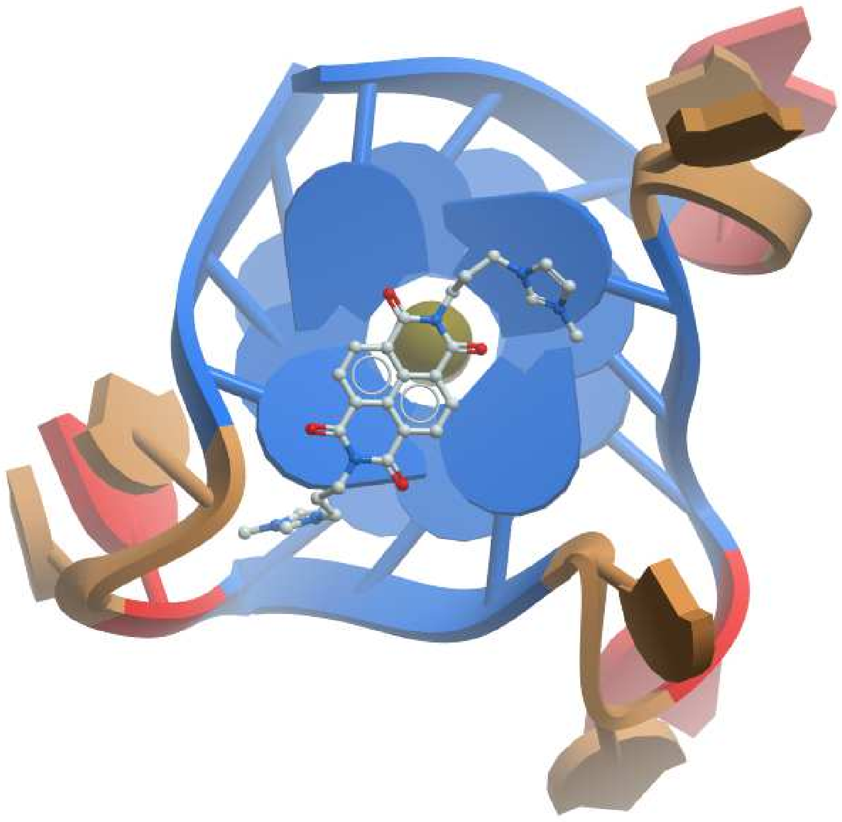

Figure S25. Top view of compound **5** docked into the parallel K^+ human telomeric G-quadruplex.

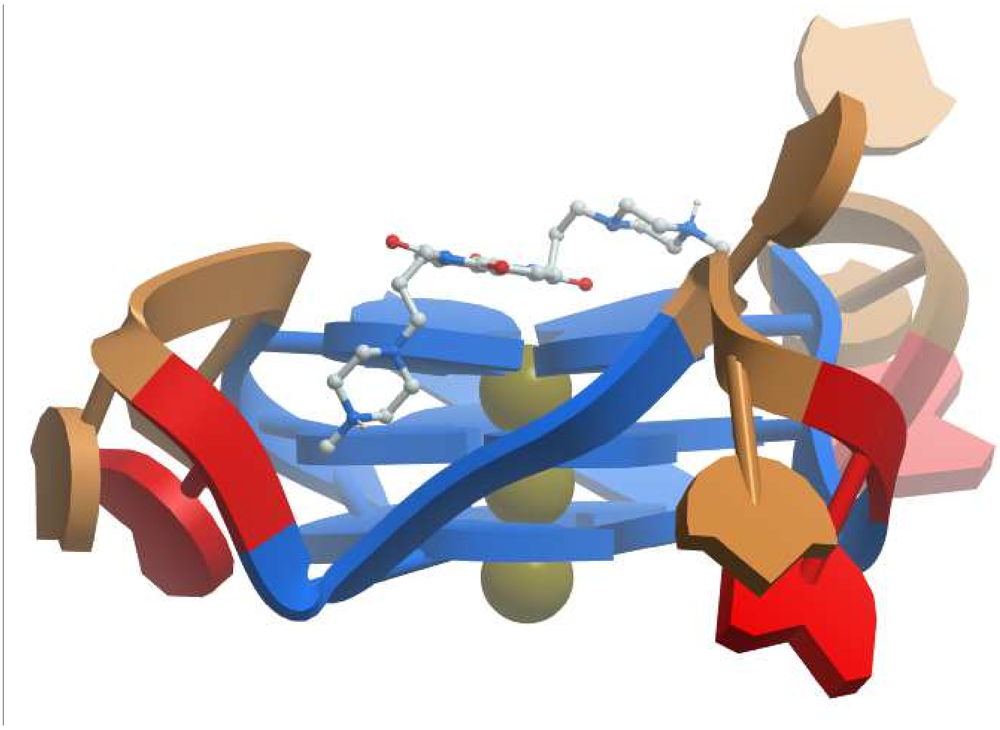

Figure S26. Side view of compound **6** docked into the parallel K^+ human telomeric G-quadruplex.

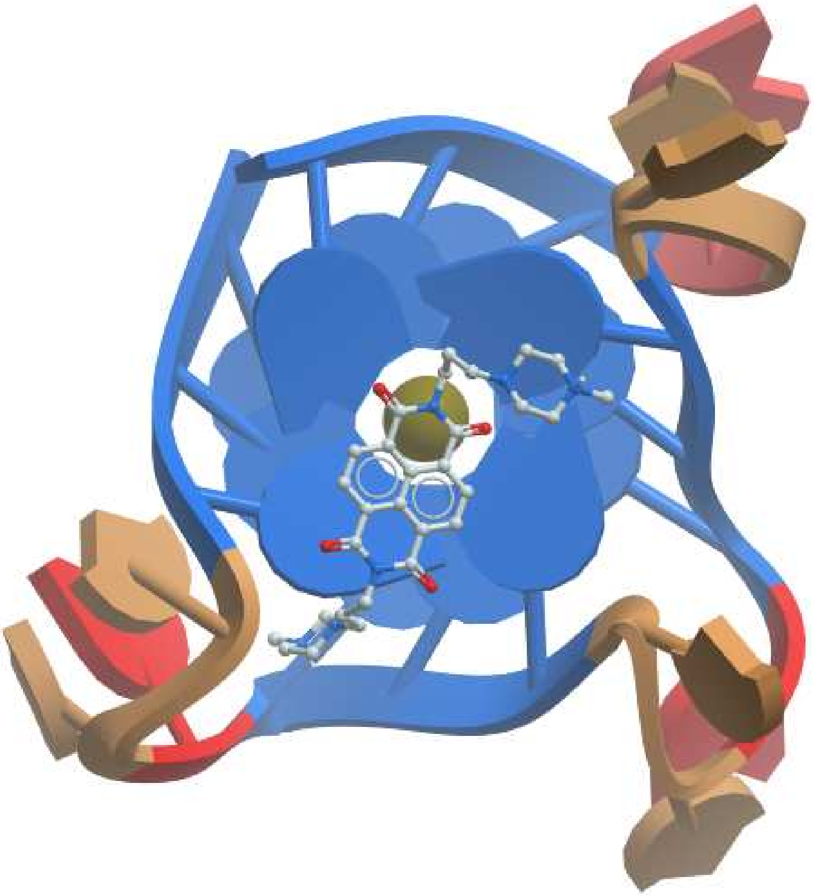

Figure S27. Top view of compound **6** docked into the parallel K^+ human telomeric G-quadruplex.

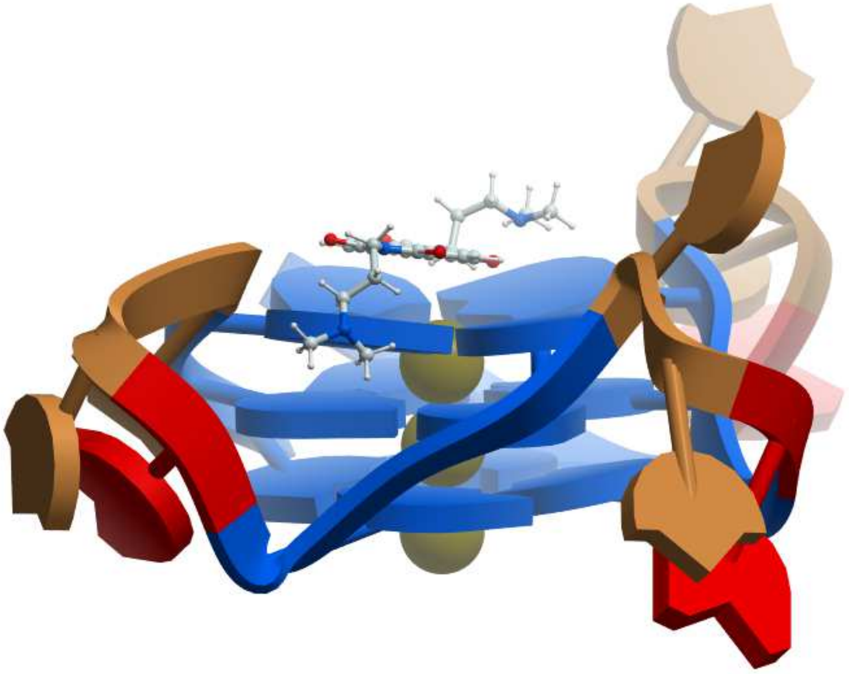

Figure S28. Side view of compound **7** docked into the parallel K^+ human telomeric G-quadruplex.

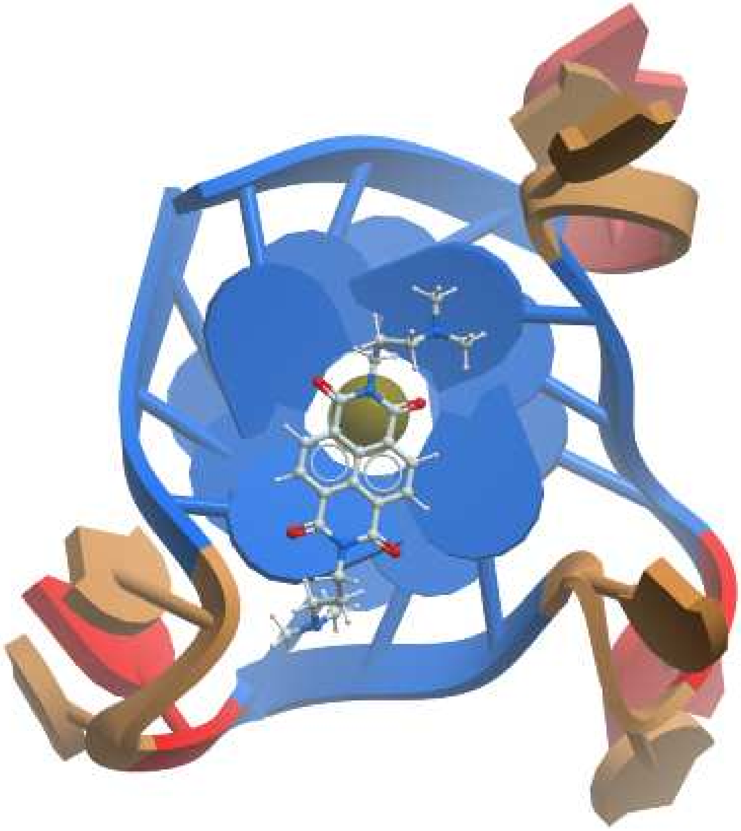

Figure S29. Top view of compound **7** docked into the parallel K^+ human telomeric G-quadruplex.

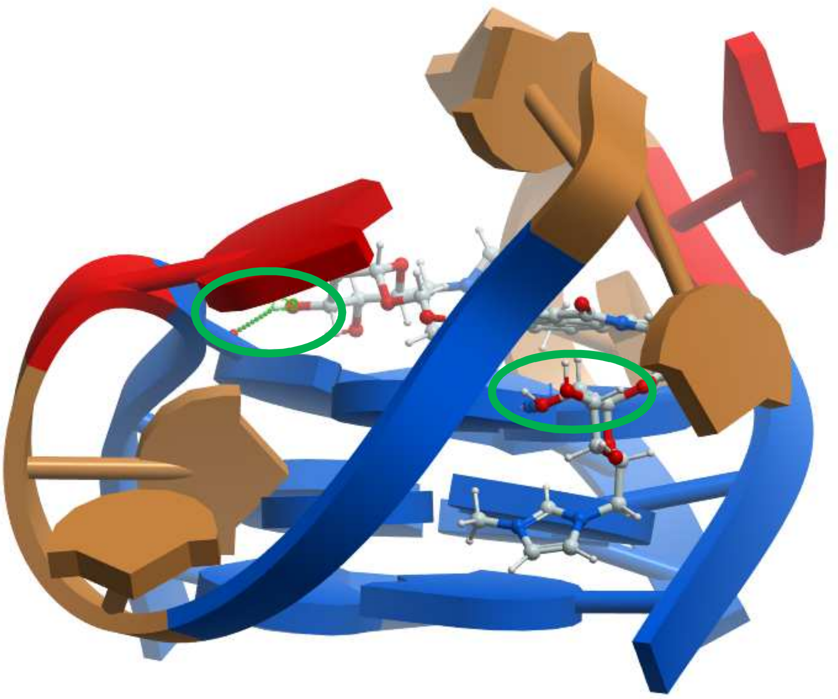

Figure S30. Side view of compound **3** docked into the 3+1 hybrid K^+ human telomeric G-quadruplex. Note the hydrogen bonding interactions between the C-2 hydroxyl and a guanine NH_2 on one side (1.94Å distance) and C-3 hydroxyl and a ribose oxygen on the other side (2.29Å distance) (circled).

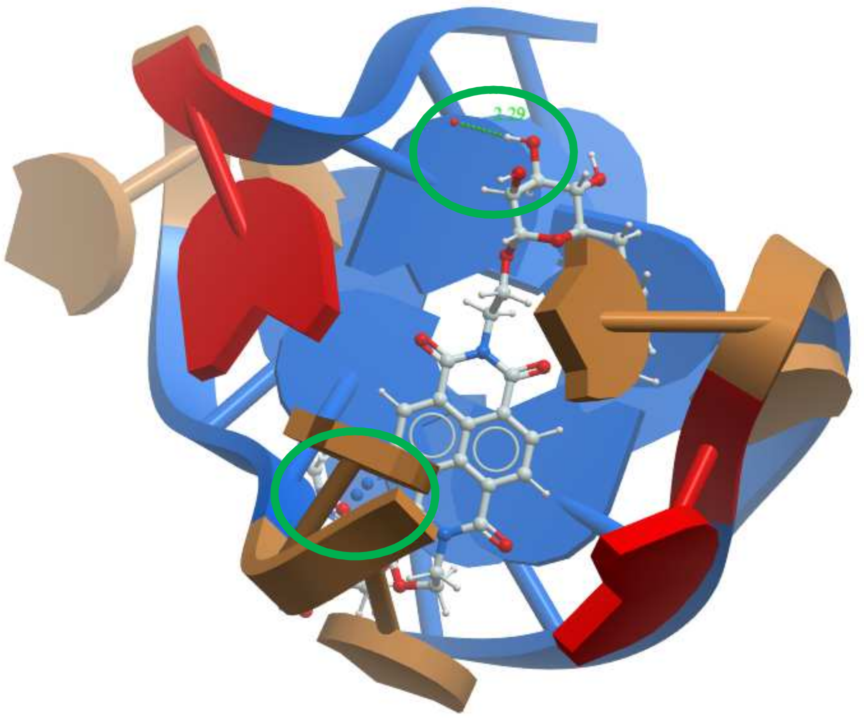

Figure S31. Top view of compound **3** docked into the 3+1 hybrid K^+ human telomeric G-quadruplex. Note the hydrogen bonding interactions between the C-2 hydroxyl and a guanine NH_2 on one side (1.94Å distance) and the C-3 hydroxyl and a ribose oxygen on the other side (2.29Å distance) (circled).

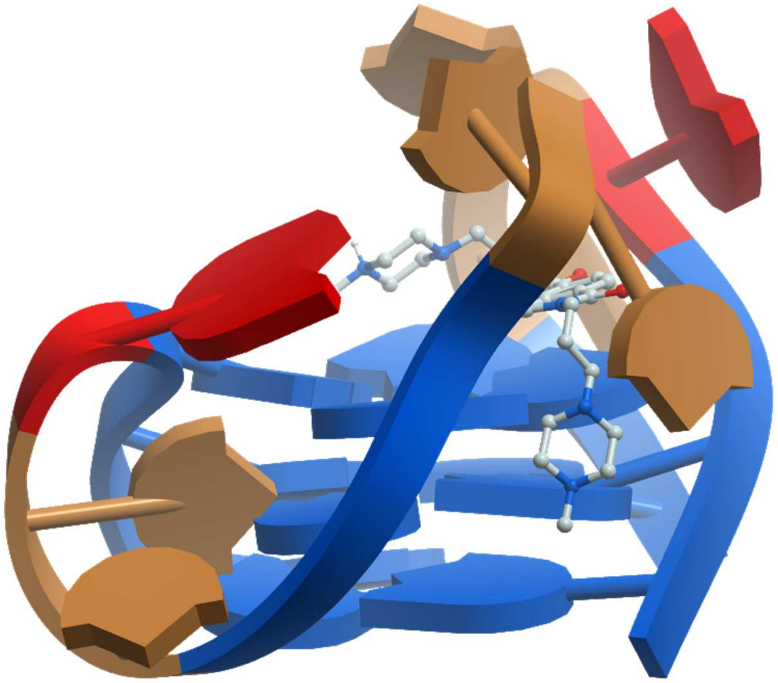

Figure S32. Side view of compound **6** docked into the 3+1 hybrid K^+ human telomeric G-quadruplex.

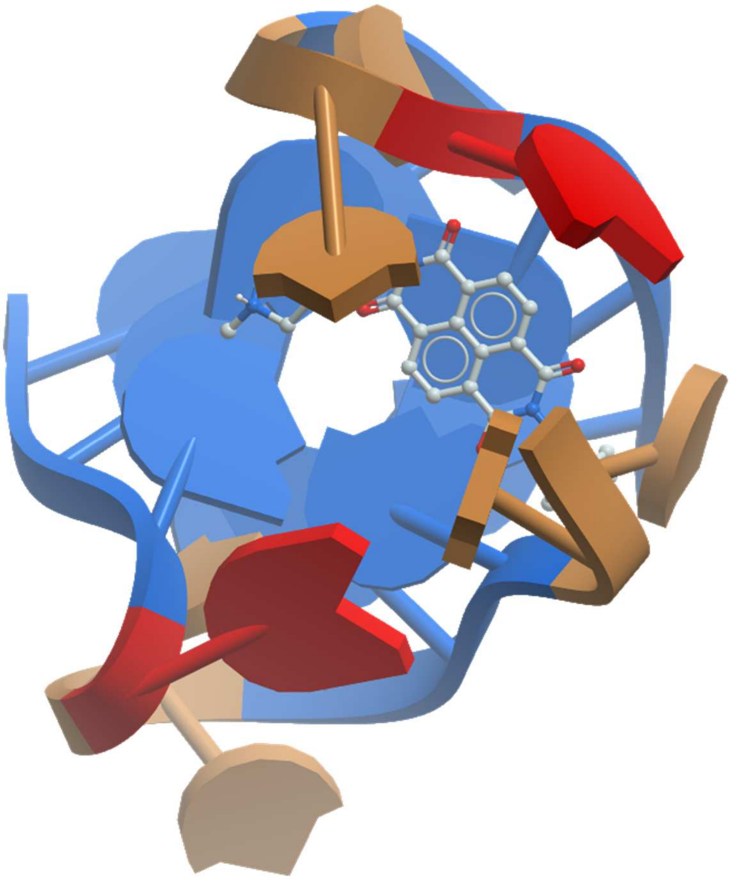

Figure S33. Top view of compound **6** docked into the 3+1 hybrid K^+ human telomeric G-quadruplex.

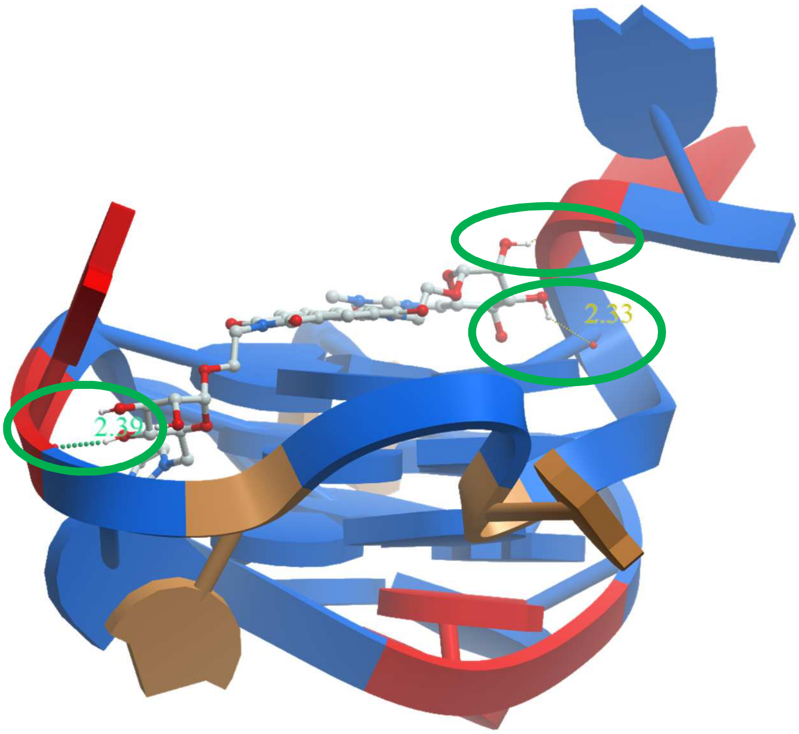

Figure S34. Side view of compound **3** docked into the Pu27T c-Myc parallel G-quadruplex. Note the hydrogen bonding interactions between the C-4 hydroxyl and a phosphate on one side (2.39Å distance), and the C-2 & C-3 hydroxyls and two phosphates on the other side (2.25Å & 2.33Å distance respectively) (circled).

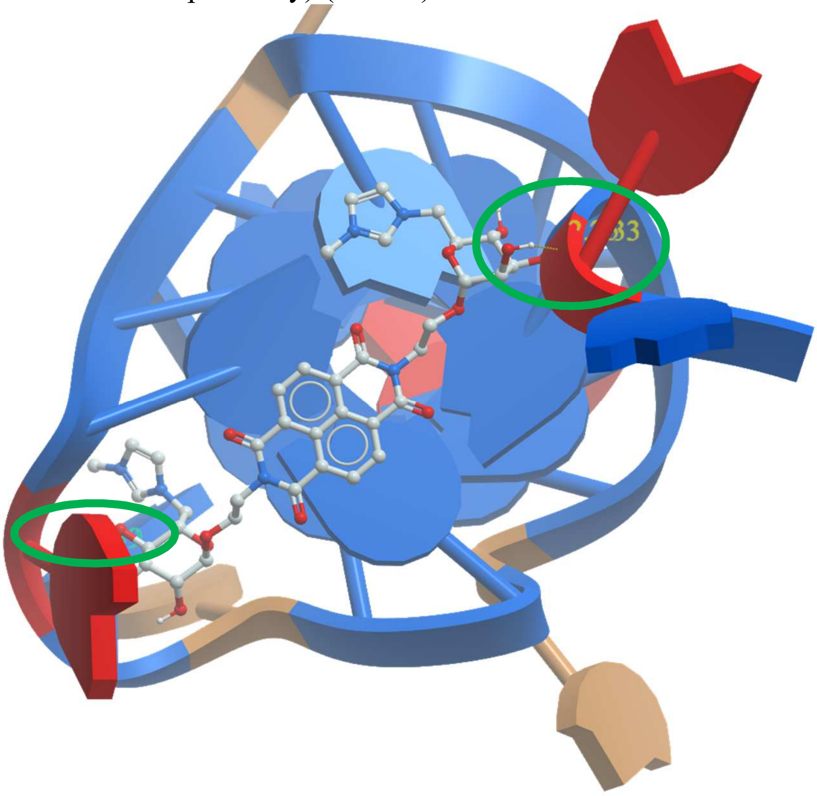

Figure S35. Top view of compound **3** docked into the Pu27T c-Myc parallel G-quadruplex. Note the hydrogen bonding interactions between the C-4 hydroxyl and a phosphate on one side (2.39Å distance), and the C-2 & C-3 hydroxyls and two phosphates on the other side (2.25Å & 2.33Å distance respectively) (circled).

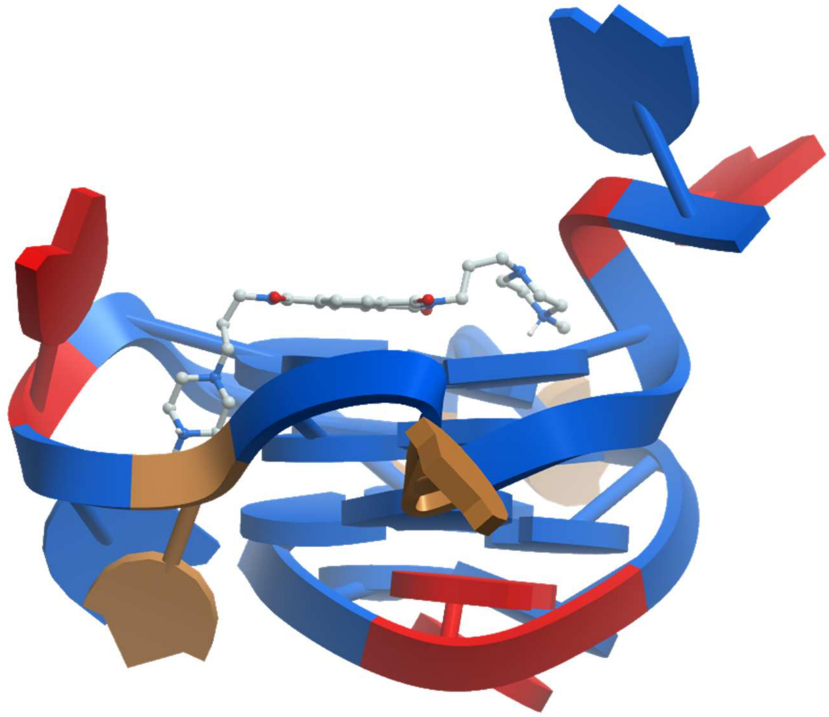

Figure S36. Side view of compound **6** docked into the Pu27T c-Myc parallel G-quadruplex.

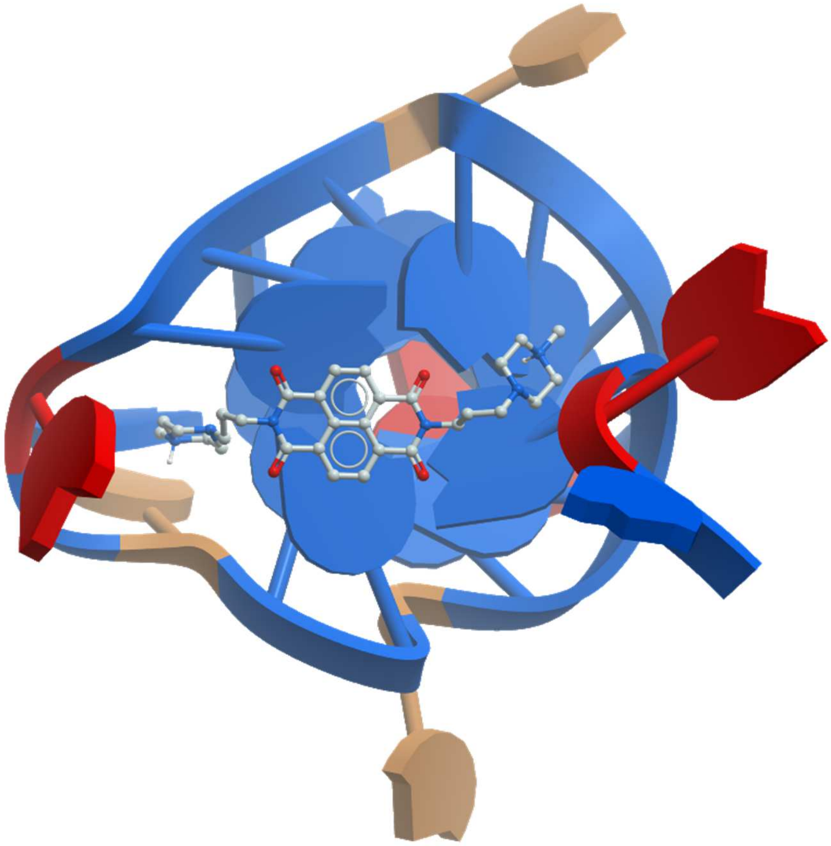

Figure S37. Top view of compound **6** docked into the Pu27T c-Myc parallel G-quadruplex.

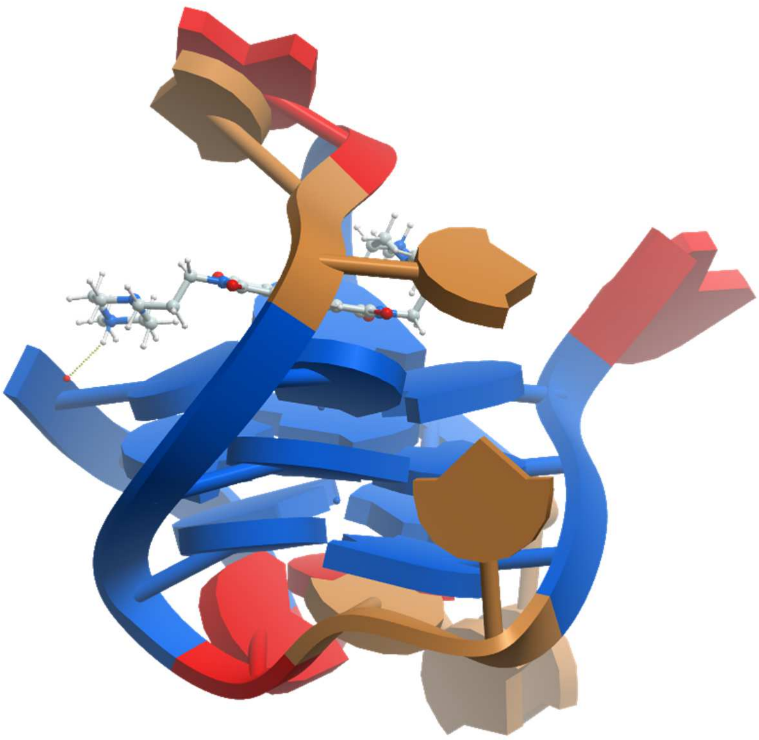

Figure S38. Side view of compound **6** docked into the top face of the antiparallel Na^+ human telomeric G-quadruplex. Note the hydrogen bonding interaction with a phosphate (2.24Å distance).

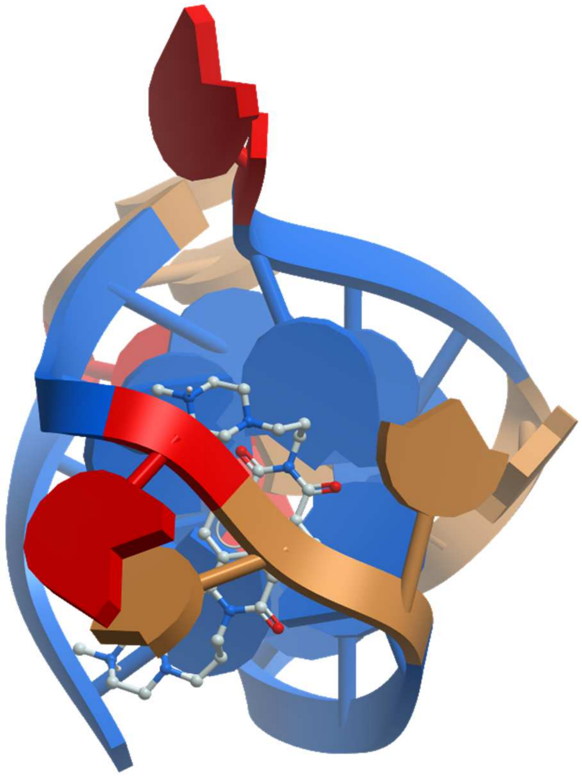

Figure S 39. Top view of compound **6** docked into the top face of the antiparallel Na^+ human telomeric G-quadruplex.

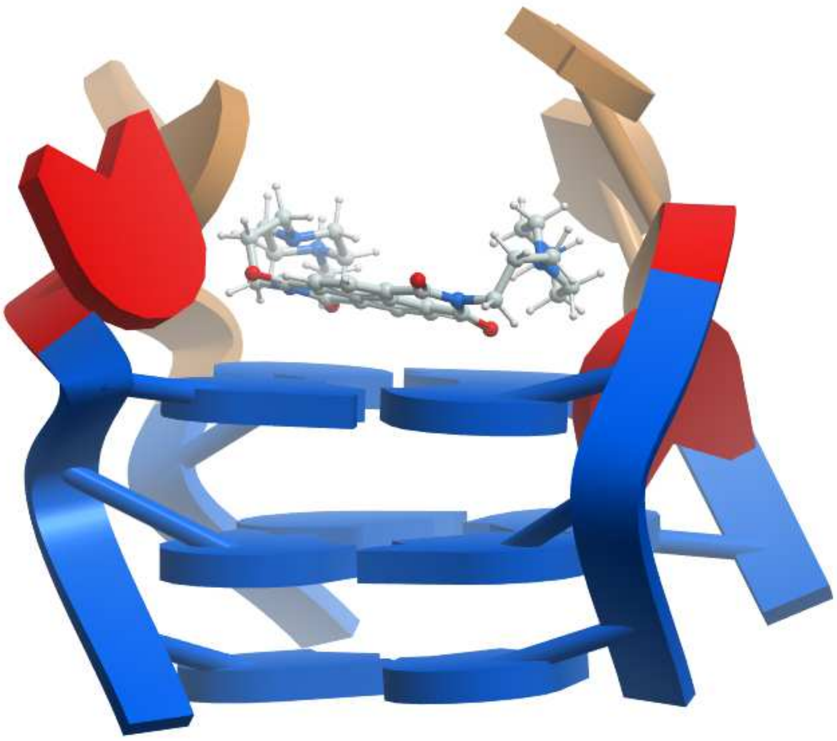

Figure S40. Side view of compound **6** docked into the top face of the antiparallel Na^+ human telomeric G-quadruplex.

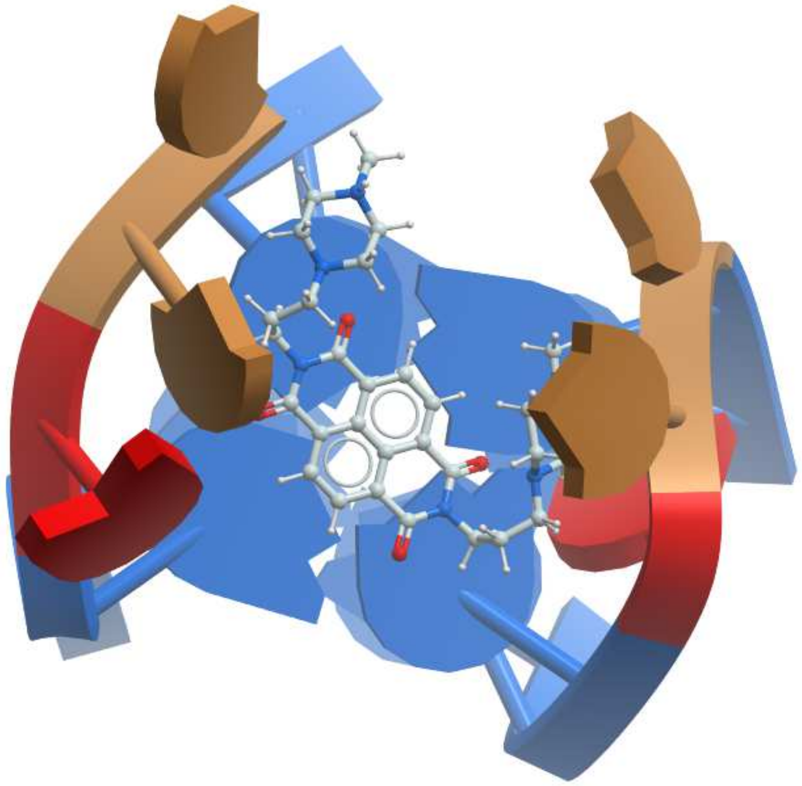

Figure S41. Top view of compound **6** docked into the top face of the antiparallel Na^+ human telomeric G-quadruplex.

Cell Culture Protocols

WI-38 (Caucasian fibroblast-like fetal lung cells) and MCF7 (Human breast adenocarcinoma cell line) were grown in Minimal Essential Medium (MEM), HeLa (Human cervical carcinoma cell line) and MDA (Human breast adenocarcinoma) were grown in Dulbecco's Minimal Essential Medium (DMEM). All growth media were supplemented with antibiotic-antimycotic (Anti-Anti) and 10-20 % fetal bovine serum (FBS). Confluent cultures were detached from the surface using trypsin (Tryp LE Express) and plated at $2*10^4$ cells/well in 96-well plates. Cell culture media and additives were purchased from Invitrogen, Life Technologies.

Toxicity Assays

The influence of compounds **1 - 7** and Doxorubicin on cell survival after exposure to the compounds was quantified by measuring calcein fluorescence. The fluorescence, retained within live cells only, results from activity of esterases on the (nonfluorescent) calcein AM (Molecular Probes). Changes in cell metabolism were assessed using AlamarBlue (AB, Life Technologies), a cytosolic substrate for reductive metabolism (resazurin to resorufin) whose fluorescence spectrum changes on reduction by cytosolic enzymes. WI-38 (fetal lung cells), HeLa (human cervical carcinoma) MDA (human breast adenocarcinoma) and MCF7 (human breast adenocarcinoma) were incubated with 0-100 μM of compounds **1 - 7** and Doxorubicin for 72 hours. Each experiment was repeated at least twice, in medium with reduced FBS (5%), with each data point conducted in octuplicate. After 72h the plates were washed with PBS, and AB (5 % solution), calcein (5 μM) and sytox (0.1 μM) were added in medium without FBS. After 1 hour incubation, the fluorescence of all three dyes was read using a plate reader (BMG Labtech CLARIOstar) (AB λex = 545 nm λem = 590 nm, calcein λex = 494 nm, λem = 517 nm, sytox red λex = 633 nm λem = 660 nm). Results are expressed as percentages of control, versus the logarithm of the ligand concentration. Curves were fitted to determine the absolute IC50s using the non-linear regression function of GraphPad Prism 6, using the 'log(inhibitor) vs. normalized response – variable slope' or 'log(inhibitor) vs response – variable slope' equations as appropriate (Table 3 and Table S10).

Table S10. Cytotoxicity of compounds 1-7 and doxorubicin determined by 72h Alamar Blue Assay (measure of reductive metabolism).[a]

Cell Line	WI-38	HeLa	MCF7	MDA
**Doxorubicin**	0.20<br>(0.17, 0.22)	0.40<br>(0.35,0.45)	0.79<br>(0.76,0.85)	1.30<br>(1.05,1.61)
**1**	>100	>100	>100	>100
**2**	>100	>100	>100	>100
**3**	~100	>100	>100	>100
**4**	22.9<br>(19.94,26.30)	0.30<br>(0.29,0.32)	12.97<br>(11.83,14.21)	42.63<br>(35.98,50.50)
**5**	19.03<br>(14.58,24.84)	0.15<br>(0.14,0.16)	3.35<br>(3.19,3.52)	55.22<br>(40.61,75.10)
**6**	1.02<br>(0.94,1.11)	0.50<br>(0.44,0.57)	3.00<br>(2.66,3.38)	0.45<br>(0.38,0.54)
**7**	0.99<br>(0.92,1.06)	0.34<br>(0.32,0.36)	0.71<br>(0.68,0.75)	0.18<br>(0.17,0.19)

a Absolute IC50 values measured in µM, with 95% confidence interval in brackets.

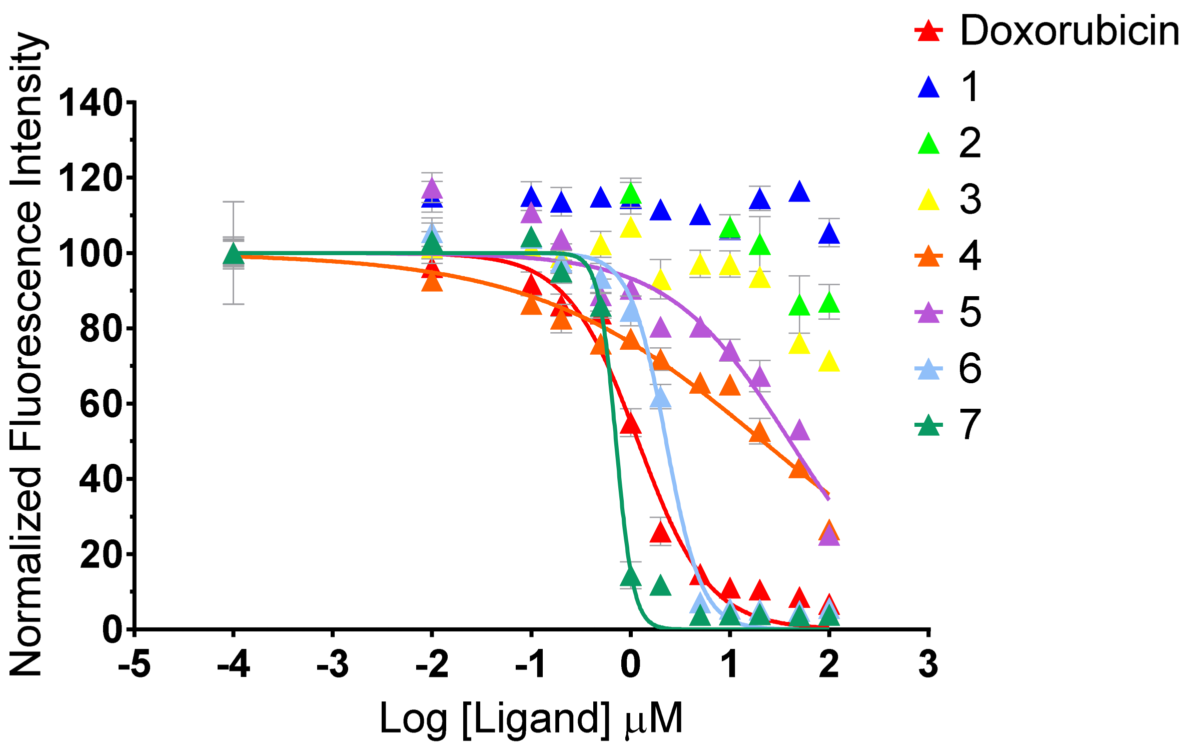

Figure S42. Calcein Assay data for WI-38 with doxorubicin and **1**-**7**. The very small (below assay sensitivity) number of cells surviving at high concentration of Doxorubicin, **6** and **7** invalidates metabolism/cell calculations.

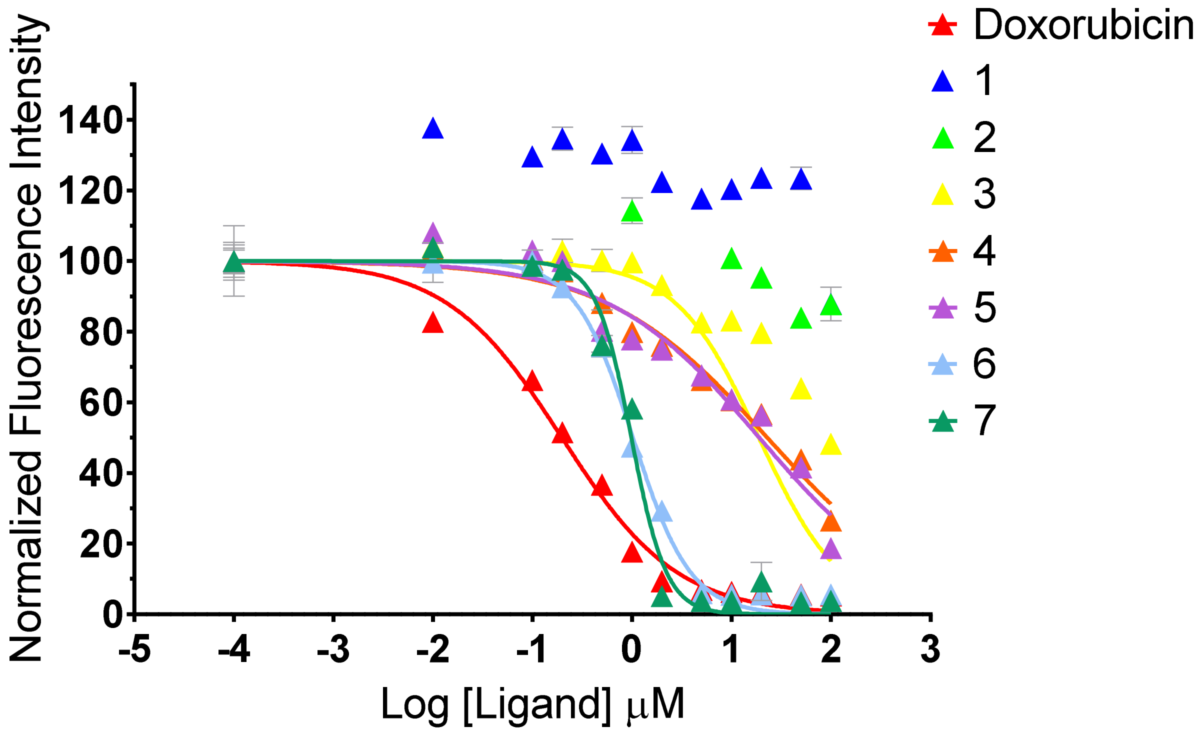

Figure S43. Effects on reductive metabolism (Alamar Blue Assay) for WI-38 after 72h incubation with doxorubicin and **1**-**7**.

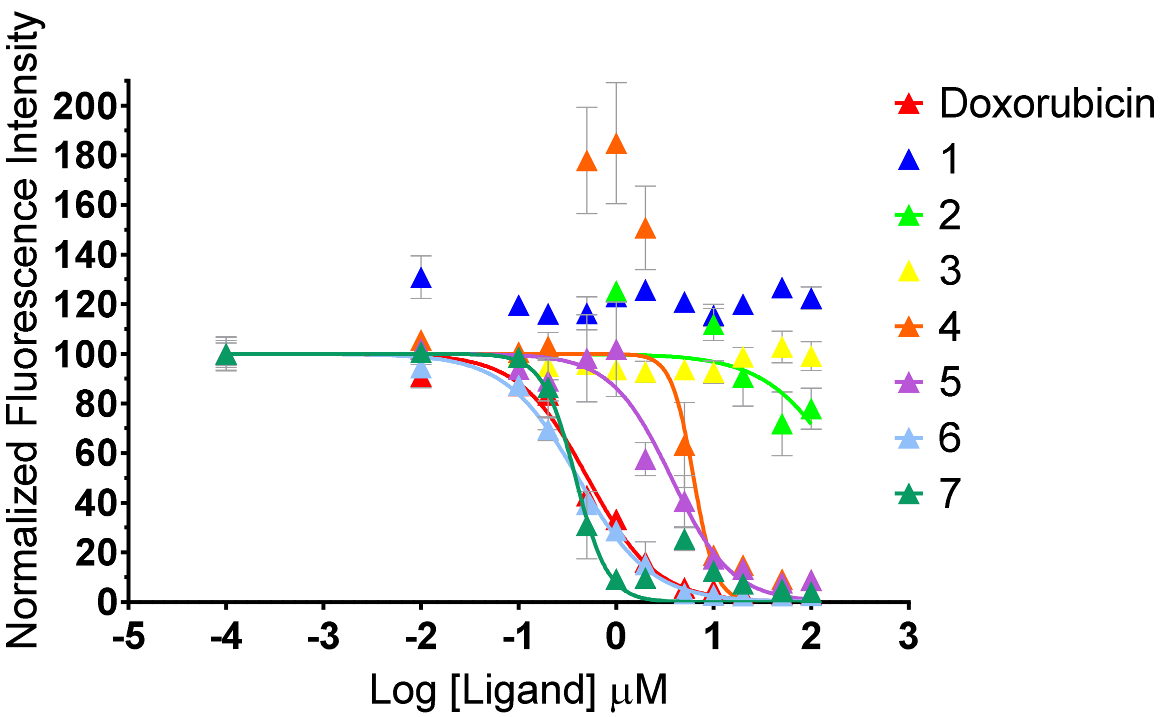

Figure S44. Calcein Assay data for HeLa with doxorubicin and **1**-**7**. The very small (below assay sensitivity) number of cells surviving at high concentration of Doxorubicin, **6** and **7** invalidates metabolism/cell calculations.

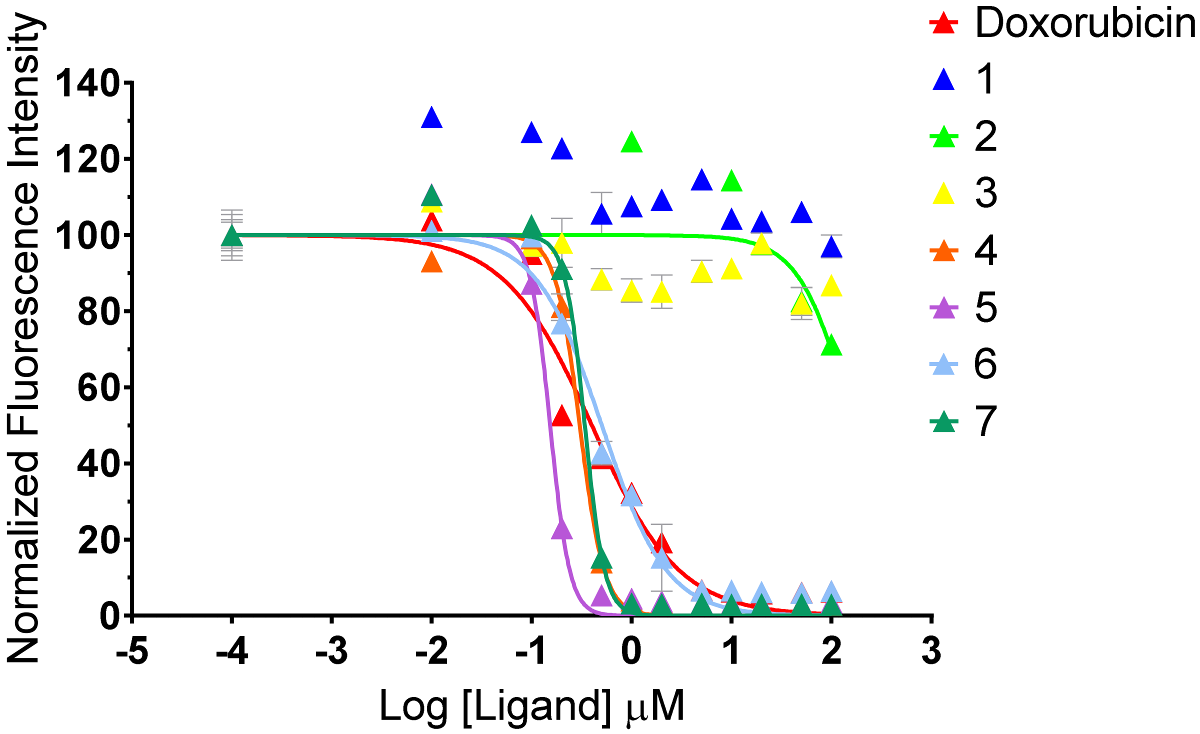

Figure S45. Effects on reductive metabolism (Alamar Blue Assay) for HeLa after 72h incubation with doxorubicin and **1**-**7**.

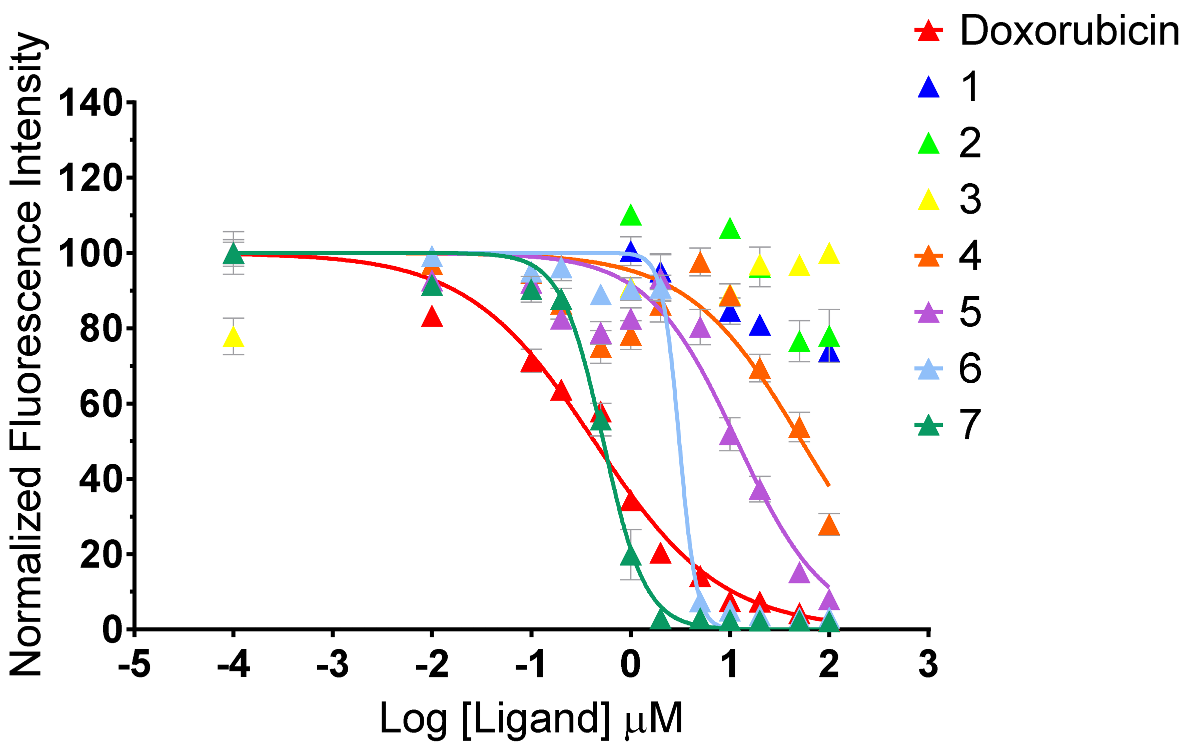

Figure S46. Calcein Assay data for MCF7 with doxorubicin and **1**-**7**. The very small (below assay sensitivity) number of cells surviving at high concentration of Doxorubicin, **6** and **7** invalidates metabolism/cell calculations.

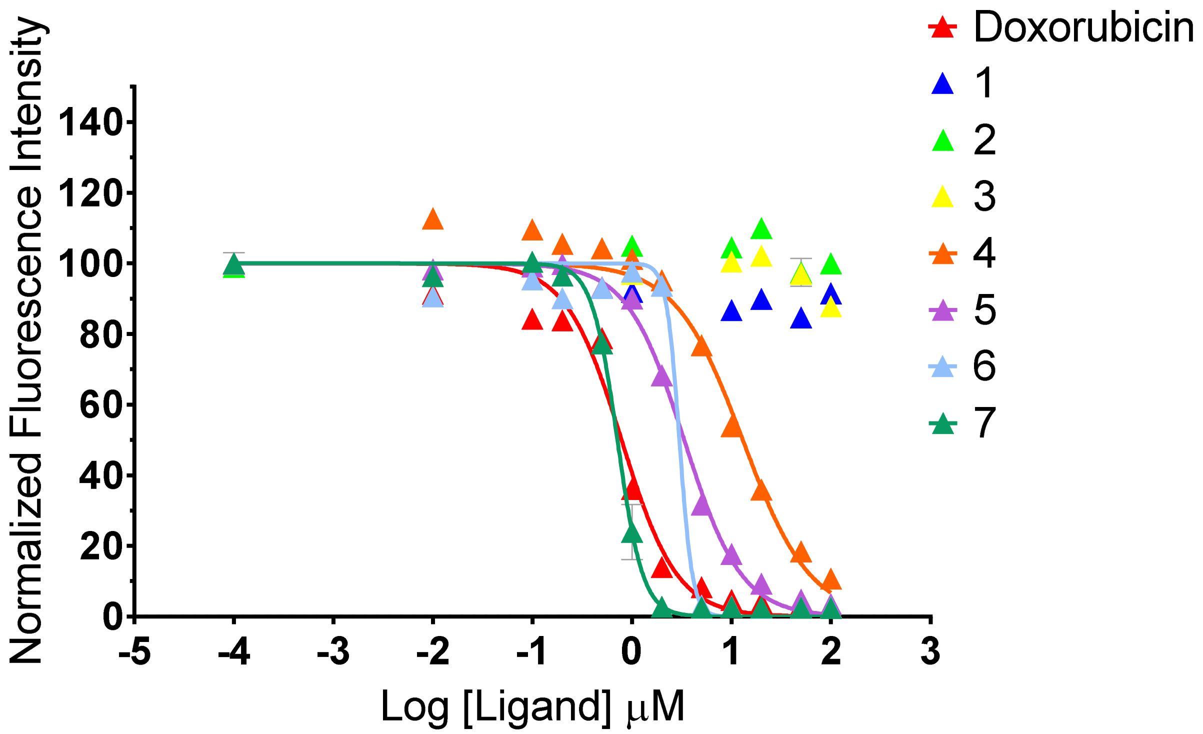

Figure S47. Effects on reductive metabolism (Alamar Blue Assay) for MCF7 after 72h incubation with doxorubicin and **1**-**7**.

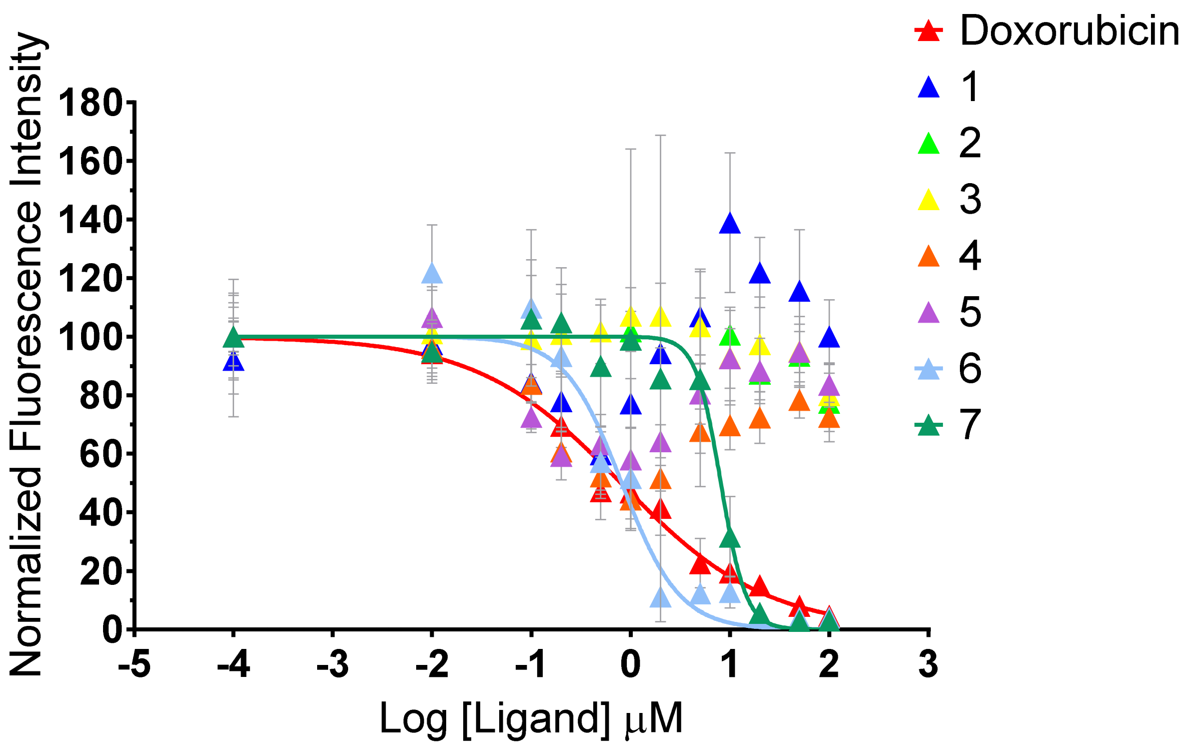

Figure S48. Calcein Assay data for MDA with doxorubicin and **1-7**. **1**, **4** and **5** appear to have a non-monotonic dose-response relationship. The very small (below assay sensitivity) number of cells surviving at high concentration of Doxorubicin, **6** and **7** invalidates metabolism/cell calculations.

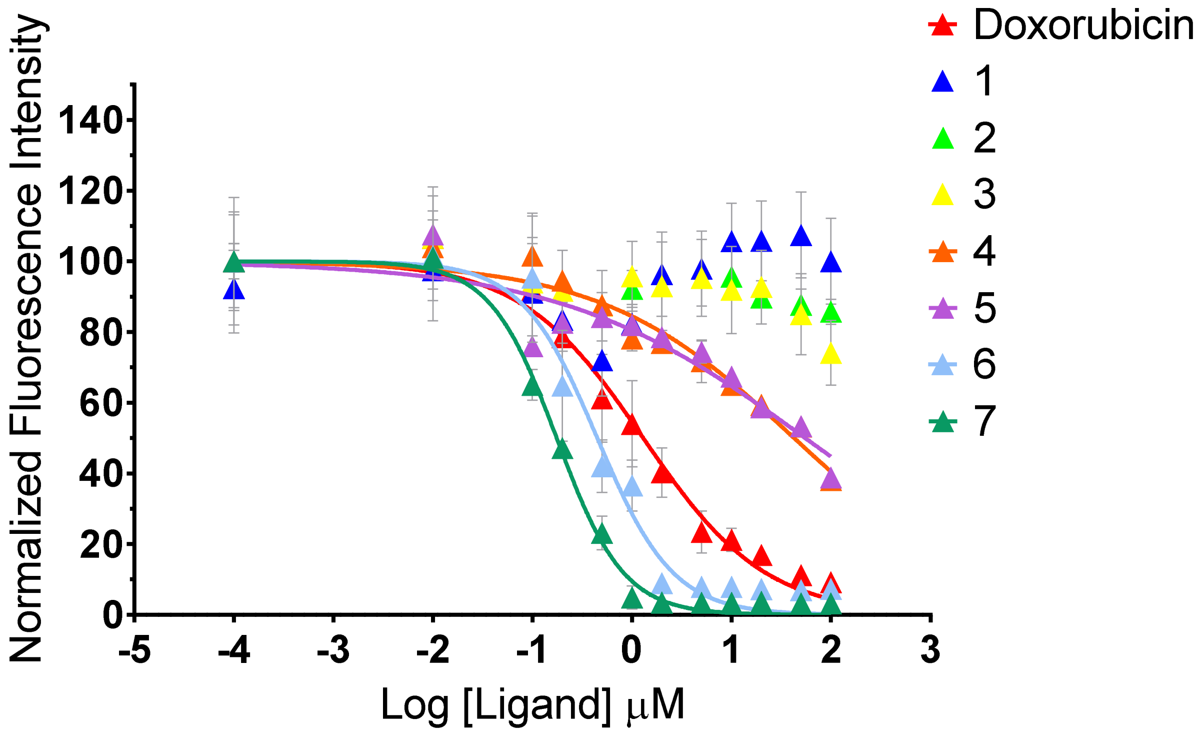

Figure S49. Effects on reductive metabolism (Alamar Blue Assay) for MDA after 72h incubation with doxorubicin and **1-7**.

Confocal Microscopy

Confocal Microscopy was carried out by the Wolfson Bioimaging Facility at the University of Bristol on a Lecia SP8 AOBS confocal laser scanning microscope attached to a Lecia DM I6000 inverted epifluorescence microscope with 'Adaptive Focus Control' to correct focus drift during time-courses (BBSRC Alert 13 capital grant (BB/L014181/1). All images were taken at 37 °C using the HeLa cell line. Cells were incubated with the 100 µM of the compound for testing at either 16h or 30min before being washed with PBS, and imaged in imaging medium. Bright-field images were taken using Transmitted Light Microscopy using a 63x 1.4 oil submersion lens. For the fluorescence images, the 405 nm diode laser was used for excitation and the emission at 535 nm was monitored. The images were analysed by Fiji software (ImageJ).

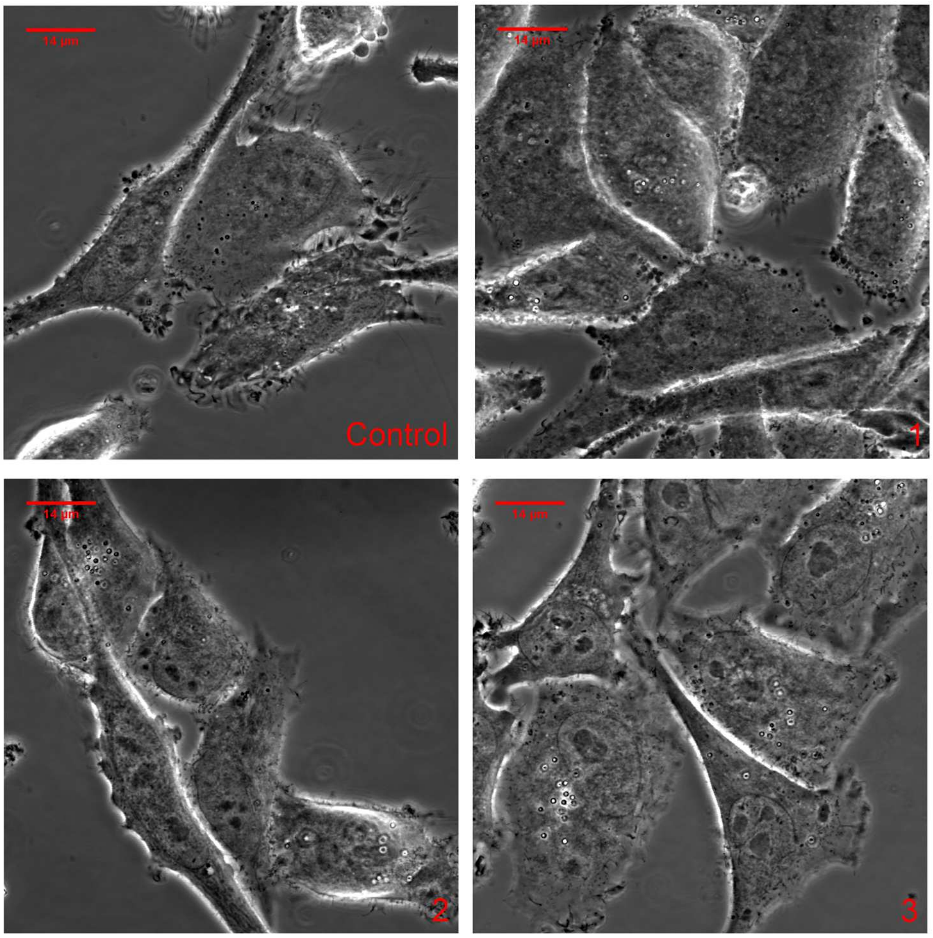

Figure S50. Bright-field transmitted light microscopy images of cells visualised in fluorescence confocal microscopy. *Top left:* Control; (1) **6** after 30 min exposure at 100 µM; (2) **3** after 30 min exposure at 100 µM; (3) **3** after 16h exposure at 100 µM.

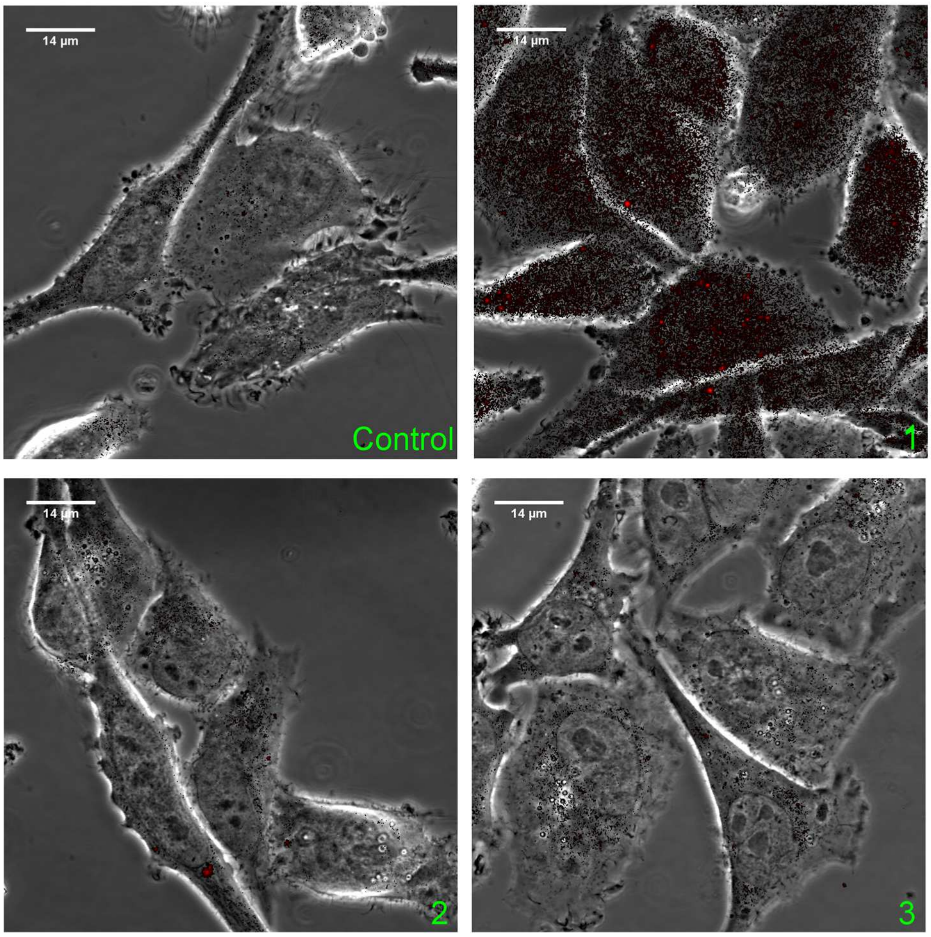

Figure S51. Overlay of bright-field transmitted light microscopy images and fluorescence confocal microscopy of cells visualised. *Top left:* Control; (1) **6** after 30 min exposure at 100 µM; (2) **3** after 30 min exposure at 100 µM; (3) **3** after 16h exposure at 100 µM.

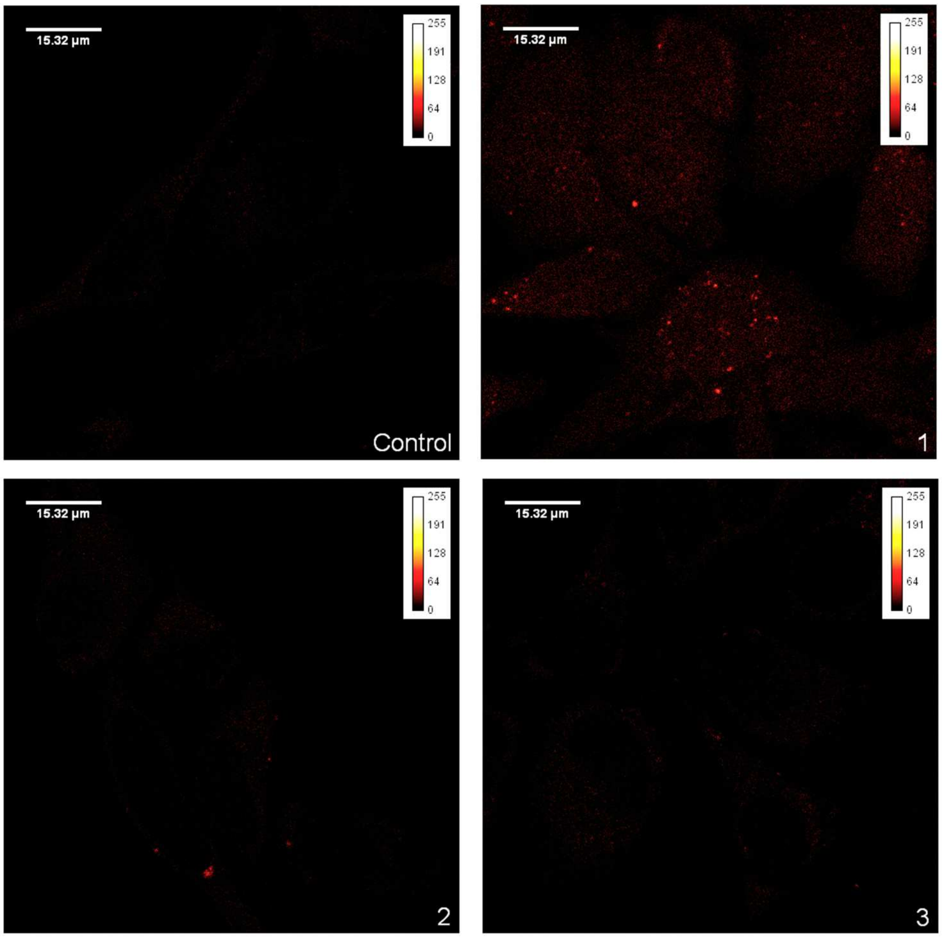

Figure S52. Fluorescence confocal microscopy images of cells visualised, monitoring the emission at 545 nm. *Top left:* Control; (1) **6** after 30 min exposure at 100 µM; (2) **3** after 30 min exposure at 100 µM; (3) **3** after 16h exposure at 100 µM.

Experimental Procedures and Data

2-azidoethyl methanesulfonate (S1)

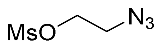

To a solution of 2-azidoethanol[13] (200 mg, 2.30 mmol) in THF (20 mL) and triethylamine (3.2 mL, 22.98 mmol) was added mesyl chloride (890 µL, 11.49 mmol) at 0 °C, and the mixture was allowed to warm to room temperature. Stirring continued for 3 h, until the reaction was determined to be complete by TLC. CH_2Cl_2 (50 mL) and saturated sodium bicarbonate (50 mL) were added, and the two layers were separated. The organic layer was washed with sodium bicarbonate (2 × 50 mL) and saturated brine (45 mL) and dried over $MgSO_4$. The Solvent was removed *in vacuo* to yield the crude product as a colorless oil (323 mg) which was used without further purification. **^{1}H NMR** (400 MHz, $CDCl_3$) δ 4.34 (2H, t, *J* = 5.0 Hz, OCH_2CH_2), 3.59 (2H, t, *J* = 5.0 Hz, $N_3CH_2CH_2$), 3.07 (3H, s, SO_2CH_3); **^{13}C NMR** (101 MHz, $CDCl_3$) δ 67.7 (OCH_2CH_2), 49.9 ($N_3CH_2CH_2$), 37.8 (SO_2CH_3). Proton and carbon NMR were consistent with literature data.[14]

3-azidopropyl methanesulfonate (S2)

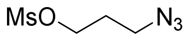

To a solution of 3-azido-1-propanol[15] (205 mg, 2.03 mmol) in THF (20 mL) and triethylamine (2.8 mL, 19.78 mmol) was added mesyl chloride (765 µL, 9.89 mmol) at 0 °C, and the mixture was allowed to warm to room temperature. Stirring continued for 3 h, until the reaction was determined to be complete by TLC. CH_2Cl_2 (50 mL) and saturated sodium bicarbonate (50 mL) were added, and the two layers were separated. The organic layer was washed with sodium bicarbonate (2 × 50 mL) and saturated brine (45 mL) and dried over $MgSO_4$. The Solvent was removed *in vacuo* to yield the crude product as a colorless oil (373 mg) which was used without further purification. **^{1}H NMR** (400 MHz, $CDCl_3$) δ 4.32 (2H, t, *J* = 6.0 Hz, OCH_2CH_2), 3.49 (2H, t, *J* = 6.4 Hz, NCH_2CH_2), 3.03 (3H, s, SO_2CH_3), 2.01 (2H, p, *J* = 6.2 Hz, $CH_2CH_2CH_2$); **^{13}C NMR** (101 MHz, $CDCl_3$) δ 66.5 (OCH_2CH_2), 47.3 ($N_3CH_2CH_2$), 37.4 (SO_2CH_3), 28.7 ($CH_2CH_2CH_2$). Proton and carbon NMR were consistent with literature data.[16]

1-(2-azidoethyl)-3-methyl-1H-imidazol-3-ium methanesulfonate (S3)

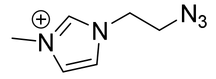

To a solution of 2-azidoethyl methanesulfonate (323 mg, 1.96 mmol) and KBF_4 (487 mg, 3.87 mmol) in acetonitrile (20 mL) was added 1-methylimidazole (500 µL, 6.27 mmol). The reaction mixture was heated to 100 °C for 24 h until the reaction was determined to be complete by TLC. After cooling to room temperature, the reaction mixture was filtered and concentrated under reduced pressure, dried under high vacuum and washed three times with a 1:1 hexane / diethyl ether (10 mL) mixture with sonication. After decantation, the solvent was removed *in vacuo* to yield the product as a colourless solid (203 mg, 42 %). **^{1}H NMR** (400 MHz, CD_3OD) δ 8.90 (1H, s, NC*H*N), 7.64 (1H, t, *J* = 1.8 Hz, NC*H*CHN), 7.58 (1H, t, *J* = 1.8 Hz, NCHC*H*N), 4.37 (2H, t, *J* = 5.6 Hz, $N_3CH_2CH_2$), 3.95 (3H, s, NCH_3), 3.84 (2H, t, J = 5.6 Hz, $NCH_2CH_2N_3$) 3.82 (3H, s, SO_2CH_3); **^{13}C NMR** (126 MHz, CD_3OD) δ 138.4 (N*C*N), 125.0 (N*C*HCHN), 123.9 (NCH*C*HN), 51.5 ($NCH_2CH_2N_3$), 49.9 ($N_3CH_2CH_2$), 36.5 (NCH_3) 34.7 (SO_2CH_3); **ESI-HRMS** for $C_6H_{10}N_5^+$ (M^+) calcd: 152.0931; found: 152.0935; **ν_{max}** / cm^{-1} (film): 3632, 3162, 3123, 2105, 1578, 1452, 1351, 1287, 1169, 1030, 840, 753.

1-(3-azidopropyl)-3-methyl-1H-imidazol-3-ium methanesulfonate (S4)

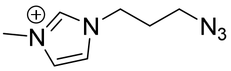

To a solution of 3-azidopropyl methanesulfonate (354 mg, 1.98 mmol) and KBF_4 (575 mg, 4.58 mmol) in acetonitrile (20 mL) was added 1-methylimidazole (500 µL, 6.27 mmol). The reaction mixture was heated to 100 °C for 16 h until the reaction was determined to be complete by TLC. After cooling to room temperature, the reaction mixture was filtered and concentrated under reduced pressure, dried under high vacuum and washed three times with a 1:1 hexane / diethyl ether (10 mL) mixture with sonication. After decantation, the solvent was removed *in vacuo* to yield the product as a colourless solid (309 mg, 62 %). **^{1}H NMR** (400 MHz, CD_3OD) δ 8.87 (1H, s, NC*H*N), 7.62 (1H, t, *J* = 1.8 Hz, NC*H*CHN), 7.56 (1H, t, *J* = 1.8 Hz, NCHC*H*N), 4.30 (2H, t, *J* = 7.1 Hz, $N_3CH_2CH_2$), 3.93 (3H, s, NCH_3), 3.79 (3H, s, SO_2CH_3), 3.44 (2H, t, *J* = 6.4 Hz, $NCH_2CH_2N_3$), 2.14 (2H, p, *J* = 6.7 Hz, $CH_2CH_2CH_2$); **^{13}C NMR** (126 MHz, CD_3OD) δ 140.7 (N*C*HN), 127.6 (N*C*HCHN), 126.2 (NCH*C*HN), 51.6 ($NCH_2CH_2N_3$), 50.7 ($N_3CH_2CH_2$), 39.0 (NCH_3), 37.0 (SO_2CH_3), 32.8 ($CH_2CH_2CH_2$); **ESI-HRMS** for $C_7H_{12}N_5^+$ (M^+) calcd: 166.1087; found: 166.1081; **ν_{max}** / cm^{-1} (film): 3631, 3162, 2919, 2850, 2101, 1573, 1462, 1285, 1168, 1017, 896, 845, 754.

1-(2-aminoethyl)-3-methyl-1H-imidazol-3-ium chloride (S5)

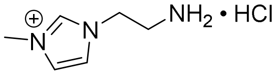

To a solution of compound **S3** (48 mg, 0.20 mmol) in acetonitrile (1 mL) and ethanol with 5% HCl (3 mL) was added palladium catalyst (10% Pd-C, 17 mg) and then the reaction mixture was hydrogenated at 1 atm for 1 h until complete consumption of the starting material was observed by TLC. The resulting mixture was then filtered through a Celite bed and washed with MeOH. The filtrate was concentrated *in vacuo* to give the final product as a yellow oil (32 mg). The product was used in the next step without further purification. **^{1}H NMR** (400 MHz, CD_3OD) δ 9.06 (1H, s, NC*H*N), 7.73 (app s, NC*H*CHN), 7.65 (app s, NCHC*H*N), 4.61 (2H, s, CHNCH_2), 3.97 (3H, s, NCH_3), 3.53 (2H, s, C$H_2$$NH_2$); **^{13}C NMR** (101 MHz, CD_3OD) δ 137.7 (N*C*HN), 124.3 (N*C*HCHN), 122.5 (NCH*C*HN), 46.6 (CHN*C*H_2), 39.0 (*C*$H_2$$NH_2$), 35.6 (N*C*$H_3$).

1-(3-aminopropyl)-3-methyl-1H-imidazol-3-ium chloride (S6)

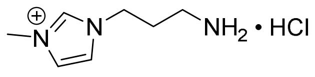

To a solution of compound **S4** (49 mg, 0.19 mmol) in ethanol with 5% HCl (3 mL) was added palladium catalyst (10% Pd-C, 13 mg) and then the reaction mixture was hydrogenated at 1 atm for 1 h until complete consumption of the starting material was observed by TLC. The resulting mixture was then filtered through a Celite bed and washed with MeOH. The filtrate was concentrated *in vacuo* to give the final product as a yellow oil (34 mg). The product was used in the next step without further purification. **^{1}H NMR** (400 MHz, D_2O) δ 8.76 (1H, s, NC*H*N), 7.50 (1H, d, *J* = 1.6 Hz, NC*H*CHN), 7.44 (1H, t, *J* = 1.7 Hz, NCHC*H*N), 4.31 (2h, t, *J* = 7.3 Hz, CHNCH_2), 3.87 (3H, s, NCH_3), 3.09 – 2.97 (2H, m, NH_2CH_2), 2.25 (2H, p, *J* = 7.6 Hz, CH_2C$H_2$$CH_2$). **^{13}C NMR** (101 MHz, D_2O) δ 135.5 (N*C*HN), 123.3 (N*C*HCHN), 121.5 (NCH*C*HN), 45.8 (CHN*C*H_2), 35.8 (*C*$H_2$$NH_2$), 35.4 (N*C*$H_3$), 26.8 ($CH_2$*C*$H_2$$CH_2$).

1-(2-azidoethyl)-6-methanesulfonate-β-D-glucopyranoside (10)

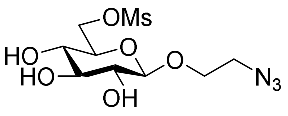

To a stirred solution of 1-(2-azidoethyl)-β-D-glucopyranoside[17] (302 mg, 1.21 mmol) in anhydrous pyridine (2.5 mL) was added methanesulfonyl chloride (103 μL, 1.34 mmol) at

-50°C dropwise over 20 mins. The mixture was kept at -50°C for 2.5 h until complete consumption of the starting material was observed by TLC. The reaction mixture was then quenched by the addition of MeOH (5 mL) and the solvent was removed *in vacuo*. The resulting residue was purified by column chromatography (95:5 to 8:2 CH_2Cl_2 / MeOH) to yield the product as a colourless solid (212 mg, 53 %). **^{1}H NMR** (400 MHz, DMSO-d_6) δ 5.30 (1H, d, *J* = 5.5 Hz, OH), 5.13 (1H, d, *J* = 5.1 Hz, OH), 5.10 (1H, d, *J* = 5.0 Hz, OH), 4.41 (1H, dd, *J* = 11.0, 1.9 Hz, H-6a), 4.29 (1H, d, *J* = 7.8 Hz, H-1), 4.27 (1H, dd, *J* = 11.1, 6.0 Hz, H-6b), 3.85 (1H, ddd, *J* = 11.2, 5.8, 4.2 Hz, OC*H*HCH$_2$), 3.68 (1H, ddd, *J* = 10.9, 5.9, 4.5 Hz, OCH*H*CH$_2$), 3.48 – 3.42 (3H, m, H-5 & CH$_2$C*H$_2$*N$_3$), 3.22 – 3.15 (4H, m, H-3 & CH$_3$), 3.09 (1H, td, *J* = 9.2, 5.5 Hz, H-4), 2.99 (1H, ddd, *J* = 9.0, 7.8, 5.1 Hz, H-2); **^{13}C NMR** (101 MHz, DMSO-d_6) δ 103.3 (C-1), 76.8 (C-3), 73.9 (C-5), 73.6 (C-2), 70.2 (C-6), 69.8 (C-4), 68.0 (O*C*H$_2$CH$_2$), 50.8 (N$_3$*C*H$_2$), 37.2 (SO$_2$*C*H$_3$); **ESI-HRMS** for $C_9H_{17}N_3NaO_8S^+$ (MNa^+) calcd: 350.0629; found: 350.0619; **ν_{max}** / cm^{-1} (film): 3417, 2933, 2096, 1349, 1275, 1169, 1078, 974, 925, 803; $[\alpha]_D^{21}$ = +0.9 (c 1.0, MeOH).

1-(2-azidoethyl)-6-methanesulfonate-α-D-mannopyranoside (11)

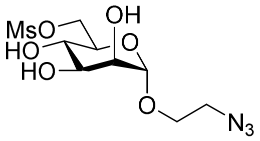

To a stirred solution of 1-(2-azidoethyl)-α-D-mannopyranoside[18] (1.28 g, 5.12 mmol) in anhydrous pyridine (10 mL) was added methanesulfonyl chloride (436 μL, 5.63 mmol) at -40°C dropwise over 20 mins. The mixture was kept at -40°C for 5.5 hours until complete consumption of the starting material was observed by TLC. The reaction mixture was then quenched by the addition of aq. sat. $NaHCO_3$ (5 mL) and the solvent was removed *in vacuo*. The resulting residue was purified by column chromatography (98:2 to 9:1 CH_2Cl_2 / MeOH) to yield the product as a colourless solid (877 mg, 52 %). **^{1}H NMR** (400 MHz, D_2O) δ 4.93 (1H, d, *J* = 1.7 Hz, H-1), 4.61 (1H, dd, *J* = 11.4, 2.2 Hz, H-6a), 4.53 (1H, dd, *J* = 11.4, 5.4 Hz, H-6b), 4.00 (1H, dd, *J* = 3.4, 1.8 Hz, H-2), 3.97 – 3.89 (2H, m, H-5 & OC*H*HCH$_2$), 3.86 (1H, dd, *J* = 9.7, 3.4 Hz, H-3), 3.77 – 3.70 (2H, m, H-4 & OCH*H*CH$_2$), 3.59 – 3.46 (2H, m, CH$_2$C*H$_2$*N$_3$), 3.27 (3H, s, SO$_2$C*H$_3$*); **^{13}C NMR** (101 MHz, D_2O) δ 100.0 (C-1), 70.5 (C-5), 70.2 (C-3), 69.7 (C-2), 69.4 (C-6), 66.5 (O*C*H$_2$CH$_2$), 66.0 (C-4), 50.1 (N$_3$*C*H$_2$), 36.5 (SO$_2$*C*H$_3$); **ESI-HRMS** for $C_9H_{17}N_3NaO_8S^+$ (MNa^+) calcd: 350.0629; found: 350.0631; **ν_{max}** / cm^{-1} (film): 3391, 2937, 2106, 1636, 1451, 1414, 1347, 1172, 1135, 1060, 965, 836; $[\alpha]_D^{22}$ = +41.0 (c 0.9, MeOH).

1-(2-azidoethyl)-6-(3-methyl-1H-imidazol-3-ium)-6-deoxy-β-D-glucopyranoside methanesulfonate (12)

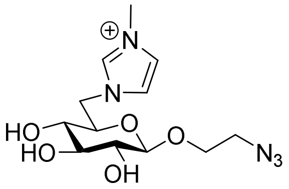

To a solution of sugar **10** (35 mg, 0.11 mmol) in acetonitrile (1 mL) and water (0.5 mL) was added 1-methylimidazole (50 µL, 0.63 mmol). The reaction mixture was stirred and heated to reflux for 48 h until the reaction was determined to be complete by TLC. After cooling to room temperature, the reaction mixture was filtered and concentrated *in vacuo*, dried under high vacuum and washed three times with a 1:1 hexane / diethyl ether (10 mL) mixture with sonication. After decantation, the oil was dried under vacuum, and purified by column chromatography (9:1 CH_2Cl_2 / MeOH then 65:35:2 CH_2Cl_2 / MeOH / H_2O) to yield the product as a colourless solid (28 mg, 62 %). **^{1}H NMR** (400 MHz, D_2O) δ 8.78 (1H, s, NC*H*N), 7.53 (1H, t, *J* = 1.7 Hz, NC*H*CHN), 7.48 (1H, t, *J* = 1.5 Hz, NCHC*H*N), 4.67 (1H, dd, *J* = 14.6, 2.5 Hz, H-6a), 4.49 (1H, d, *J* = 8.0 Hz, H-1), 4.42 (1H, dd, *J* = 14.7, 7.5 Hz, H-6b), 3.96 – 3.88 (4H, m, NCH_3 & OC*H*HCH_2), 3.85 – 3.74 (2H, m, OCH*H*CH_2 & H-5), 3.56 – 3.43 (3H, m, $OCH_2CH_2N_3$ & H-3), 3.30 – 3.20 (2H, m, H-2 & H-4), 2.81 (3H, s, SO_2CH_3); **^{13}C NMR** (100 MHz, D_2O) δ 136.8 (N*C*HN), 123.4 (N*C*HCHN), 123.1 (NCH*C*HN), 102.2 (C-1), 75.3 (C-3), 73.1 (C-5), 72.8 (C-2), 70.3 (C-4), 68.6 (OCH_2CH_2), 50.3 (N_3CH_2), 49.8 (C-6), 38.4 (SO_2CH_3), 35.7 (NCH_3); **ESI-HRMS** for $C_{12}H_{20}N_5O_5^+$ (MH^+) calcd: 314.1459; found: 314.1460; **ν_{max}** / cm^{-1} (film): 3367, 2918, 2165, 2107, 1644, 1577, 1425, 1167, 1040, 834, 775; $[\alpha]_D^{22}$ = -6.1 (c 0.2, MeOH).

1-(2-azidoethyl)-6-(3-methyl-1H-imidazol-3-ium methanesulfonate)-6-deoxy-α-D-mannopyranoside (13)

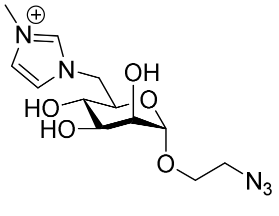

To a solution of sugar **11** (228 mg, 0.70 mmol) in DMF (5 mL) was added 1-methylimidazole (277 µL, 3.48 mmol). The reaction mixture was stirred and heated to 80°C for 48 h until complete consumption of the starting material was observed by TLC. After cooling to room temperature, the reaction mixture was concentrated under reduced pressure, dried under high

vacuum and washed three times with a 1:1 hexane / diethyl ether (10 mL) mixture with sonication. After decantation, the oil was dried under vacuum, and purified by column chromatography (9:1 CH_2Cl_2 / MeOH then 65:35:2 CH_2Cl_2 / MeOH / H_2O) to yield the product as a colourless solid (125 mg, 44 %). **^{1}H NMR** (400 MHz, D_2O) δ 8.83 (1H, s, NC*H*N), 7.59 (1H, t, *J* = 1.9 Hz, NC*H*CHN), 7.48 (1H, t, *J* = 1.9 Hz, NCHC*H*N), 4.90 (1H, d, *J* = 1.7 Hz, H-1), 4.65 (1H, dd, *J* = 14.6, 2.4 Hz, H-6a), 4.41 (1H, dd, *J* = 14.6, 8.0 Hz, H-6b), 3.98 (1H, dd, *J* = 3.4, 1.7 Hz, H-2), 3.94 – 3.87 (4H, m, H-5 & NCH_3), 3.84 (1H, dd, *J* = 9.6, 3.4 Hz, H-3), 3.62 (1H, ddd, *J* = 10.5, 5.9, 2.9 Hz, OC*H*HCH_2), 3.57 – 3.43 (3H, m, OCH*H*CH_2, H-4 & CH_2C*H*HN_3), 3.40 – 3.32 (1H, m, CH_2CH*H*N_3), 2.81 (3H, s, SO_2CH_3); **^{13}C NMR** (101 MHz, D_2O) δ 136.9 (N*C*HN), 123.4 (N*C*HCHN), 123.0 (NCH*C*HN), 99.9 (C-1), 70.8 (C-5), 70.1 (C-3), 69.7 (C-2), 67.4 (C-4), 66.3 (O*C*H_2CH_2), 50.0 (N_3*C*H_2), 49.9 (C-6), 38.4 (SO_2*C*H_3), 35.7 (N*C*H_3); **ESI-HRMS** for $C_{12}H_{20}N_5O_5^+$ (M^+) calcd: 314.1459; found: 314.1473; **ν$_{max}$** / cm^{-1} (film): 3382, 2932, 2104, 1652, 1548, 1426, 1170, 1133, 1103, 1041, 961, 894, 776; $[\alpha]_D^{22}$ = +24.3 (c 1.9, MeOH).

bis-*N,N'*-(*N,N*-dimethylpropylamine)-1,4,5,8-naphthalenetetracarboxylic diimide (S7)

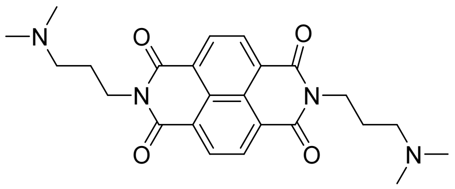

To a solution of 3-dimethylaminopropylamine (0.235 ml, 1.868 mmol) in toluene (10 ml) was added 1,4,5,8-Naphthalenetetracarboxylic dianhydride (NTCDA) (102 mg, 0.380 mmol) to give a yellow suspension. The reaction was heated to 145°C for 18 h until the reaction was determined to be complete by LC-MS (MH = 437). The solvent was removed *in vacuo* and purified by flash chromatography (CH_2Cl_2 to 9:1 CH_2Cl_2 / MeOH) to yield the product as a yellow solid (115 mg, 69 %). **^{1}H NMR** (400 MHz, $CDCl_3$) δ 8.76 (4H, s, Ar-*H*), 4.27 (4H, t, *J* = 7.5 Hz, C(O)NCH_2CH_2), 2.44 (4H, t, *J* = 7.1 Hz, CH_3NCH_2CH_2), 2.23 (12H, s, NCH_3), 1.92 (4H, p, *J* = 7.3 Hz, CH_2CH_2CH_2). **^{13}C NMR** (101 MHz, $CDCl_3$) δ 162.4 (C=O), 130.4 (Ar-*C*H), 126.2 (Ar-*C*), 56.7 (CH_3N*C*H_2CH_2), 44.8 (N*C*H_3), 38.8 (C(O)N*C*H_2CH_2), 25.5 (CH_2*C*H_2CH_2). Proton and carbon NMR were consistent with literature data.[19]

bis-*N,N'*-(*N,N*-dimethylpropan-1-aminium trifluoroacetate)-1,4,5,8-naphthalenetetracarboxylic diimide (7)

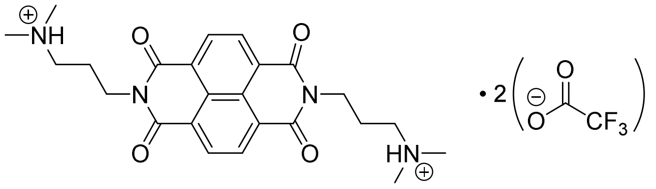

To a solution of **S7** (28 mg, 0.07 mmol) in water (2 ml) was added TFA (100 μL) and stirred at rt for 1 min until all of the yellow solid had dissolved. The sample was then purified by reverse-phase flash chromatography (95:5 to 5:95 water + 0.1 % TFA/ MeCN) to yield the product as a colourless solid (32 mg, 74 %). **^{1}H NMR** (400 MHz, D_2O) δ 8.65 (4H, s, Ar-*H*), 4.26 (4H, t, J = 6.8 Hz, C(O)NC*H2*), 3.34 - 3.25 (4H, m, CH_3NC*H2*), 2.93 (12H, s, NC*H3*), 2.21 (4H, q, J = 7.3 Hz, CH_2C*H2*CH_2). **^{13}C NMR** (101 MHz, D_2O) δ 163.9 (*C*=O), 130.6 (Ar-*C*H), 125.7 (Ar-*C*), 54.9 (CH_3NCH_2), 42.3 (NCH_3), 37.1 (C(O)NCH_2), 22.4 ($CH_2CH_2CH_2$). **^{19}F NMR** (376 MHz, D_2O) δ -75.60 (TFA). **ESI-HRMS** for $C_{24}H_{30}N_4O_4^{2+}$ (M^+) calcd: 219.1128; found: 219.1136; **ν_{max}** / cm^{-1} (film): 2973, 2886, 1702, 1663, 1582, 1455, 1377, 1336, 1247, 1174, 1126, 1054, 830, 799, 768, 720; **HPLC** (280 nm) t_R: 18.4 min, purity: 99 %.

bis-*N,N'*-(3-(4-methyl-1-piperazine-1,2-diium trifluoroacetate)propyl)-1,4,5,8-naphthalenetetracarboxylic diimide (6)

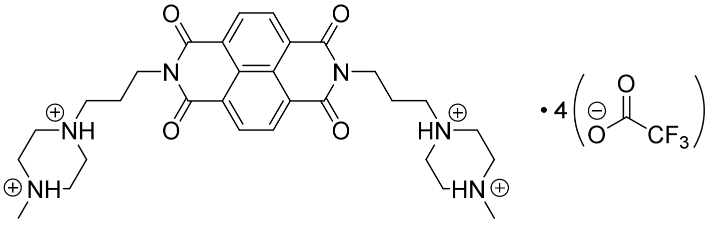

To a solution of 1-(3-aminopropyl)-4-methylpiperazine (160 μL, 0.94 mmol) in toluene (10 mL) was added NTCDA (51 mg, 0.19 mmol). The reaction mixture was heated to reflux for 17 h until LC-MS showed the reaction was complete (MH = 548). The solution was cooled to room temperature, the solvent was removed *in vacuo* and purified by reverse-phase flash chromatography (90:10 to 5:95 water + 0.1 % TFA/ MeCN) to yield the product as a light pink solid (139 mg, 74 %). **^{1}H NMR** (400 MHz, D_2O) δ 8.60 (4H, s, Ar-*H*), 4.27 (4H, t, J = 6.9 Hz, (C=O)NC*H2*CH_2), 3.71 (16H, br s, NC*H2*C*H2*N), 3.50 - 3.43 (4H, m, NC*H2*CH_2CH_2NC(O)), 3.05 (6H, s, NC*H3*), 2.26 (4H, p, J = 7.8 Hz, CH_2C*H2*CH_2); **^{13}C NMR** (101 MHz, D_2O) δ 164.1 (C=O), 162.8 (q, *J* = 35.5 Hz, CF_3C=O), 131.1 (Ar-*C*H), 126.0 (Ar-

C), 116.3 (q, J = 291.8 Hz, CF3), 54.6 (NCH2CH2CH2NC(O)), 50.3 (NCH2CH2N), 48.8 (NCH2CH2N), 42.8 (NCH3), 37.7 ((C=O)NCH2CH2), 22.3 (CH2CH2CH2); **^{19}F NMR** (376 MHz, D2O) δ -75.58 (TFA); **ESI-HRMS** for $C_{30}H_{40}N_6O_4^{2+}$ (M^+) calcd: 274.1550; found: 274.1158; **νmax** / cm^{-1} (film): 3008, 1702, 1672, 1659, 1581, 1454, 1406, 1375, 1247, 1194, 1170, 1103, 1074, 1040, 1017, 972, 962, 885, 850, 832, 795, 764, 721, 626; **HPLC** (280 nm) t_R: 26.1 min, purity: >99 %.

bis-*N,N′*-(1-(2-ethyl)-3-methyl-1H-imidazol-3-ium chloride)-1,4,5,8-naphthalenetetracarboxylic diimide (4)

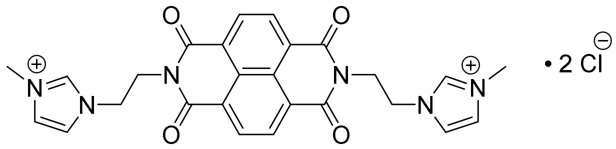

To a solution of amine **S5** (54 mg, 0.25 mmol) and pyridine (100 µL, 0.92 mmol) in EtOH (5 mL) and acetonitrile (2 mL) was added NTCDA (23 mg, 0.08 mmol). The reaction mixture was heated to reflux for 4 h until complete consumption of the starting materials was observed by TLC. After cooling to room temperature, the brown solid was filtered off, washed with EtOH (5 mL) and dried under vacuum to yield the product as a colourless solid (11 mg, 23 %). **^{1}H NMR** (400 MHz, DMSO-d_6) δ 9.21 (2H, s, NC*H*N), 8.66 (4H, s, Ar-H) 7.87 (2H, app s, NC*H*CHN), 7.66 (2H, app s, NCHC*H*N), 4.55 (4H, s, NC*H2*CH2N), 4.49 (4H, app s, NCH2C*H2*N), 3.80 (6H, app s, C*H3*); **^{13}C NMR** (126 MHz, DMSO-d_6) δ 163.29 (C=O), 137.69 (NCHN), 130.98 (CH), 126.79 (C), 124.3 (C), 123.91 (NCHCHN), 123.55 (NCHCHN), 47.87 (NCH2CH2N), 40.99 (NCH2CH2N), 36.20 (NCH3); **ESI-HRMS** for $C_{26}H_{24}N_6O_4^{2+}$ (M^{2+}) calcd: 242.0924; found: 242.0919; **νmax** / cm^{-1} (film): 3395, 3164, 31101705, 1667, 1582, 1456, 1379, 1340, 1255, 1168, 1019, 889, 802, 767, 726, 655; **HPLC** (280 nm) t_R: 21.0 min, purity: 95 %.

bis-*N,N′*-(1-(3-propyl)-3-methyl-1H-imidazol-3-ium chloride)-1,4,5,8-naphthalenetetracarboxylic diimide (5)

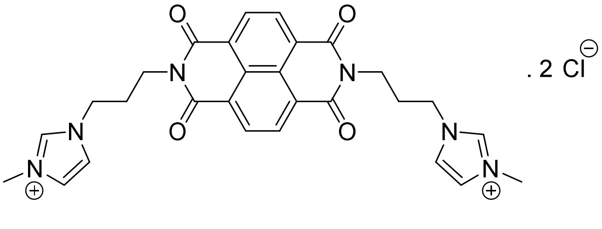

To a solution of amine **S6** (57 mg, 0.25 mmol) and pyridine (100 µL, 0.92 mmol) in EtOH (5 mL) and acetonitrile (2 mL) was added NTCDA (23 mg, 0.08 mmol). The reaction mixture

was heated to reflux for 19 h until complete consumption of the starting materials was observed by TLC. After cooling to room temperature, the brown solid was filtered off, washed with EtOH (5 mL) and dried under vacuum to yield the product as a colourless solid (25 mg, 51 %). **^{1}H NMR** (400 MHz, DMSO-*d*$_6$) δ 9.10 (2H, s, NC*H*N), 8.70 (4H, s, Ar-H), 7.79 (2H, s, NC*H*CHN), 7.70 (2H, s, NCHC*H*N), 4.30 (4H, t, *J* = 7.3 Hz, NC*H*$_2$CH$_2$), 4.12 (4H, t, *J* = 6.6 Hz, CH$_2$C*H*$_2$N), 3.85 (6H, s, CH$_3$), 2.25 (4H, p, *J* = 6.7 Hz, CH$_2$C*H*$_2$CH$_2$); **^{13}C NMR** (126 MHz, DMSO-*d*$_6$) δ 163.37 (C=O), 137.12 (N*C*HN), 130.89 (CH), 126.89 (C), 126.68 (C), 124.05 (NCH*C*HN), 122.71 (N*C*HCHN), 47.34 (N*C*H$_2$CH$_2$), 37.65 (CH$_2$*C*H$_2$N), 36.21 (N*C*H$_3$), 28.71 (CH$_2$*C*H$_2$CH$_2$); **ESI-HRMS** for $C_{28}H_{28}N_6O_4^{2+}$ (M^{2+}) calcd: 256.1081; found: 256.1076; **ν$_{max}$** / cm^{-1} (film): 3410, 3164, 3113, 2975, 1701, 1657, 1579, 1460, 1336, 1249, 1173, 1056, 970, 852, 768, 746, 660; **HPLC** (280 nm) *t*$_R$: 25.6 min, purity: 96 %.

bis-*N,N'*-(2-ethyl-β-D-glucopyranoside)-1,4,5,8-naphthalenetetracarboxylic diimide (1)

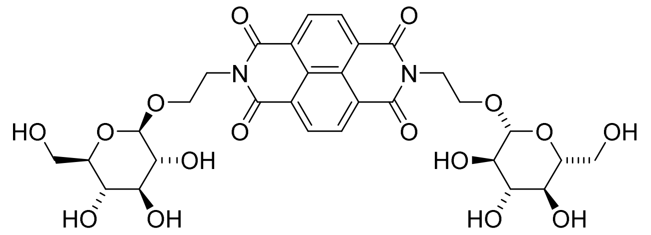

To a solution of 2-aminoethyl-β-D-Glucopyranoside[18] (41 mg, 0.18 mmol) in anhydrous EtOH (5 mL) was added NTCDA (22 mg, 0.08 mmol). The reaction mixture was stirred and heated to reflux for 16 h until the reaction was determined to be complete by MALDI (MNa$^+$ = 701). After cooling to room temperature, the mixture was cooled to 0°C and the resulting precipitate was filtered, washed with cold ethanol (10 mL) and diethyl ether (10 mL), to yield the product as a colourless solid (20 mg, 37 %). **^{1}H NMR** (500 MHz, D$_2$O) δ 8.48 (1H, s, Ar-H), 4.53 - 4.46 (2H, m, CH$_2$C*H*HN), 4.48 (2H, d, *J* = 7.9 Hz, H-1), 4.39 – 4.30 (2H, m, CH$_2$CH*H*N), 4.27 – 4.19 (2H, m, H-6a), 4.05 (2H, dt, *J* = 10.6, 5.0 Hz, H-6b), 3.73 (2H, dd, *J* = 12.2, 1.9 Hz, OC*H*HCH$_2$), 3.50 (2H, dd, *J* = 12.3, 5.9 Hz, OC*H*HCH$_2$), 3.47 – 3.38 (4H, m, H-5 & H-3), 3.32 – 3.21 (4H, m, H-4 & H-2); **^{13}C NMR** (126 MHz, CDCl$_3$) δ 163.75 (C=O), 130.96 (CH), 125.72 (C), 125.52 (C), 102.36 (C-1), 75.79 (C-5), 75.56 (C-3), 72.98 (C-2), 69.53 (C-4), 66.68 (C-6), 60.65 (O*C*H$_2$CH$_2$N), 40.11 (N*C*H$_2$CH$_2$O); **ESI-HRMS** for $C_{30}H_{34}N_2NaO_{16}^+$ (MNa$^+$) calcd: 701.1801; found: 701.1800; **ν$_{max}$** / cm^{-1} (film): 3342, 2955, 2922, 2853, 1728, 1635, 1461, 1377, 1260, 1071; $[\alpha]_D^{23}$ = -280 (c 0.1, MeOH); **HPLC** (280 nm) *t*$_R$: 18.3 min, purity: 98 %.

bis-*N,N′*-(2-ethyl-6-(3-methyl-1H-imidazol-3-ium formate)-6-deoxy-β-D-glucopyranoside)-1,4,5,8-naphthalenetetracarboxylic diimide (2)

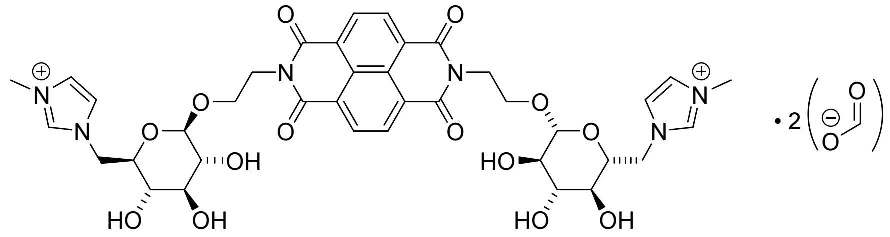

To a solution of sugar **12** (48 mg, 0.15 mmol) in ethanol with 5% HCl (5 mL) was added palladium catalyst (10% Pd-C, 15 mg). The reaction mixture was left stirring at room temperature under a under a H_2 atmosphere at 1 atm for 2 h until complete consumption of starting materials was observed by TLC. The resulting mixture was then filtered through a Celite bed and washed with MeOH. The filtrate was concentrated *in vacuo* to give the intermediate amine as a yellow oil (45 mg). **ESI-HRMS** for $C_{12}H_{22}N_3O_5^+$ (MH^+) calcd: 288.1554; found: 288.1557. Without further purification, a portion of the dry mixture (15 mg) was directly taken to the next step and NTCDA (4 mg, 0.02 mmol) and triethylamine (50 μL, 0.36 mmol) were added and dissolved in anhydrous EtOH (5 mL). The reaction mixture was stirred and heated to reflux. After 16 h, a green fluorescent spot was observed by TLC and MALDI analysis revealed formation of the desired product (M^+ = 808). After cooling to room temperature, the solvent was removed *in vacuo* and the resulting dark oil was purified by preparative reverse-phase HPLC to yield the product as a light brown solid (7.4 mg, 55 %). **^{1}H NMR** (500 MHz, D_2O) δ 8.78 (2H, s, NC*H*N), 8.71 (4H, s, Ar-H), 8.46 (2H, br s, *H*COO), 7.48 (4H, s, NC*H*C*H*N), 4.62 (2H, d, *J* = 13.1 Hz, H-6a), 4.47 (2H, d, *J* = 7.9 Hz, H-1), 4.45 – 4.36 (4H, m, OCH_2CH_2N), 4.30 (2H, dd, *J* = 14.7, 7.8 Hz, H-6b), 4.10 (2H dt, *J* = 11.6, 6.1 Hz, OC*H*HCH_2N), 4.00 – 3.91 (8H, m, OC*H*HCH_2N & NCH_3), 3.79 – 3.72 (2H, m, H-5), 3.47 (2H, t, *J* = 9.2 Hz, H-3), 3.22 (2H, t, *J* = 8.9 Hz, H-2), 3.18 (2H, t, *J* = 9.3 Hz, H-4); **^{13}C NMR** (126 MHz, D_2O) δ 171.0 (H*C*OO), 164.3 (C=O), 136.8 (N*C*HN), 131.0 (CH), 126.2 (C), 126.1 (C), 123.5 (N*C*HCHN), 123.1 (NCH*C*HN), 102.5 (C-1), 75.2 (C-3), 73.3 (C-5), 72.8 (C-2), 70.4 (C-4), 66.6 (OCH_2CH_2N), 49.9 (C-6), 40.0 (NCH_2CH_2O), 35.8 (NCH_3); **ESI-HRMS** for $C_{38}H_{44}N_6O_{14}^{2+}$ (M^{2+}) calcd: 404.1452; found: 404.1456; **ν_{max}** / cm^{-1} (film): 3368, 1706, 1665, 1583, 1345, 1080; $[\alpha]_D^{21}$ = +21 (c 0.1, MeOH); **HPLC** (280 nm) t_R: 25.5 min, purity: 96 %.

bis-*N,N'*-(2-ethyl-6-(3-methyl-1H-imidazol-3-ium formate)-6-deoxy-α-D-mannopyranoside)-1,4,5,8-naphthalenetetracarboxylic diimide (3)

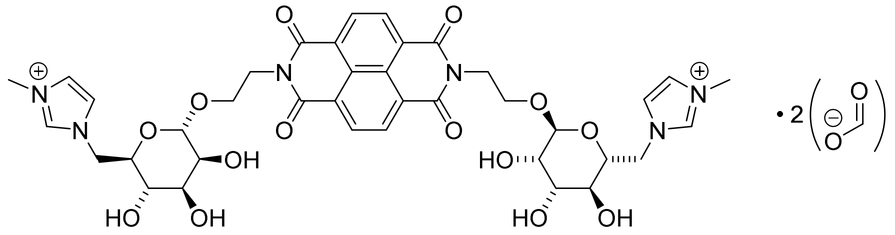

To a solution of sugar **13** (55 mg, 0.14 mmol) in ethanol with 5% HCl (10 mL) was added palladium catalyst (10% Pd-C, 17 mg). The reaction mixture was hydrogenated at 1 atm for 1.5 h until complete consumption of the starting material was observed by TLC. The resulting mixture was then filtered through a Celite bed and washed with MeOH. The filtrate was concentrated *in vacuo* to give the intermediate amine as a yellow oil (49 mg). To the crude intermediate (16 mg), NTCDA (4 mg, 0.02 mmol) and triethylamine (20 μL, 0.15 mmol) was added and dissolved in anhydrous EtOH (5 mL). The reaction mixture was stirred and heated to 80°C. After 17 h, a green fluorescent spot was observed by TLC and MALDI analysis revealed formation of the desired product (M^+ = 808). After cooling to room temperature, the solvent was removed *in vacuo* and the resulting dark oil was purified by preparative reverse-phase HPLC to yield the product as a light brown solid (8.2 mg, 60 %). **^{1}H NMR** (500 MHz, D_2O) δ 8.82 (2H, s, NC*H*N), 8.60 (4H, s, Ar-H), 8.40 (2H, br s, *H*COO), 7.49 (2H, t, *J* = 1.8 Hz, NC*H*CHN), 7.46 (2H, t, *J* = 1.8 Hz, NCHC*H*N), 4.98 (2H, d, *J* = 1.6 Hz, H-1), 4.52 (2H, dd, *J* = 14.6, 2.3 Hz, H-6a), 4.42 (2H, ddd, *J* = 13.9, 7.9, 4.9 Hz, OCH_2C*H*HN), 4.34 (2H, dd, *J* = 14.6, 8.0 Hz, H-6b), 4.22 (2H, dt, *J* = 14.0, 4.9 Hz, OCH_2CH*H*N), 3.96 (6H, s, NC*H*$_3$), 3.90 (1H, dd, *J* = 3.4, 1.7 Hz, H-2), 3.79 (2H, dt, *J* = 10.9, 4.9 Hz, OC*H*HCH_2N), 3.76 – 3.68 (4H, m, H-5 & OCH*H*CH_2N), 3.66 (2H, dd, *J* = 9.6, 3.4 Hz, H-3), 3.48 (2H, t, *J* = 9.7 Hz, H-4); **^{13}C NMR** (126 MHz, D_2O) δ 169.4 (*H*COO), 163.8 (C=O), 136.9 (N*C*HN), 131.1 (CH), 125.9 (C), 125.8 (C), 123.5 (N*C*HCHN), 122.9 (N*C*HCHN), 99.3 (C-1), 70.9 (C-5), 70.2 (C-3), 69.7 (C-2), 67.2 (C-4), 63.7 (NCH_2*C*H_2O), 49.8 (C-6), 39.5 (N*C*H_2CH_2O), 35.8 (N*C*H_3); **ESI-HRMS** for $C_{38}H_{44}N_6O_{14}^{2+}$ (M^{2+}) calcd: 404.1452; found: 404.1468; **ν_{max}** / cm^{-1} (film): 3350, 3159, 2919, 2781, 2699, 1703, 1666, 1597, 1455, 1342, 1243, 1169, 1134, 1049, 961, 766; $[\alpha]_D^{22}$ = 34.2 (c 0.3, MeOH); **HPLC** (280 nm) t_R: 25.9 min, purity: 99 %.

NMR Spectra of Novel Compounds

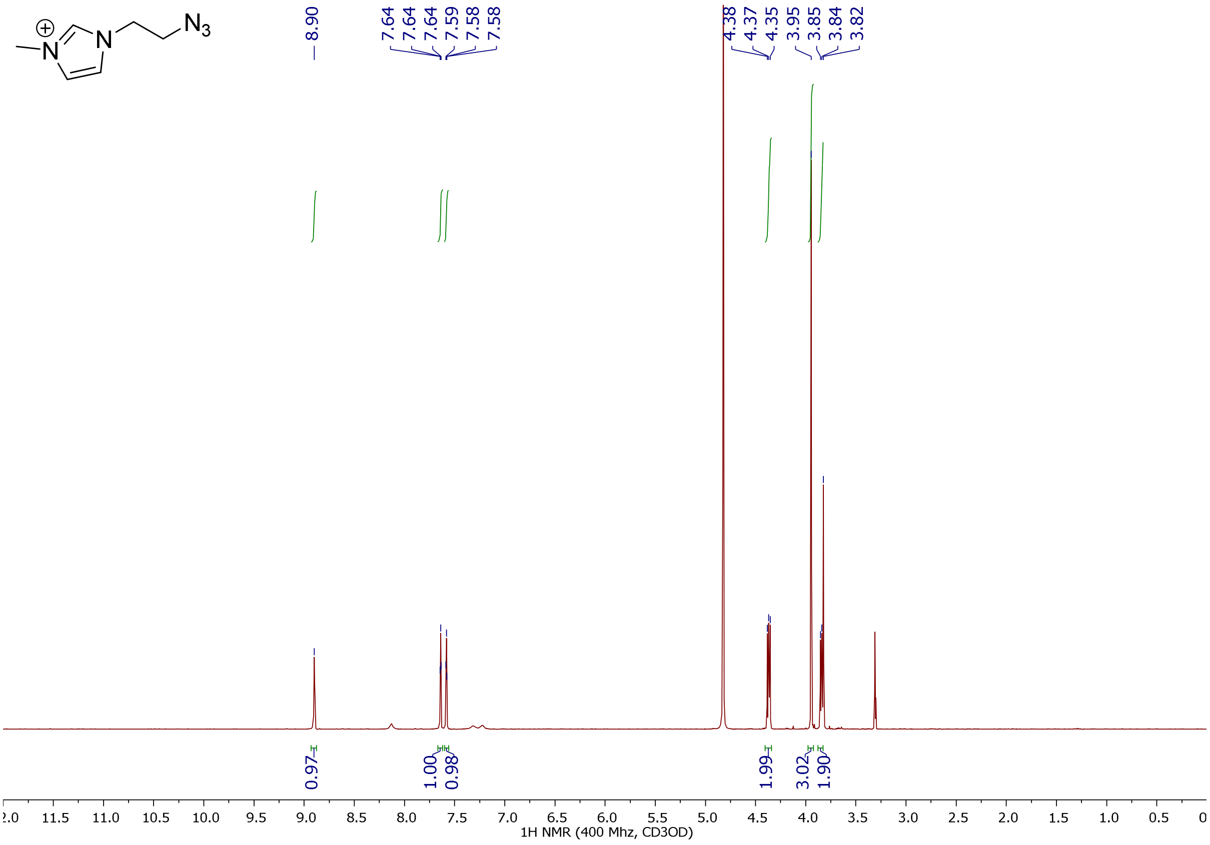

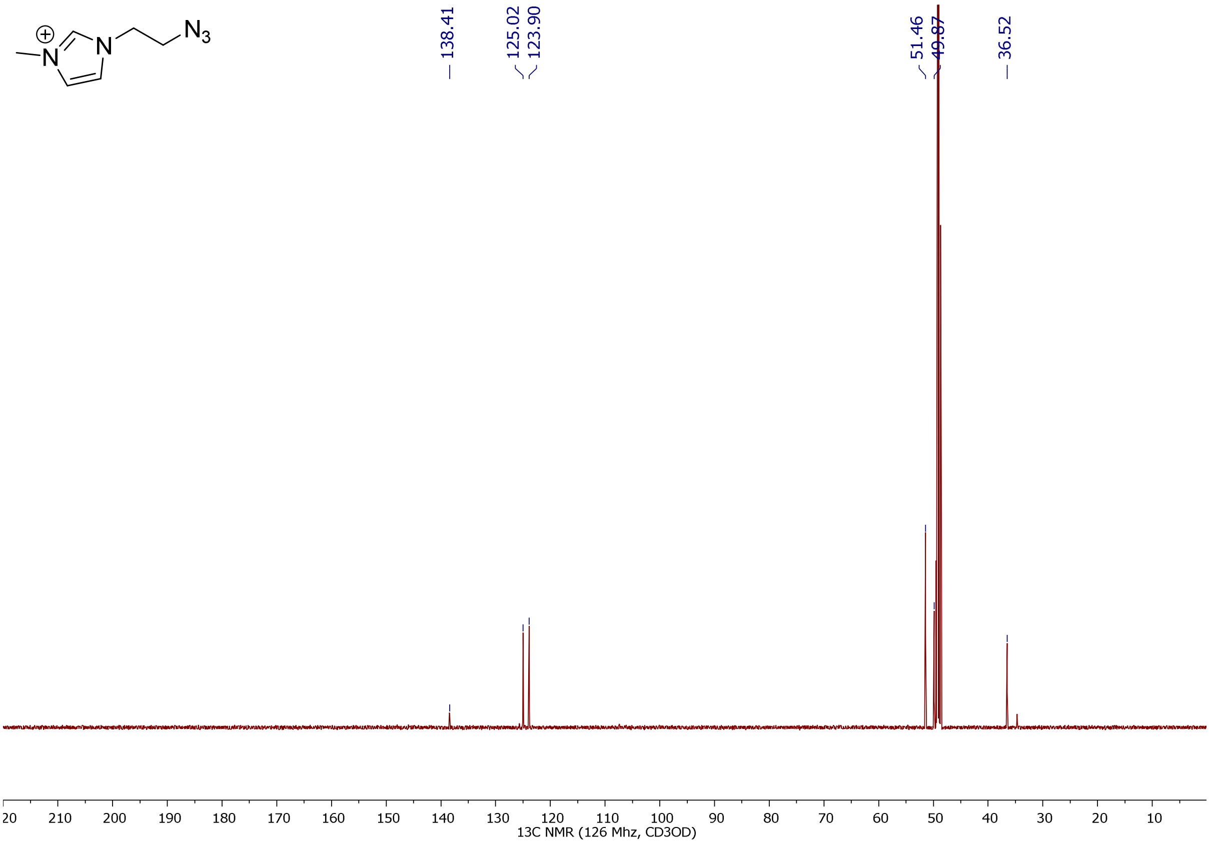

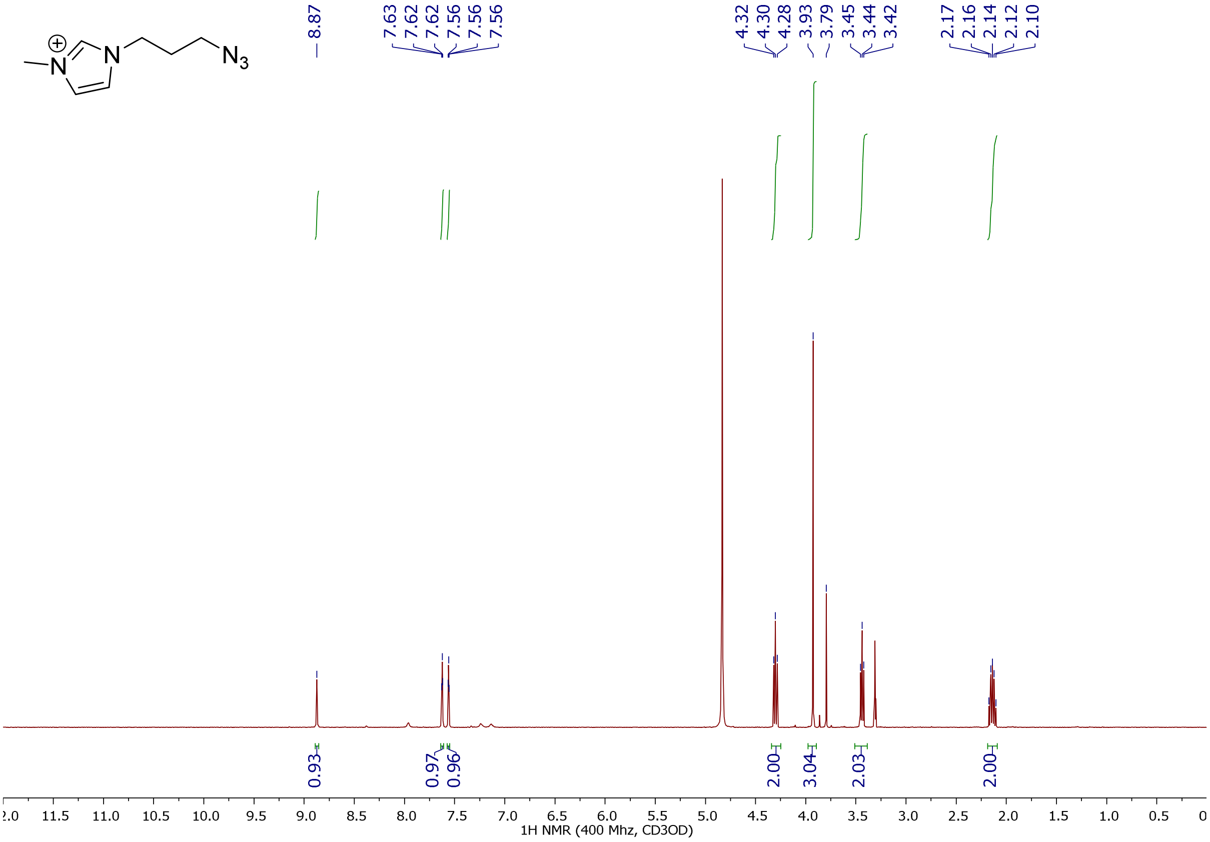
N3
8.87
7.63
7.62
7.62
7.56
7.56
7.56
4.32
4.30
4.28
3.93
3.79
3.45
3.44
3.42
2.17
2.16
2.14
2.12
2.10
0.93
0.97
0.96
2.00
3.04
2.03
2.00
1H NMR (400 Mhz, CD3OD)

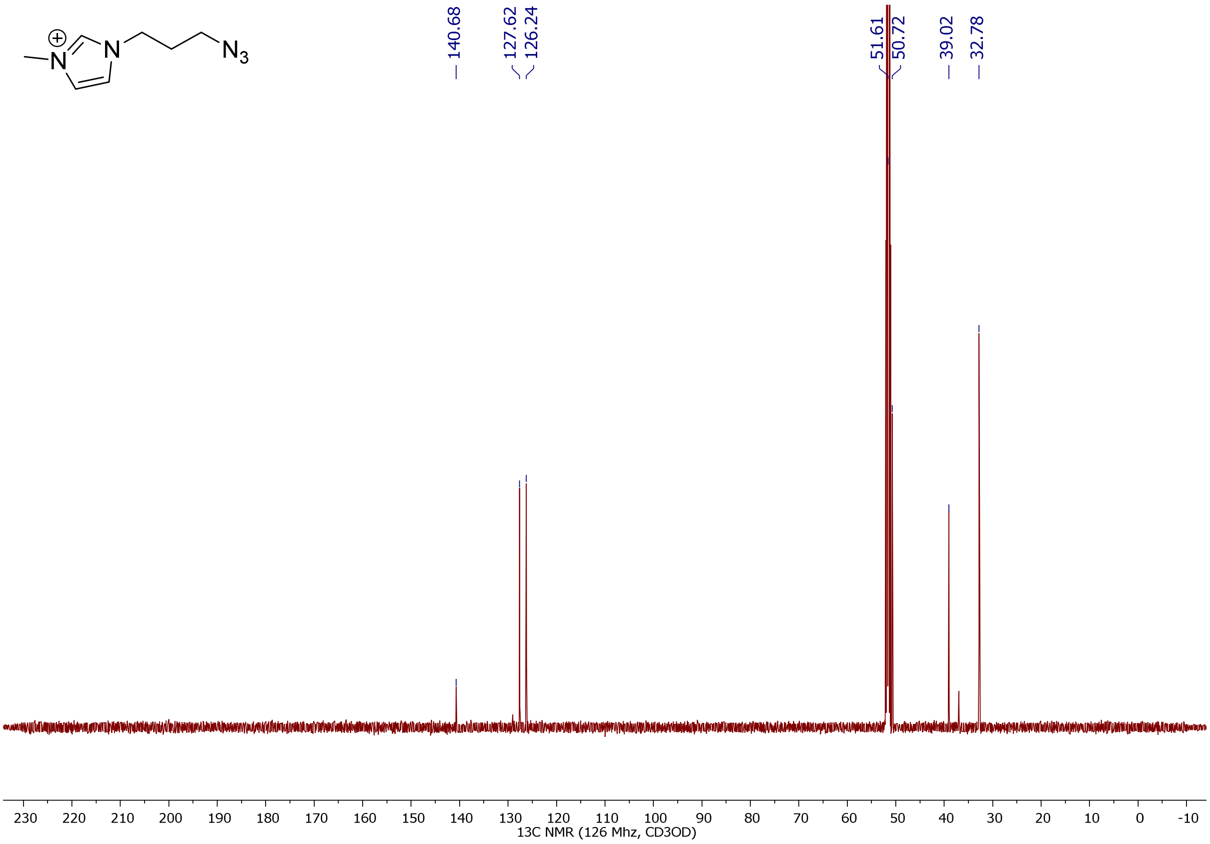
N3
140.68
127.62
126.24
51.61
50.72
39.02
32.78
13C NMR (126 Mhz, CD3OD)

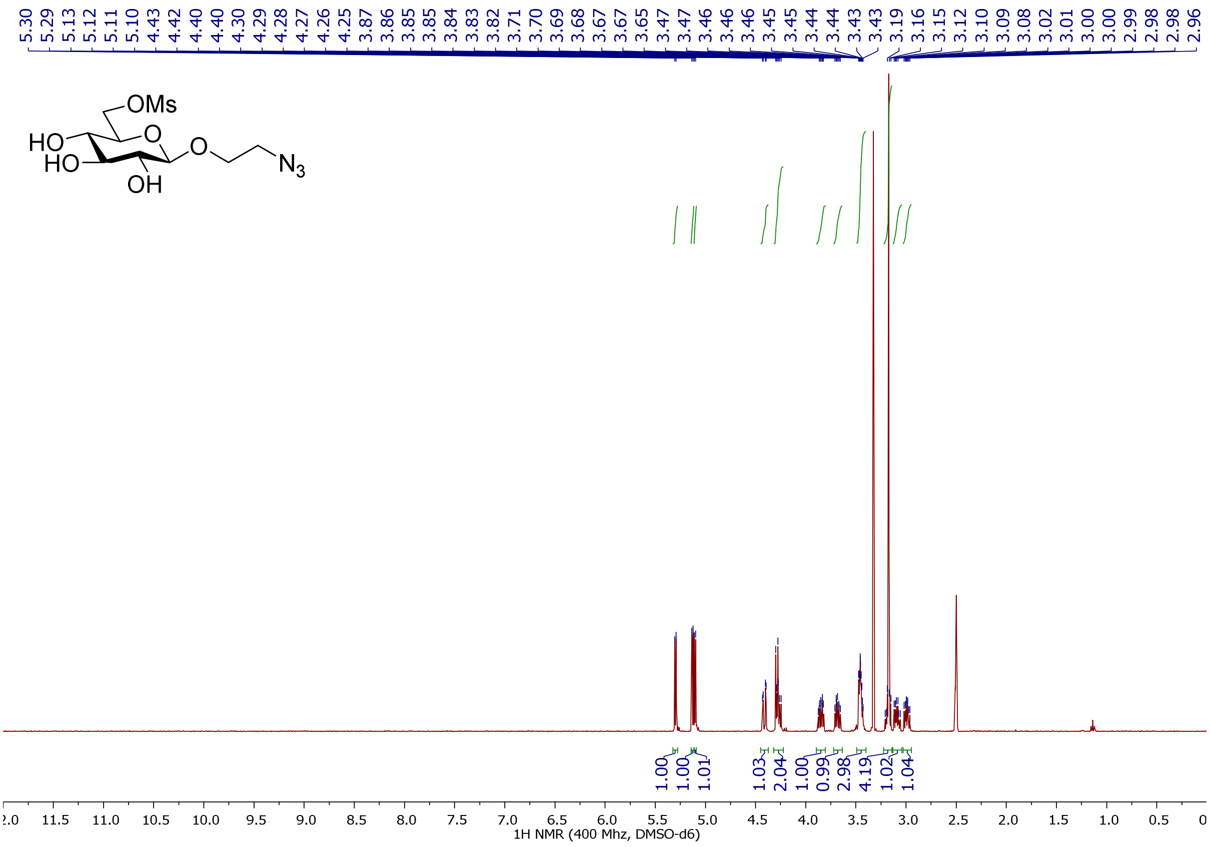
5.30
5.29
5.13
5.12
5.11
5.10
4.43
4.42
4.40
4.40
4.30
4.29
4.28
4.27
4.26
4.25
3.87
3.86
3.85
3.85
3.84
3.83
3.82
3.71
3.70
3.69
3.68
3.67
3.67
3.65
3.47
3.47
3.46
3.46
3.45
3.45
3.44
3.44
3.43
3.43
3.19
3.16
3.15
3.12
3.10
3.09
3.08
3.02
3.01
3.00
3.00
2.99
2.98
2.98
2.96
OMs
HO
HO
O
O
N3
OH
1.00
1.00
1.01
1.03
2.04
1.00
0.99
2.98
4.19
1.02
1.04
1H NMR (400 Mhz, DMSO-d6)

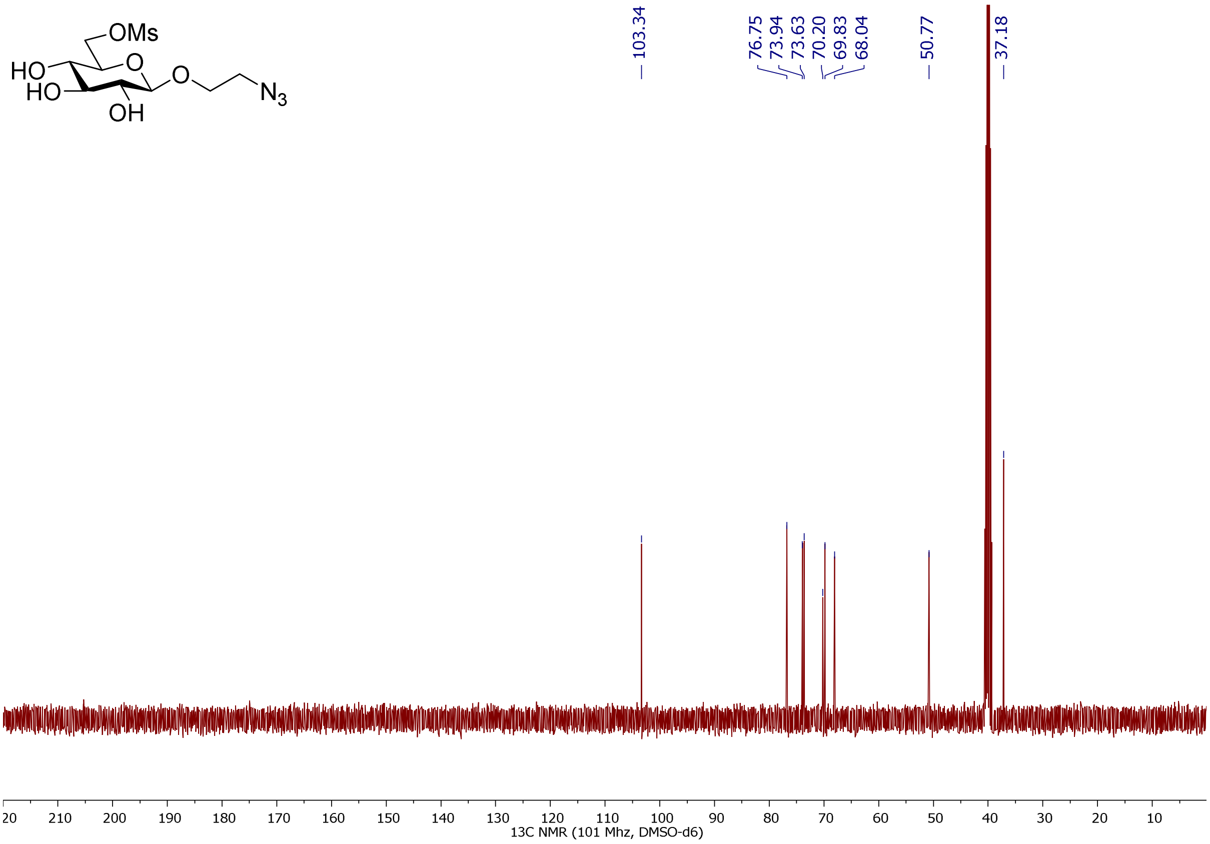
103.34
76.75
73.94
73.63
70.20
69.83
68.04
50.77
37.18
OMs
HO
HO
O
O
N3
OH
13C NMR (101 Mhz, DMSO-d6)

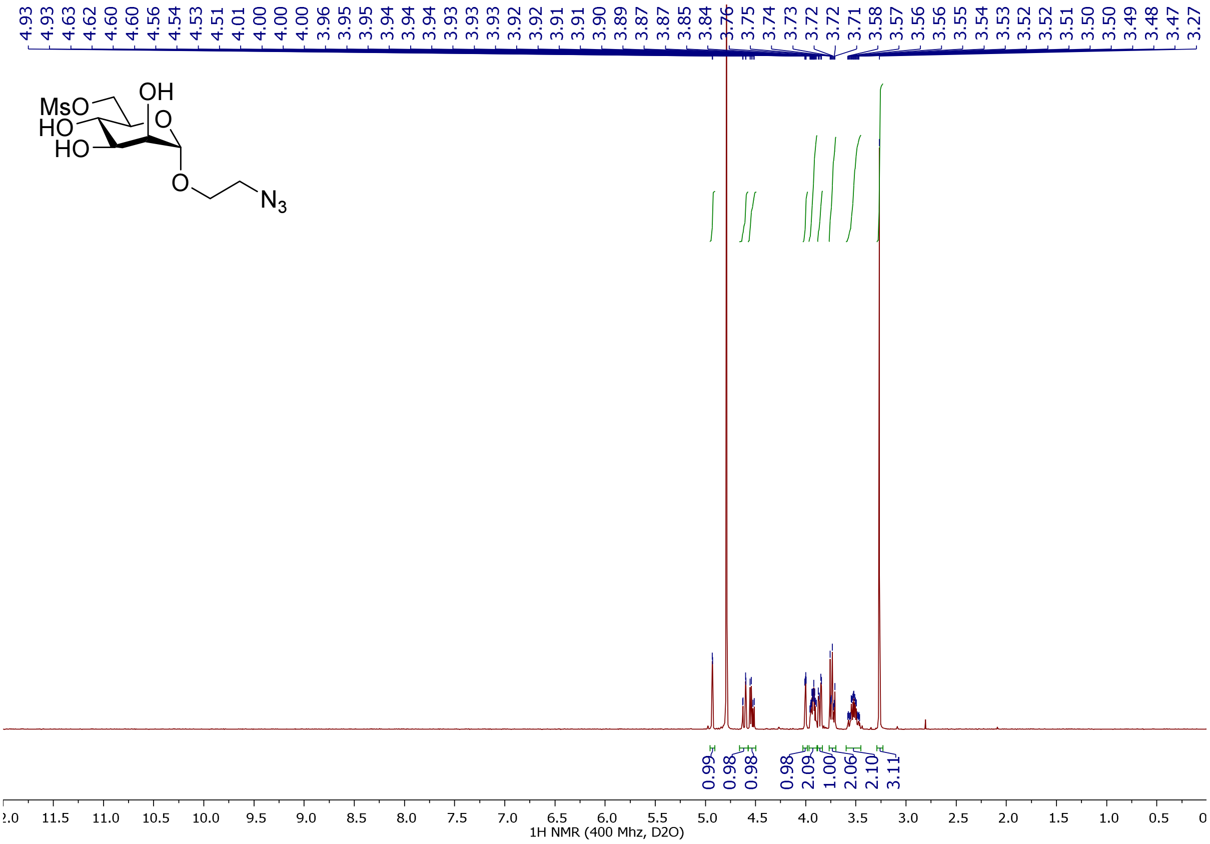
4.93 4.93 4.63 4.62 4.60 4.60 4.56 4.54 4.53 4.51 4.01 4.00 4.00 3.96 3.95 3.95 3.94 3.94 3.94 3.93 3.93 3.93 3.92 3.92 3.91 3.91 3.90 3.89 3.87 3.87 3.85 3.84 3.76 3.75 3.74 3.73 3.72 3.72 3.71 3.58 3.57 3.56 3.56 3.55 3.54 3.53 3.52 3.52 3.51 3.50 3.50 3.49 3.48 3.47 3.27
MsO
HO
HO
OH
O
O
N3
0.99
0.98
0.98
0.98
2.09
1.00
2.06
2.10
3.11
1H NMR (400 Mhz, D2O)

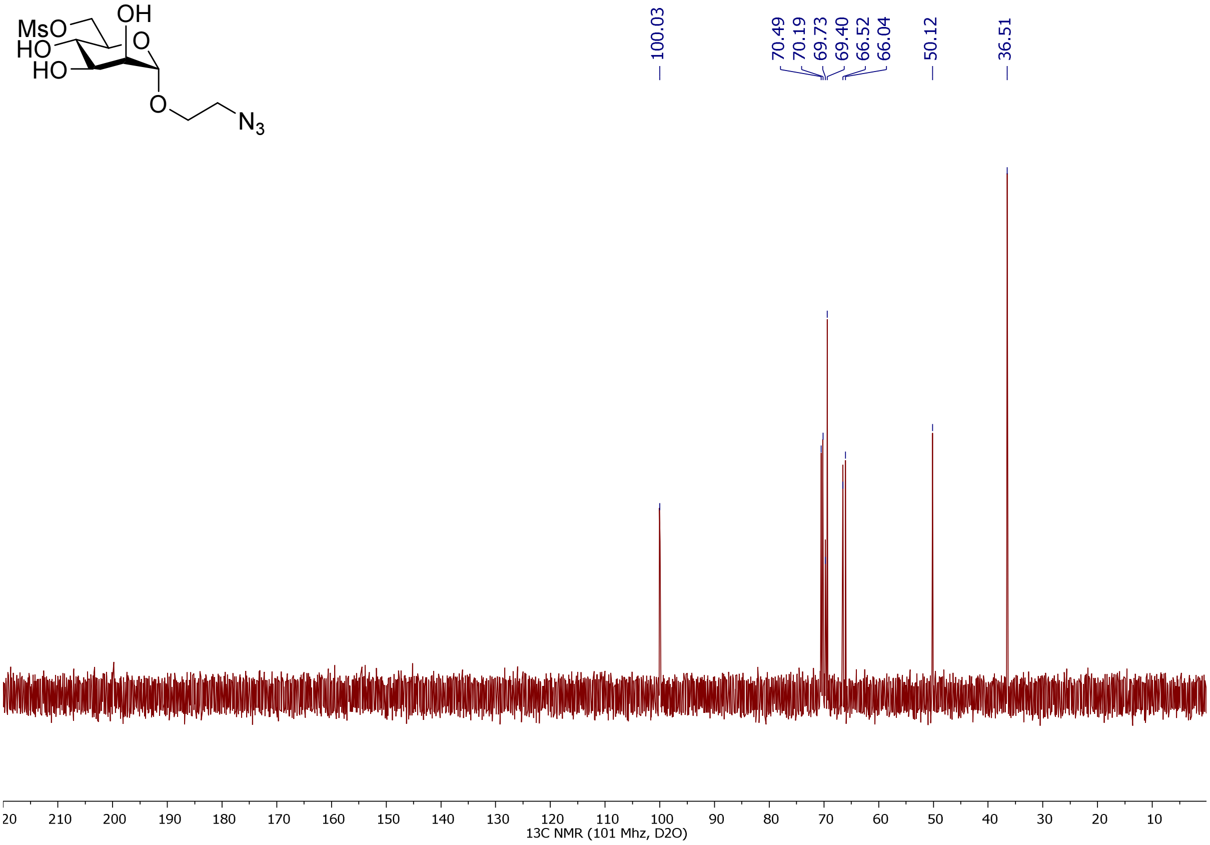
100.03
70.49
70.19
69.73
69.40
66.52
66.04
50.12
36.51
MsO
HO
HO
OH
O
O
N3
13C NMR (101 Mhz, D2O)

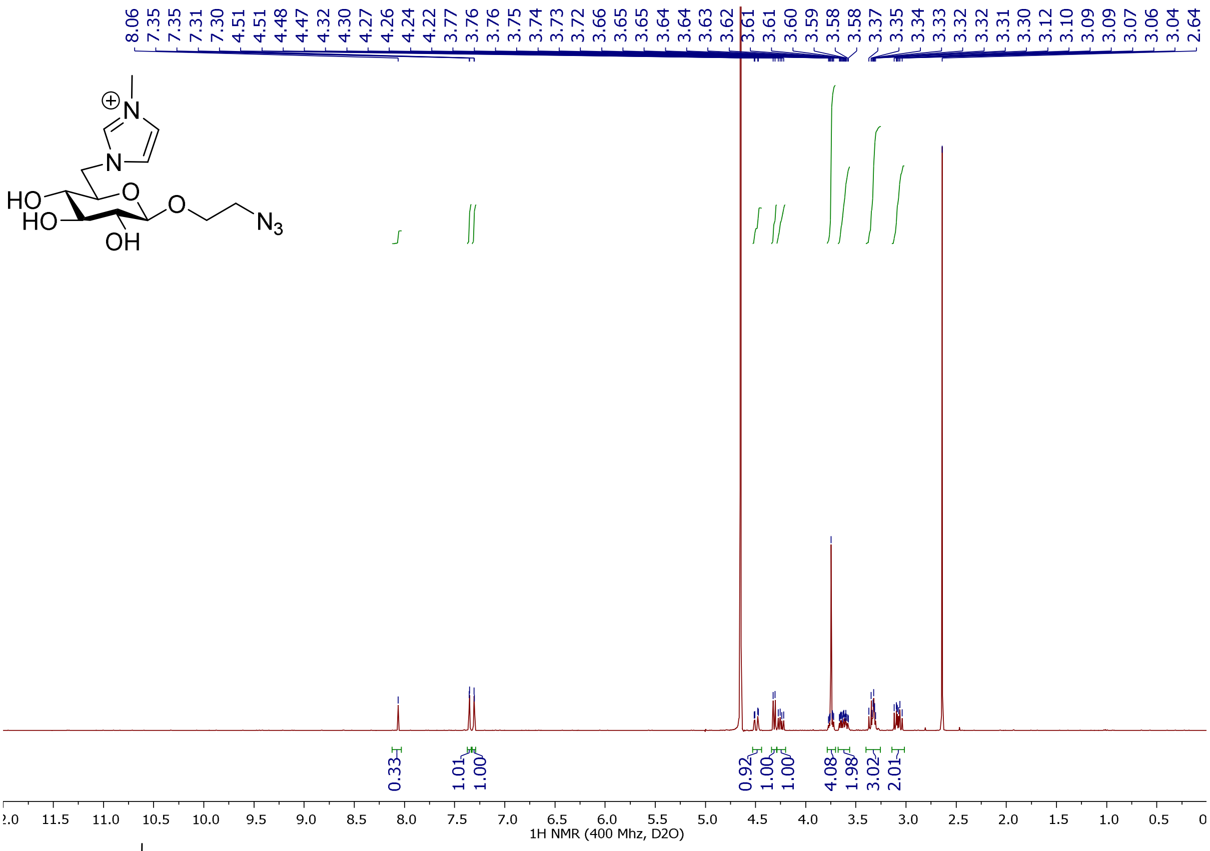
8.06 7.35 7.35 7.31 7.30 4.51 4.51 4.48 4.47 4.32 4.30 4.27 4.26 4.24 4.22 3.77 3.76 3.75 3.74 3.73 3.72 3.66 3.65 3.65 3.64 3.64 3.63 3.62 3.61 3.61 3.60 3.59 3.58 3.58 3.37 3.35 3.34 3.33 3.32 3.32 3.31 3.30 3.12 3.10 3.09 3.09 3.07 3.06 3.04 2.64
HO
HO
OH
O
O
N
N
N3
0.33
1.01
1.00
0.92
1.00
1.00
4.08
1.98
3.02
2.01
1H NMR (400 Mhz, D2O)

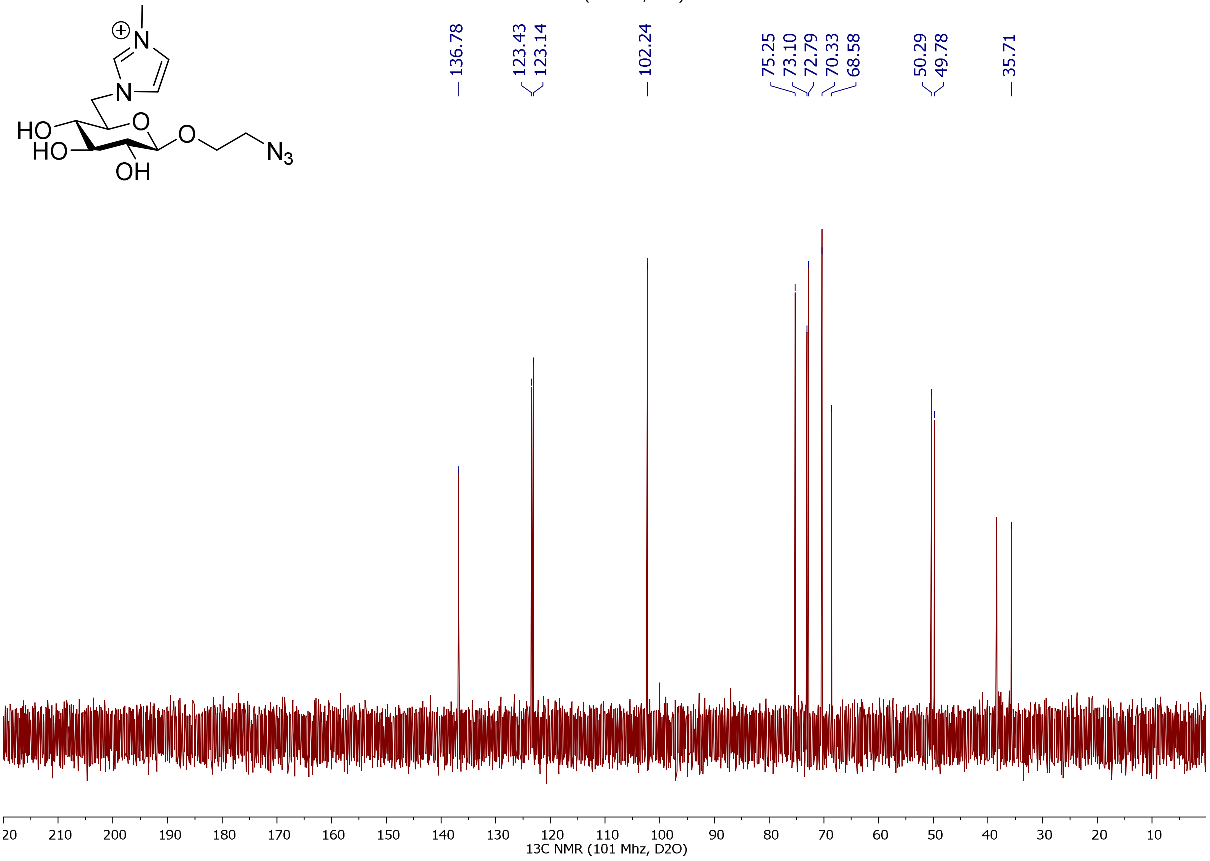
136.78
123.43
123.14
102.24
75.25
73.10
72.79
70.33
68.58
50.29
49.78
35.71
HO
HO
OH
O
O
N
N
N3
13C NMR (101 Mhz, D2O)

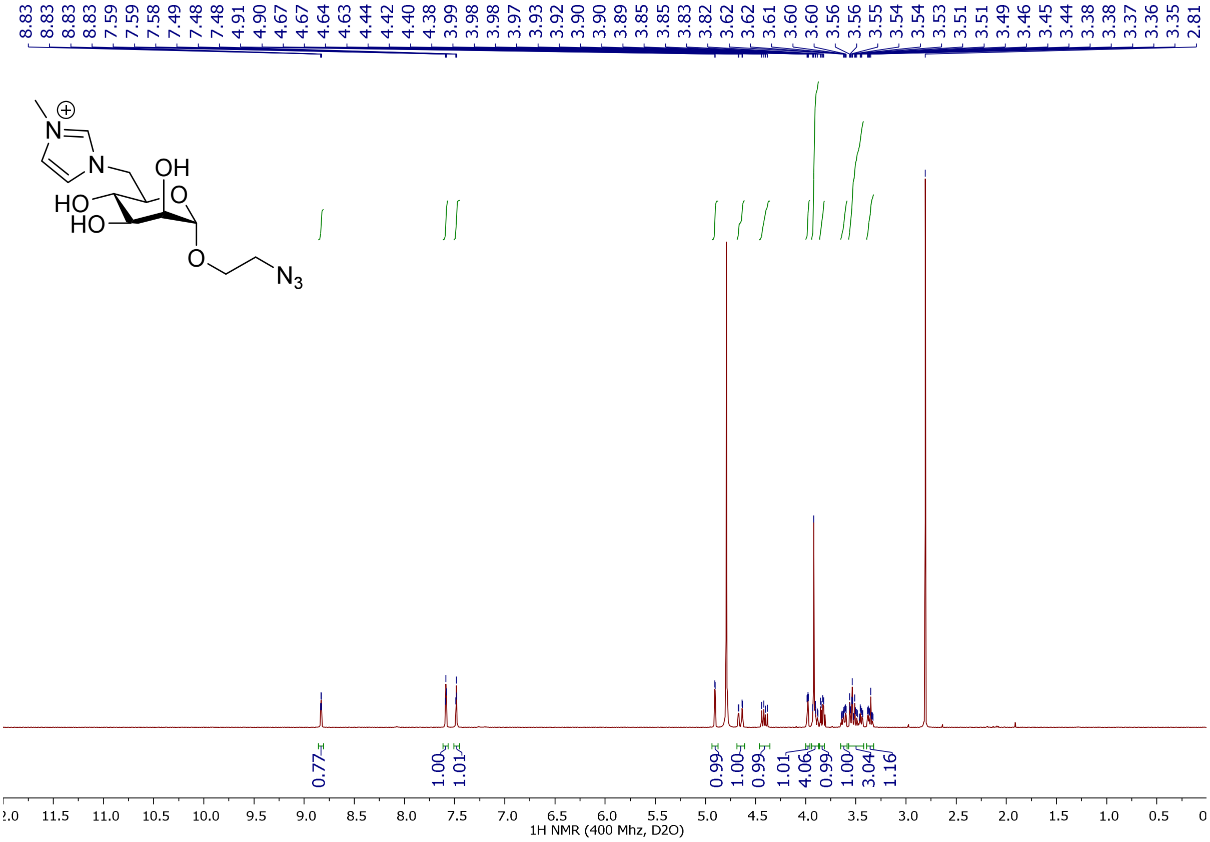
8.83 8.83 8.83 8.83 7.59 7.59 7.58 7.49 7.48 7.48 4.91 4.90 4.67 4.67 4.64 4.63 4.44 4.42 4.40 4.38 3.99 3.98 3.98 3.97 3.93 3.92 3.90 3.89 3.85 3.85 3.83 3.82 3.62 3.62 3.61 3.60 3.60 3.56 3.56 3.55 3.54 3.54 3.53 3.51 3.51 3.49 3.46 3.45 3.44 3.38 3.38 3.37 3.36 3.35 2.81
OH
O
HO
HO
O
N
N
N_3
0.77
1.00
1.01
0.99
1.00
0.99
1.01
4.06
0.99
1.00
3.04
1.16
1H NMR (400 Mhz, D2O)

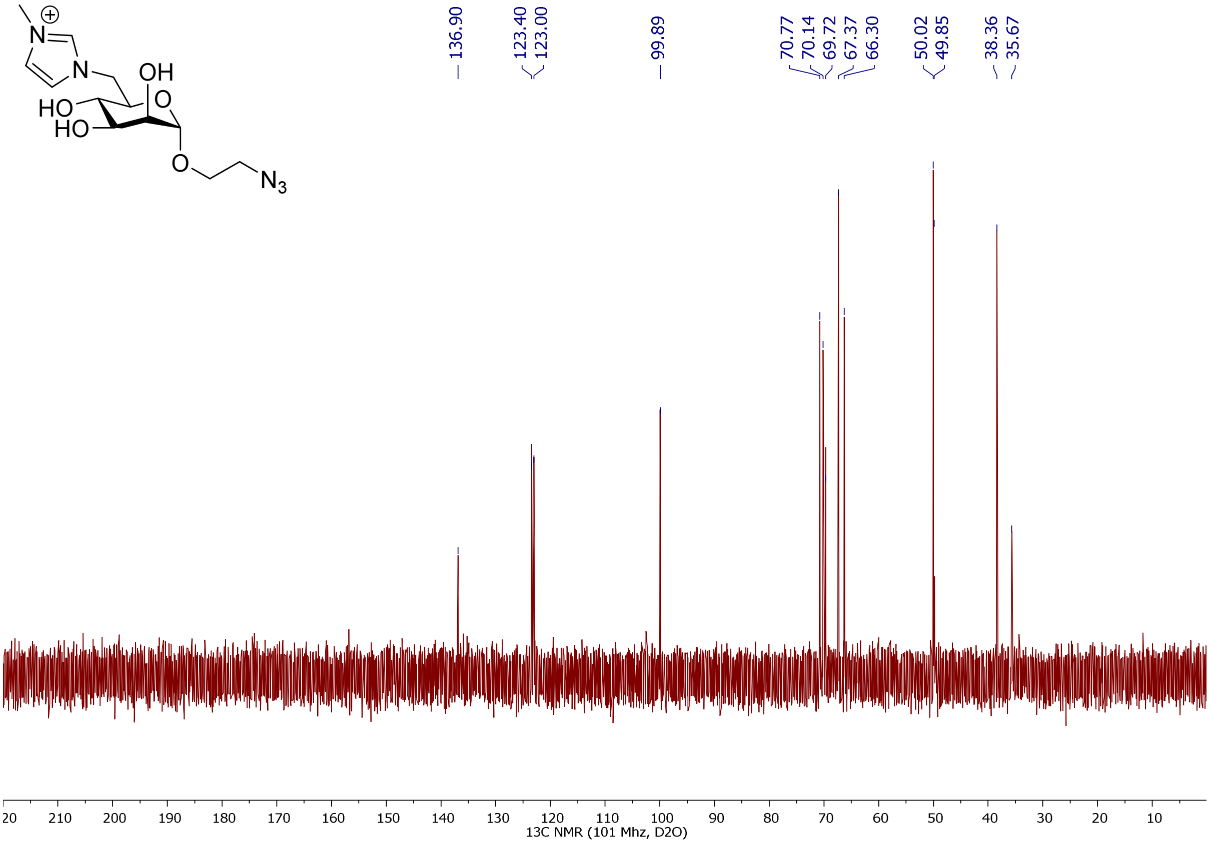
136.90
123.40 123.00
99.89
70.77 70.14 69.72 67.37 66.30
50.02 49.85
38.36 35.67
OH
O
HO
HO
O
N
N
N_3
13C NMR (101 Mhz, D2O)

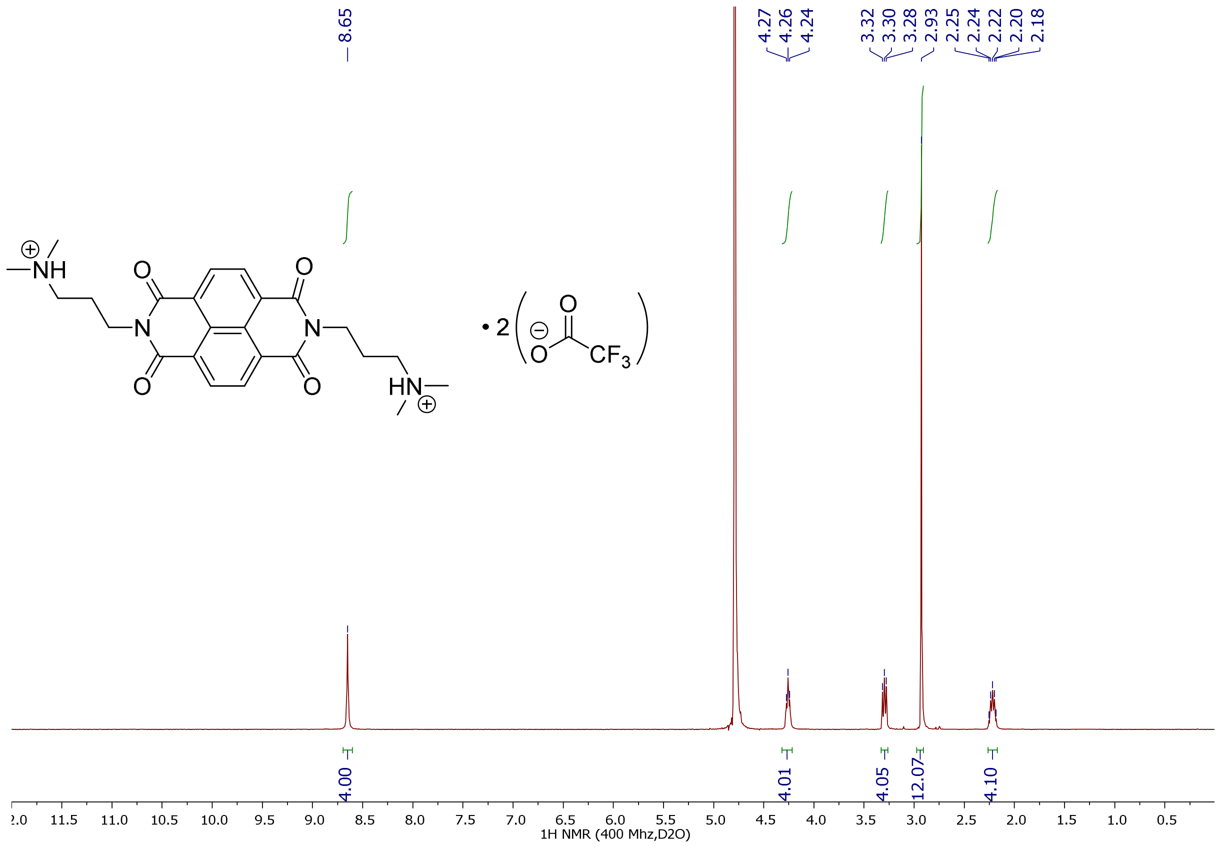

8.65
4.27
4.26
4.24
3.32
3.30
3.28
2.93
2.25
2.24
2.22
2.20
2.18
4.00
4.01
4.05
12.07
4.10
1H NMR (400 Mhz,D2O)

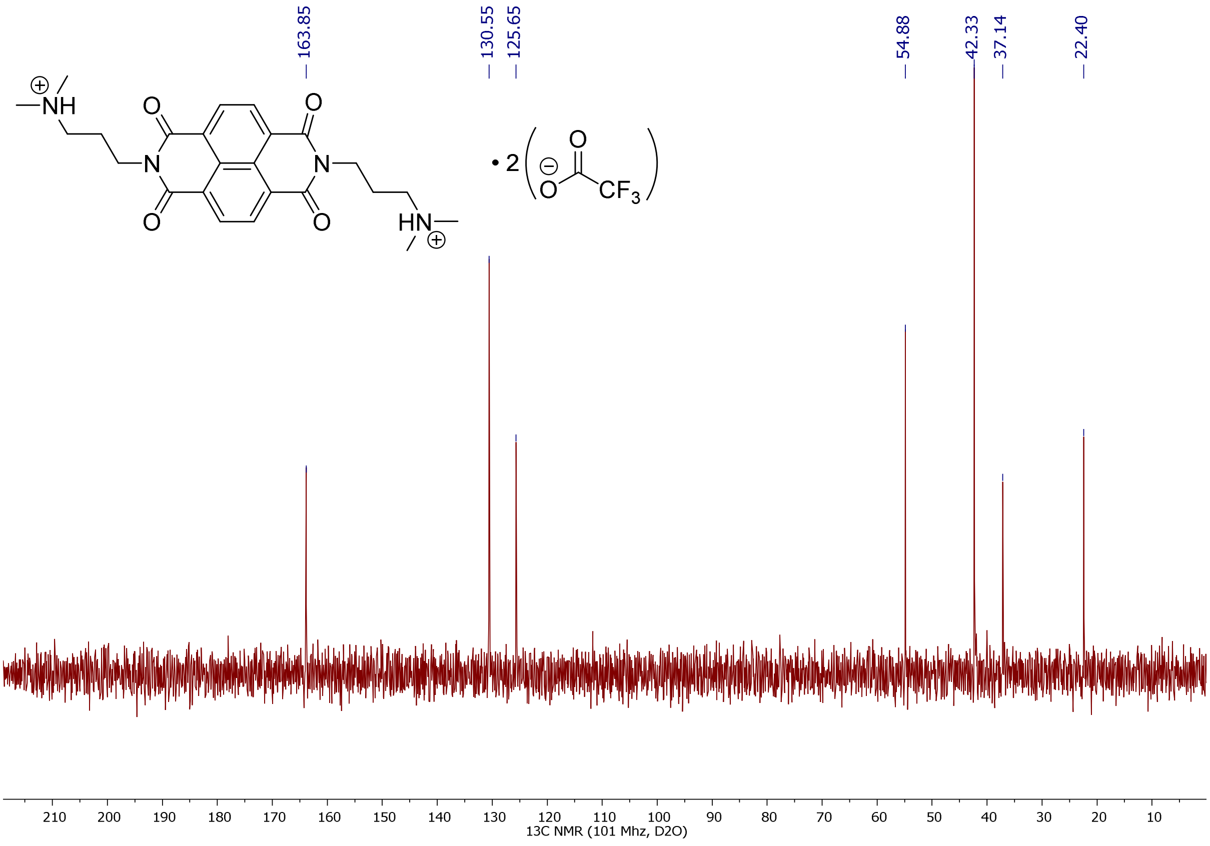

163.85
130.55
125.65
54.88
42.33
37.14
22.40
13C NMR (101 Mhz, D2O)

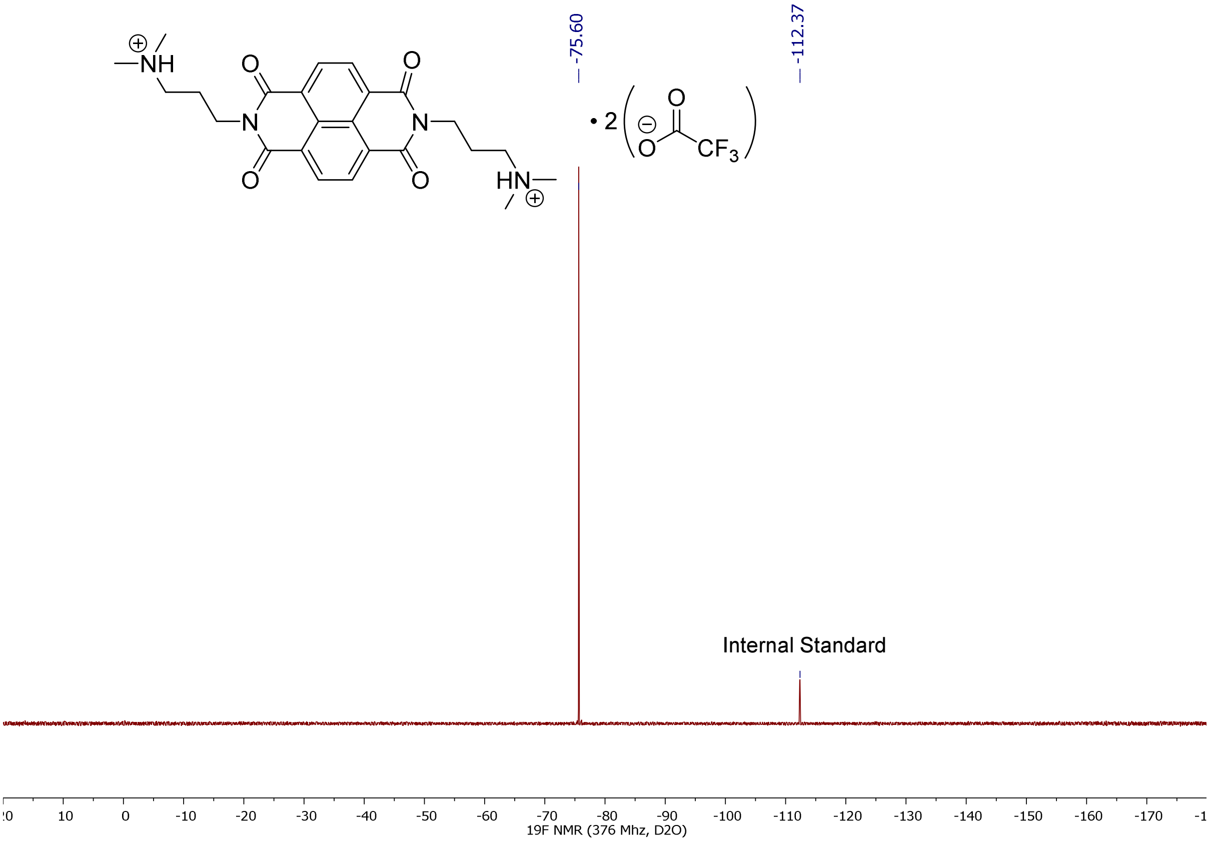
-75.60
-112.37
Internal Standard
• 2
19F NMR (376 Mhz, D2O)

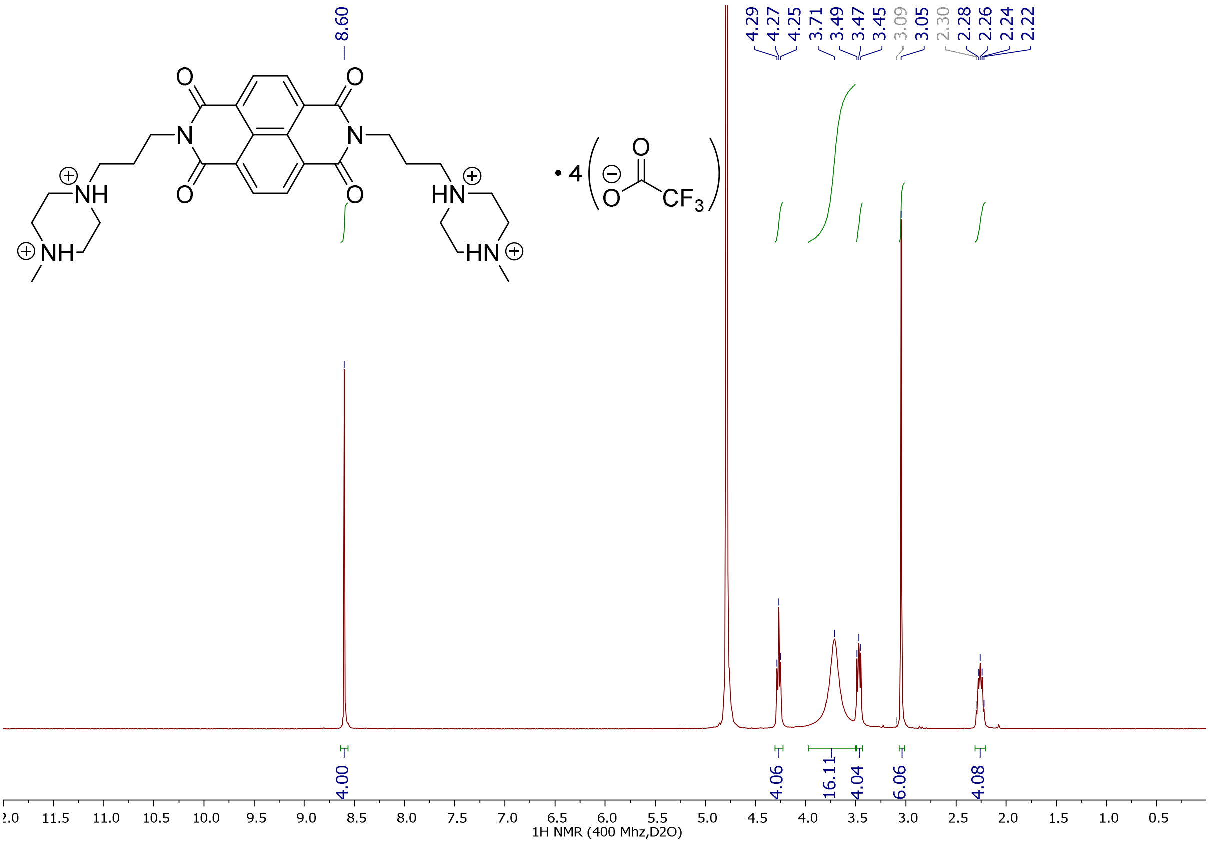
8.60
4.29
4.27
4.25
3.71
3.49
3.47
3.45
3.09
3.05
2.30
2.28
2.26
2.24
2.22
• 4
4.00
4.06
16.11
4.04
6.06
4.08
1H NMR (400 Mhz,D2O)

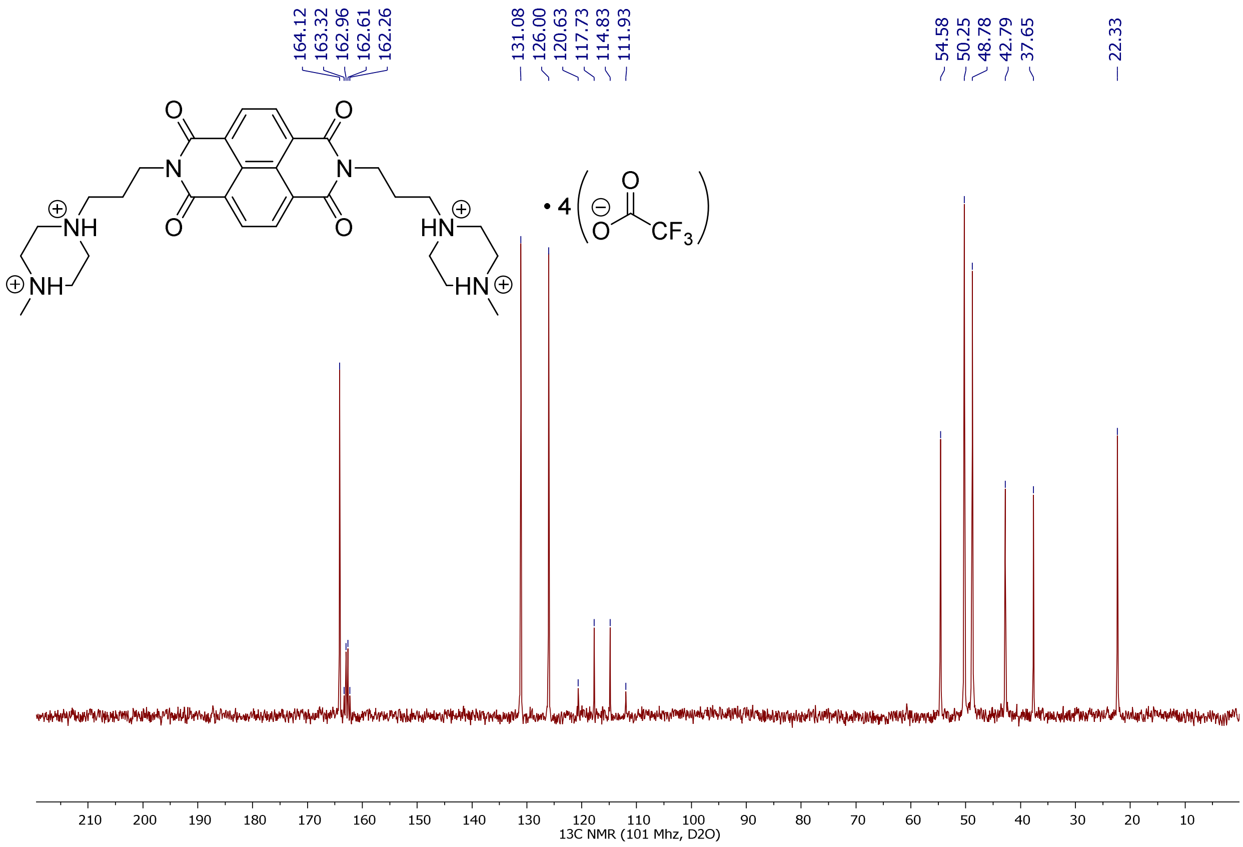
164.12
163.32
162.96
162.61
162.26
131.08
126.00
120.63
117.73
114.83
111.93
54.58
50.25
48.78
42.79
37.65
22.33
• 4
CF3
210 200 190 180 170 160 150 140 130 120 110 100 90 80 70 60 50 40 30 20 10
13C NMR (101 Mhz, D2O)

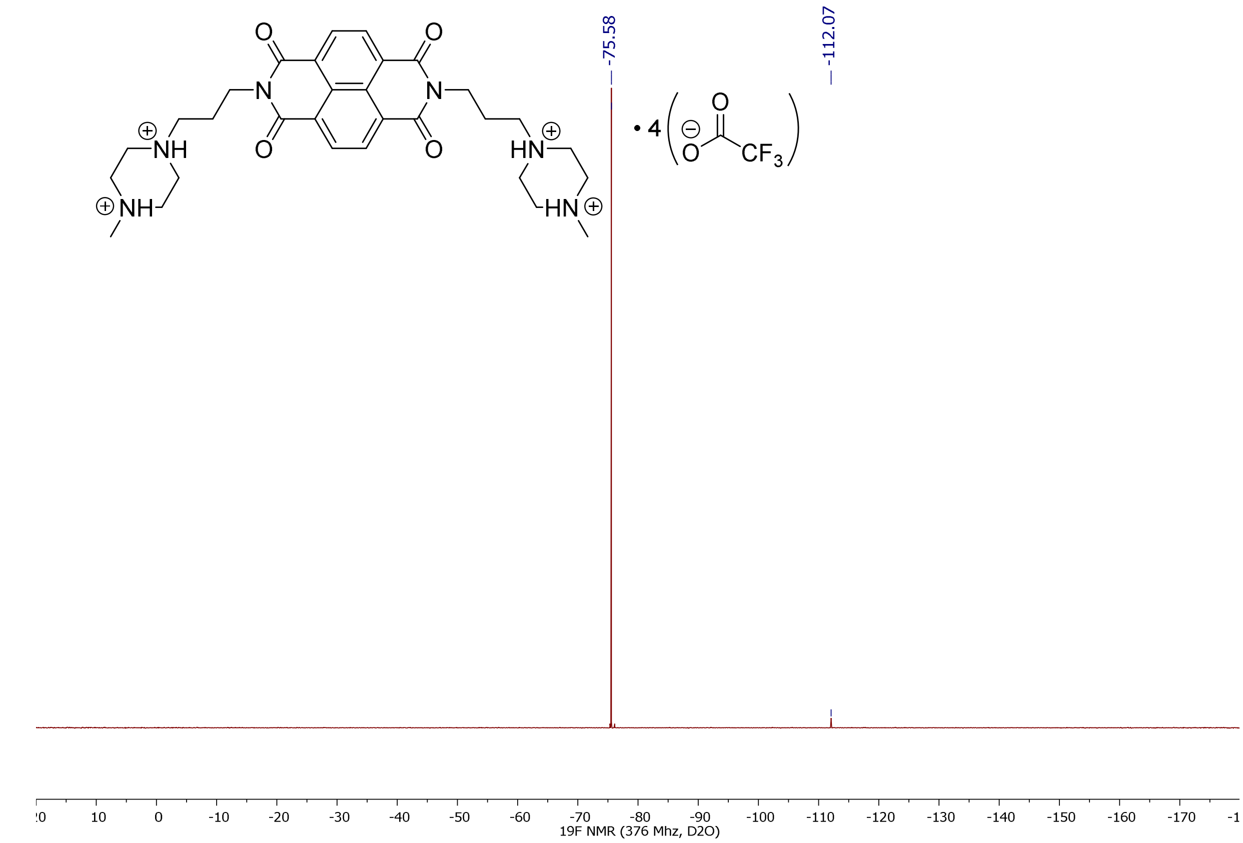
-75.58
-112.07
• 4
CF3
10 0 -10 -20 -30 -40 -50 -60 -70 -80 -90 -100 -110 -120 -130 -140 -150 -160 -170
19F NMR (376 Mhz, D2O)

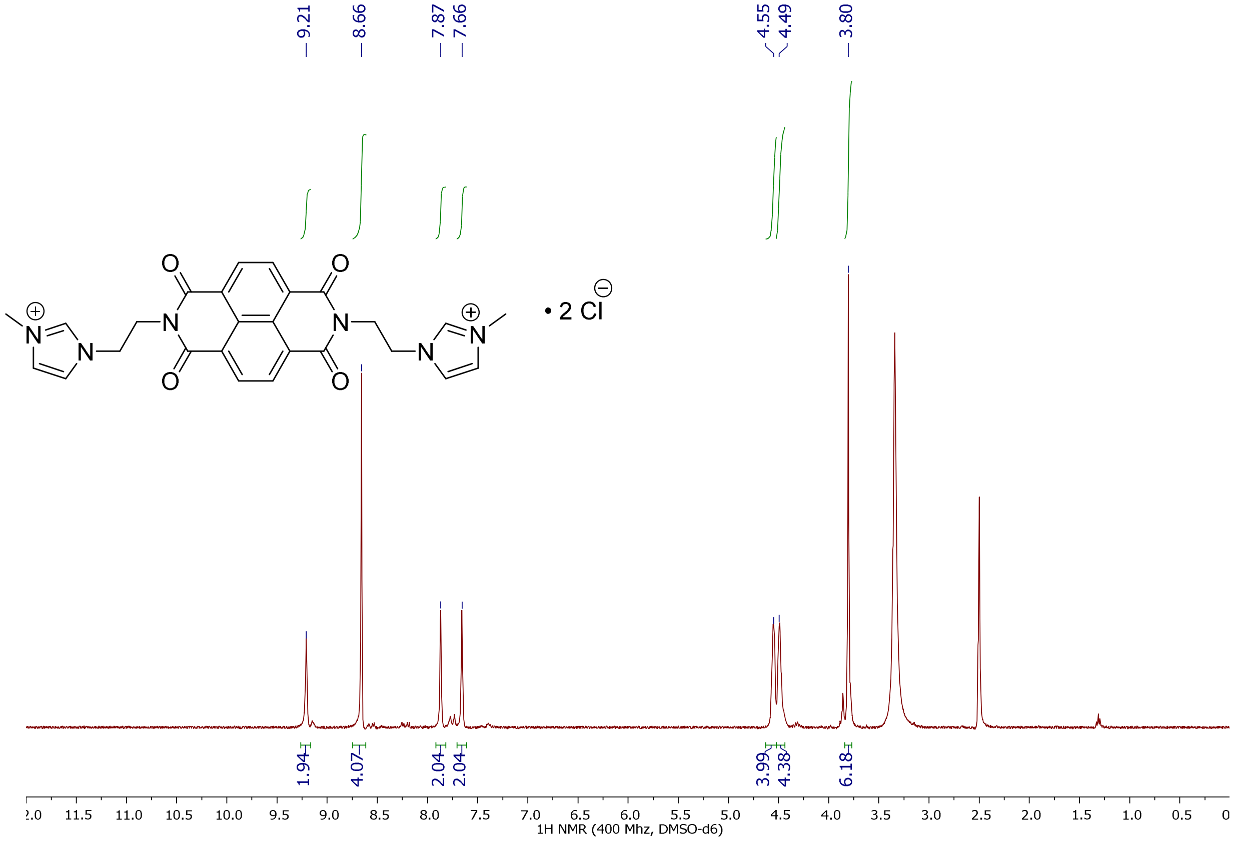
9.21
8.66
7.87
7.66
4.55
4.49
3.80
• 2 Cl
1.94
4.07
2.04
2.04
3.99
4.38
6.18
1H NMR (400 Mhz, DMSO-d6)

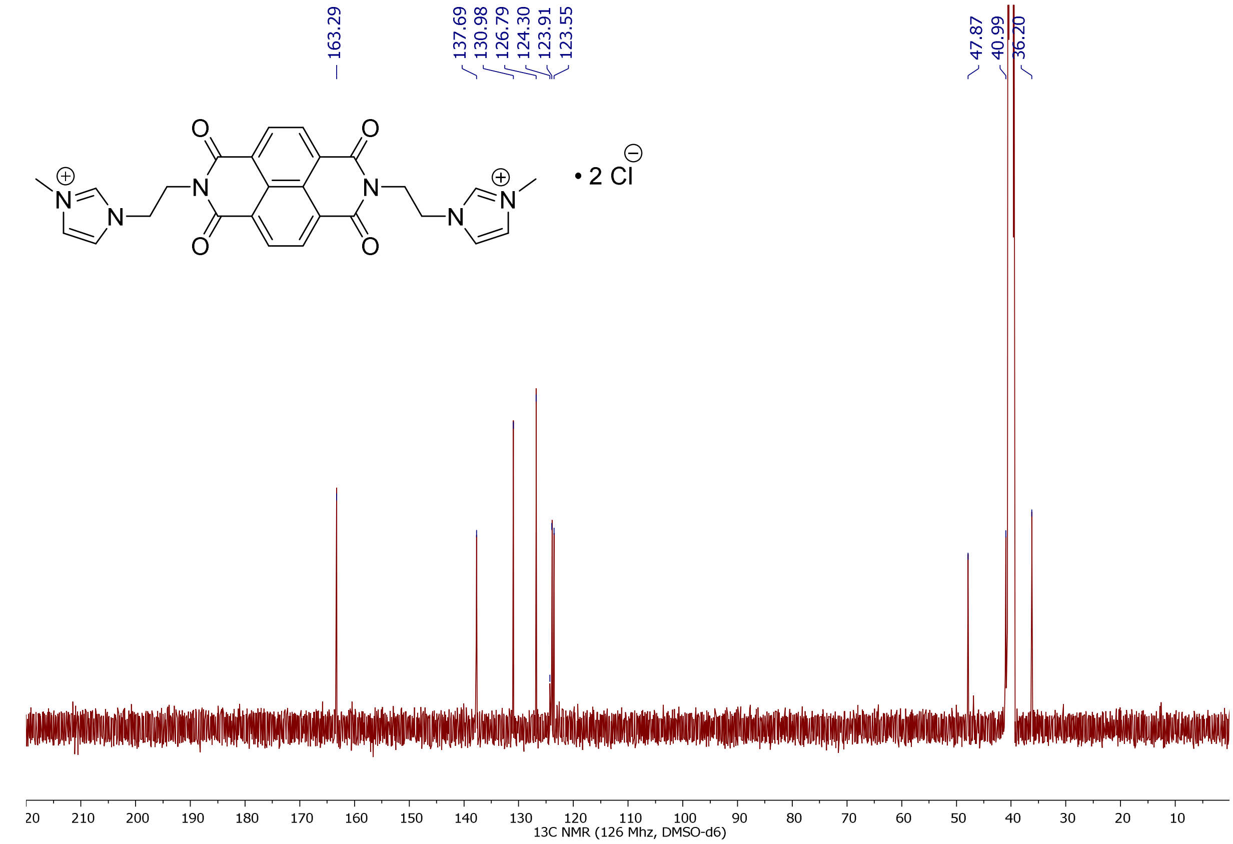
163.29
137.69
130.98
126.79
124.30
123.91
123.55
47.87
40.99
36.20
• 2 Cl
13C NMR (126 Mhz, DMSO-d6)

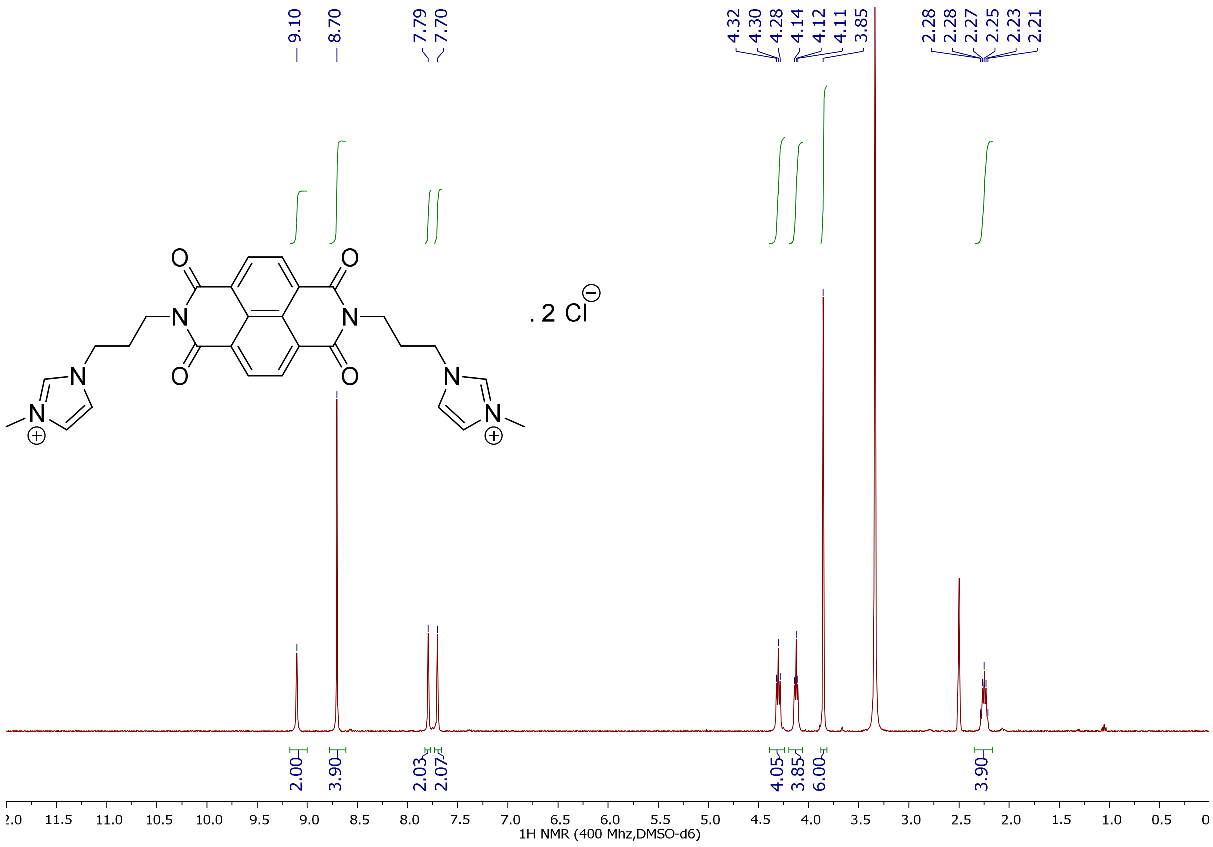
9.10
8.70
7.79
7.70
4.32
4.30
4.28
4.14
4.12
4.11
3.85
2.28
2.28
2.27
2.25
2.23
2.21
. 2 Cl
2.00
3.90
2.03
2.07
4.05
3.85
6.00
3.90
1H NMR (400 Mhz,DMSO-d6)

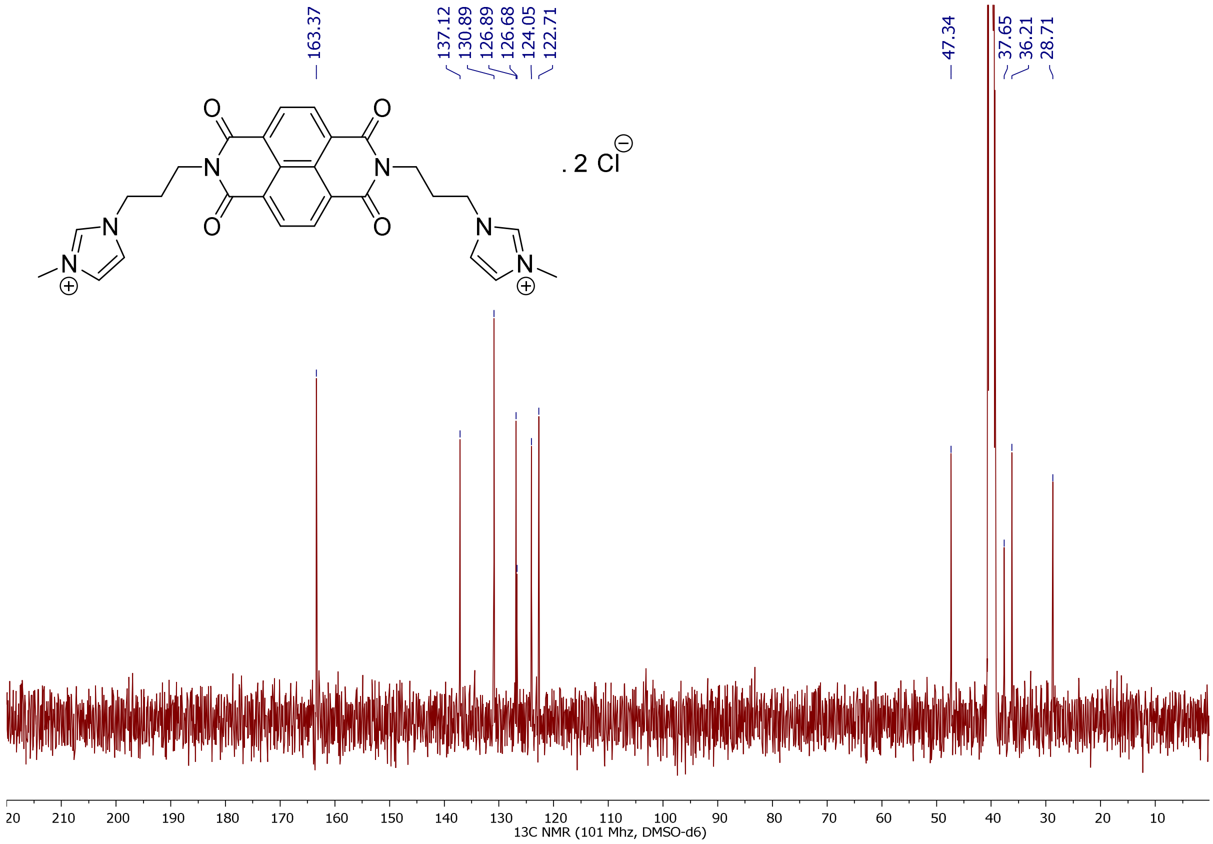
163.37
137.12
130.89
126.89
126.68
124.05
122.71
47.34
37.65
36.21
28.71
. 2 Cl
13C NMR (101 Mhz, DMSO-d6)

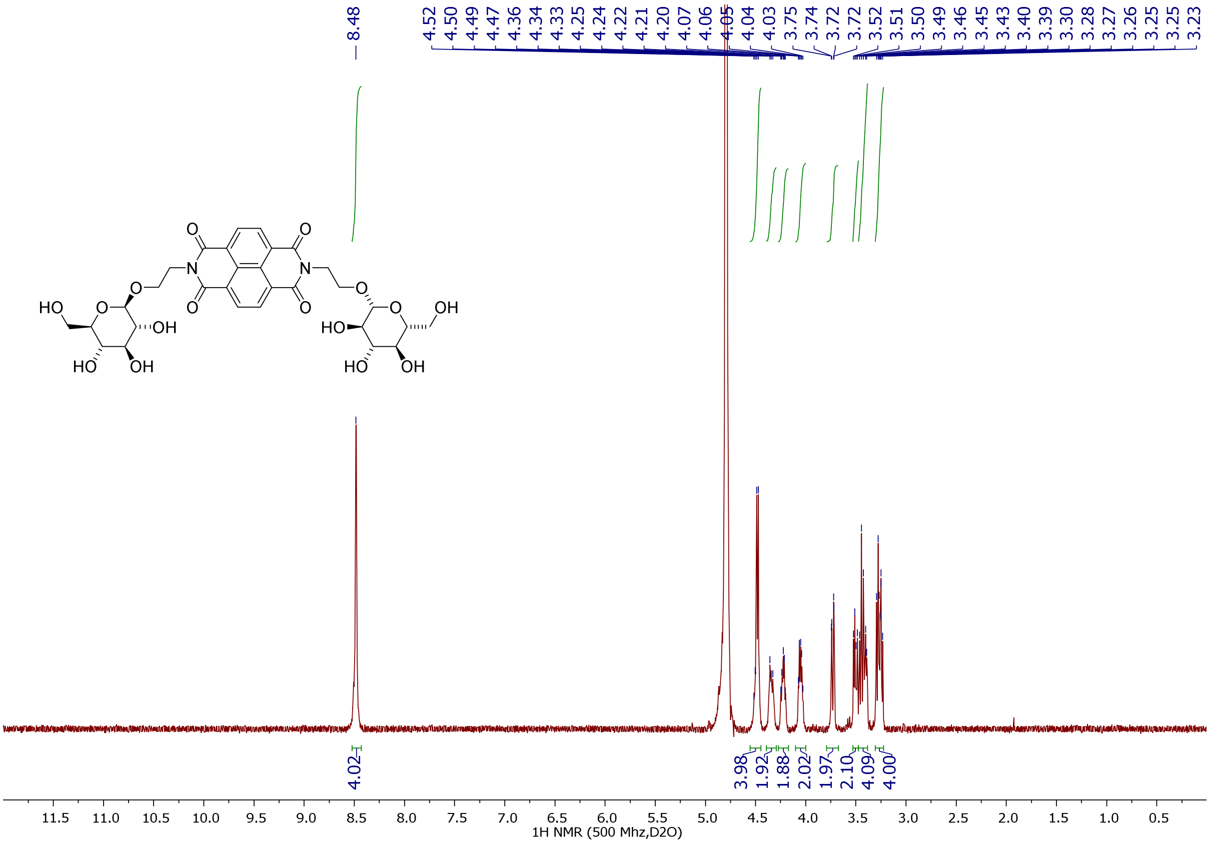

8.48
4.52 4.50 4.49 4.47 4.36 4.34 4.33 4.25 4.24 4.22 4.21 4.20 4.07 4.06 4.05 4.04 4.03 3.75 3.74 3.72 3.72 3.52 3.51 3.50 3.49 3.46 3.45 3.43 3.40 3.39 3.30 3.28 3.27 3.26 3.25 3.23
HO
O
O
OH
HO
OH
N
4.02
3.98 1.92 1.88 2.02 1.97 2.10 4.09 4.00
11.5 11.0 10.5 10.0 9.5 9.0 8.5 8.0 7.5 7.0 6.5 6.0 5.5 5.0 4.5 4.0 3.5 3.0 2.5 2.0 1.5 1.0 0.5
1H NMR (500 Mhz,D2O)

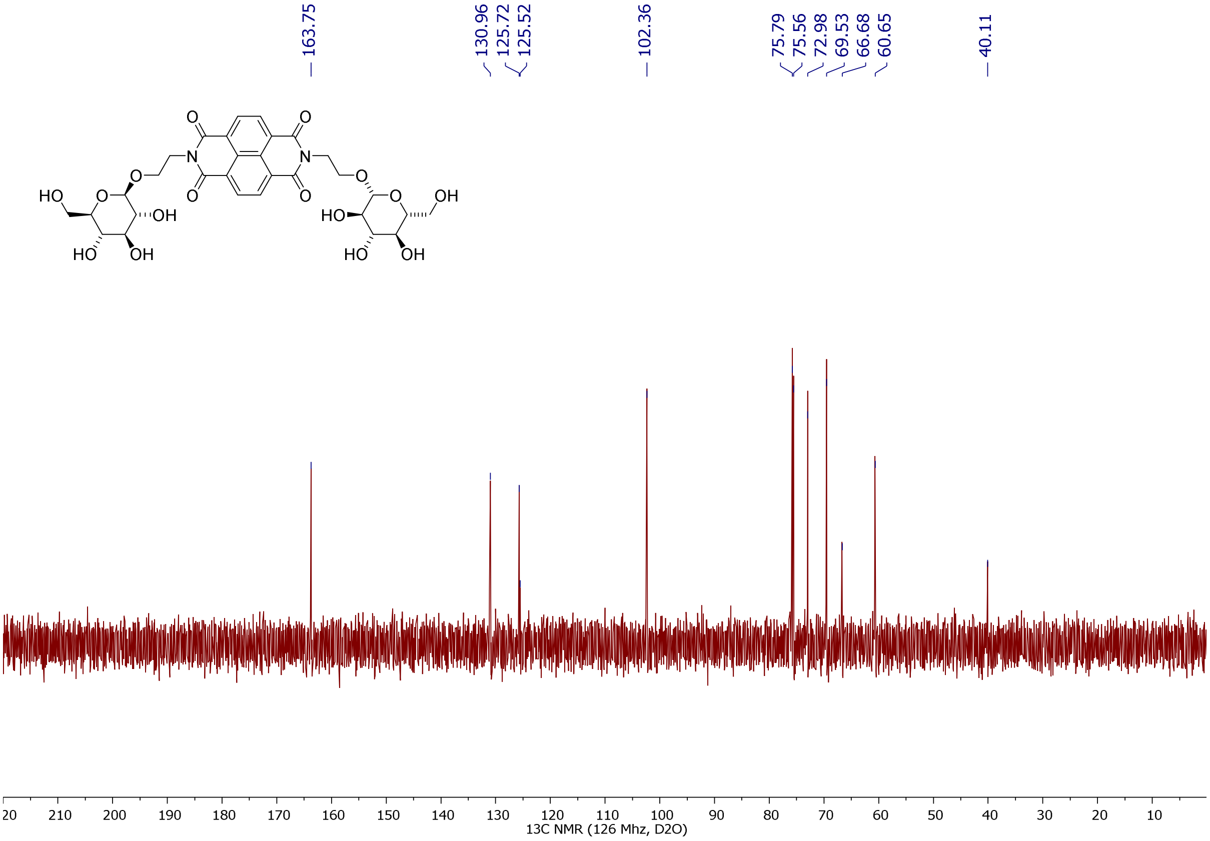

163.75
130.96 125.72 125.52
102.36
75.79 75.56 72.98 69.53 66.68 60.65
40.11
HO
O
O
OH
HO
OH
N
20 210 200 190 180 170 160 150 140 130 120 110 100 90 80 70 60 50 40 30 20 10
13C NMR (126 Mhz, D2O)

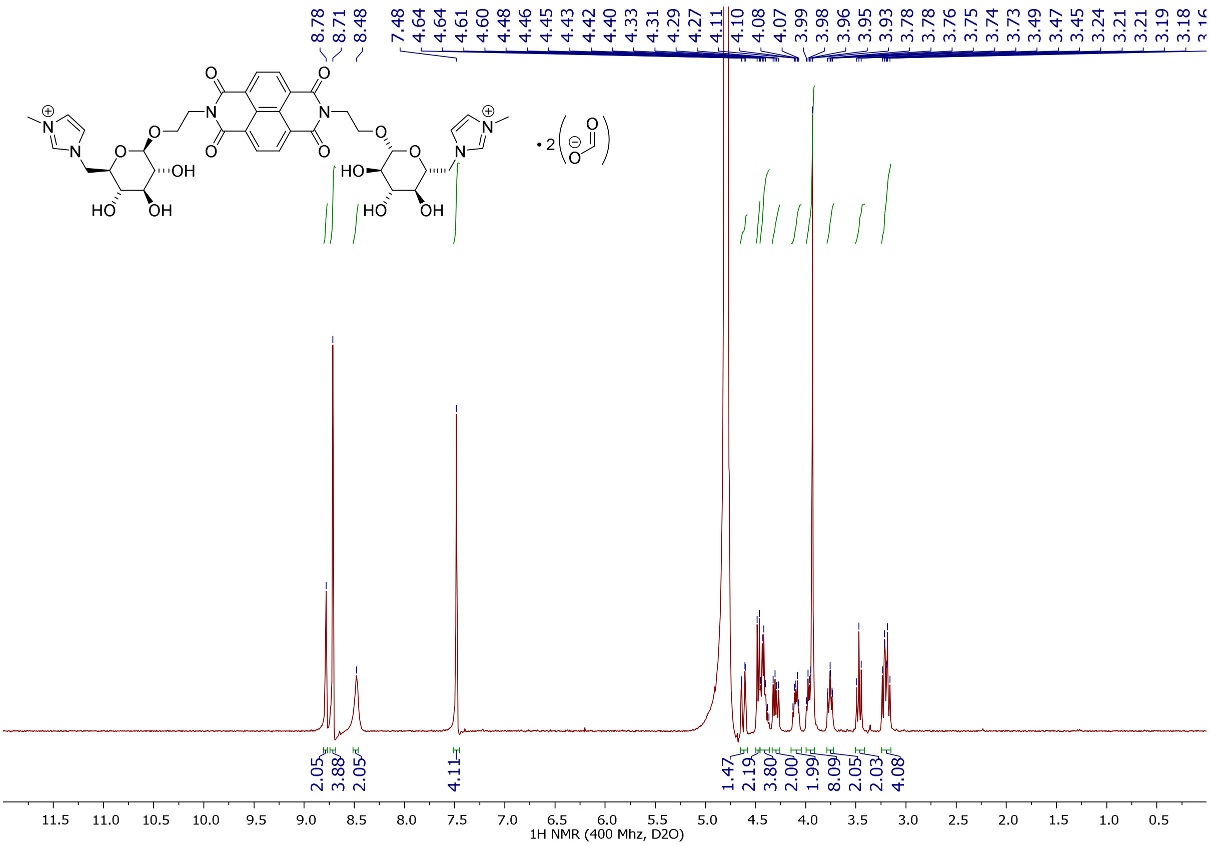
8.78
8.71
8.48
7.48
4.64
4.64
4.61
4.60
4.48
4.46
4.45
4.43
4.42
4.40
4.33
4.31
4.29
4.27
4.11
4.10
4.08
4.07
3.99
3.98
3.96
3.95
3.93
3.78
3.76
3.75
3.74
3.73
3.49
3.47
3.45
3.24
3.21
3.21
3.19
3.18
2.05
3.88
2.05
4.11
1.47
2.19
3.80
2.00
1.99
8.09
2.05
2.03
4.08
11.5 11.0 10.5 10.0 9.5 9.0 8.5 8.0 7.5 7.0 6.5 6.0 5.5 5.0 4.5 4.0 3.5 3.0 2.5 2.0 1.5 1.0 0.5
1H NMR (400 Mhz, D2O)

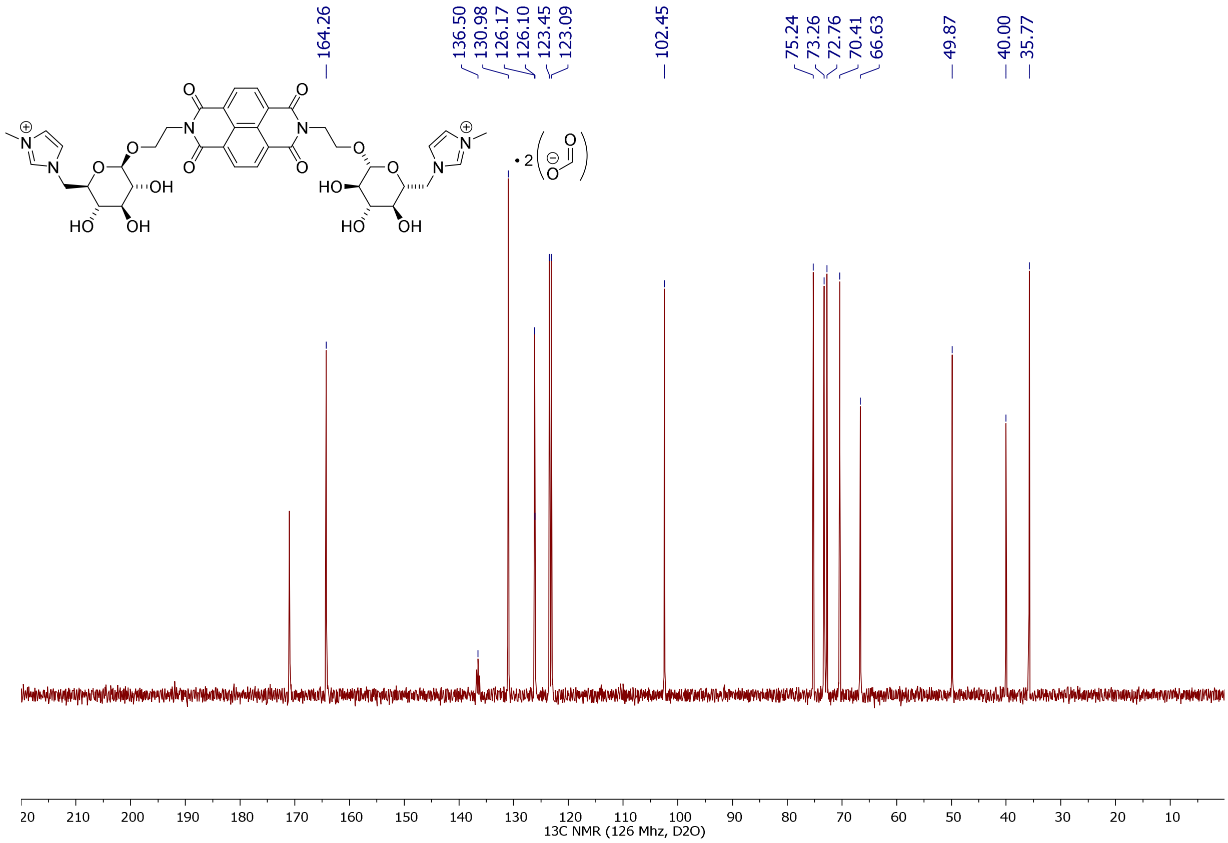
164.26
136.50
130.98
126.17
126.10
123.45
123.09
102.45
75.24
73.26
72.76
70.41
66.63
49.87
40.00
35.77
20 210 200 190 180 170 160 150 140 130 120 110 100 90 80 70 60 50 40 30 20 10
13C NMR (126 Mhz, D2O)

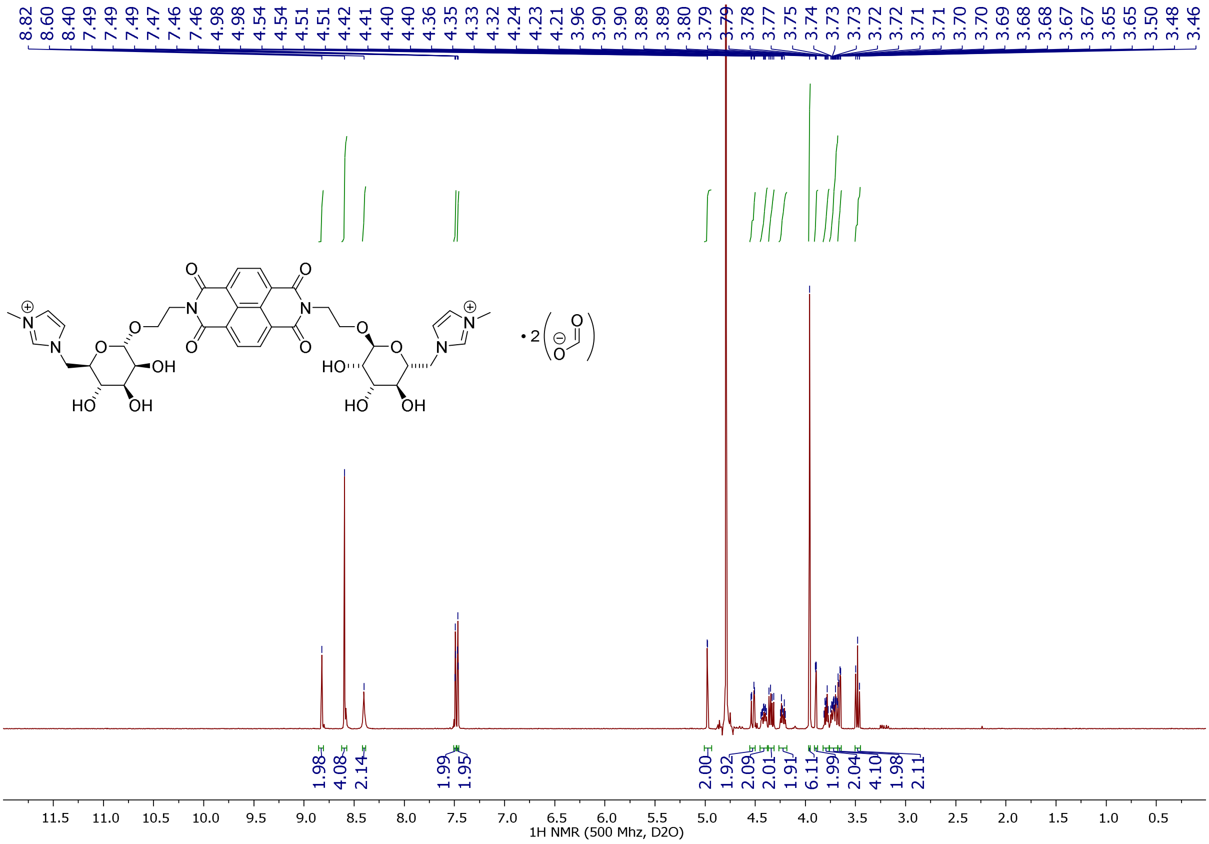
1.98
4.08
2.14
1.99
1.95
2.00
1.92
2.09
2.01
1.91
6.11
1.99
2.04
4.10
1.98
2.11
1H NMR (500 Mhz, D2O)

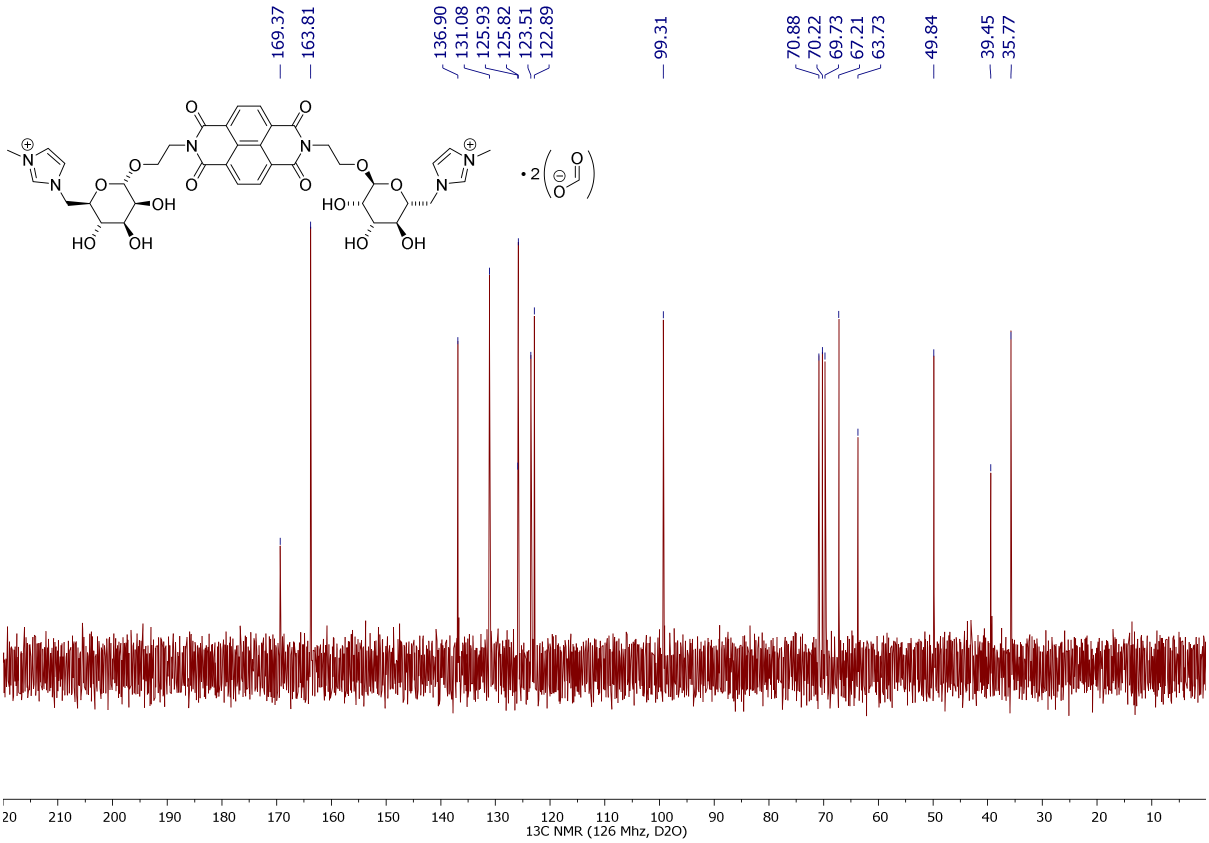
169.37
163.81
136.90
131.08
125.93
125.82
123.51
122.89
99.31
70.88
70.22
69.73
67.21
63.73
49.84
39.45
35.77
13C NMR (126 Mhz, D2O)

References

(1) Pangborn, A. B.; Giardello, M. A.; Grubbs, R. H.; Rosen, R. K.; Timmers, F. J. *Organometallics* **1996**, *15* (5), 1518.

(2) Still, W. C.; Kahn, M.; Mitra, A. *J. Org. Chem.* **1978**, *43* (14), 2923.

(3) De Cian, A.; Guittat, L.; Kaiser, M.; Saccà, B.; Amrane, S.; Bourdoncle, A.; Alberti, P.; Teulade-Fichou, M.-P.; Lacroix, L.; Mergny, J.-L. *Methods* **2007**, *42* (2), 183.

(4) Brautigam, C. A.; Zhao, H.; Vargas, C.; Keller, S.; Schuck, P. *Nat. Protoc.* **2016**, *11* (5), 882.

(5) Keller, S.; Vargas, C.; Zhao, H.; Piszczek, G.; Brautigam, C. A.; Schuck, P. *Anal. Chem.* **2012**, *84* (11), 5066.

(6) Houtman, J. C. D.; Brown, P. H.; Bowden, B.; Yamaguchi, H.; Appella, E.; Samelson, L. E.; Schuck, P. *Protein Sci.* **2007**, *16* (1), 30.

(7) Brautigam, C. A. *Calculations and Publication-Quality Illustrations for Analytical Ultracentrifugation Data*, 1st ed.; Elsevier Inc., 2015; Vol. 562.

(8) Abagyan, R.; Totrov, M. *J. Mol. Biol.* **1994**, *235*, 983.

(9) Micco, M.; Collie, G. W.; Dale, A. G.; Ohnmacht, S. A.; Pazitna, I.; Gunaratnam, M.; Reszka, A. P.; Neidle, S. *J. Med. Chem.* **2013**, *56* (7), 2959.

(10) Wilson, T.; Costa, P. J.; Williamson, M. P.; Thomas, J. A. *J. Med. Chem.* **2013**.

(11) Chung, W. J.; Heddi, B.; Tera, M.; Iida, K.; Nagasawa, K.; Phan, A. T. *J. Am. Chem. Soc.* **2013**, *135* (36), 13495.

(12) Chung, W. J.; Heddi, B.; Hamon, F.; Teulade-Fichou, M.-P.; Phan, A. T. *Angew. Chem. Int. Ed. Engl.* **2014**, *53* (4), 999.

(13) Pfaendler, H. R.; Weimar, V. *Synthesis (Stuttg).* **1996**, *11*, 1345.

(14) Beghdadi, S.; Miladi, I. A.; Romdhane, H. Ben; Bernard, J.; Drockenmuller, E. *Biomacromolecules* **2012**, *13*, 4138.

(15) Kolmakov, K.; Wurm, C. A.; Meineke, D. N. H.; Göttfert, F.; Boyarskiy, V. P.; Belov, V. N.; Hell, S. W. *Chem. - A Eur. J.* **2014**, *20* (1), 146.

(16) Kotsuki, H.; Sugino, A.; Sakai, H.; Yasuoka, H. *Heterocycles* **2000**, *53* (11), 2561.

(17) Tagmose, T. M.; Bols, M. *Chem. - A Eur. J.* **1997**, *3* (3), 453.

(18) Park, S.; Shin, I. *Org. Lett.* **2007**, *9* (9), 1675.

(19) Takada, T.; Kawai, K.; Cai, X.; Sugimoto, A.; Fujitsuka, M.; Majima, T. *J. Am. Chem. Soc.* **2004**, *126* (4), 1125.
